# Genome-wide Identification and Expression Analysis of the CDPK Gene Family in Grape, *Vitis spp*

**DOI:** 10.1186/s12870-015-0552-z

**Published:** 2015-06-30

**Authors:** Kai Zhang, Yong-Tao Han, Feng-Li Zhao, Yang Hu, Yu-Rong Gao, Yan-Fei Ma, Yi Zheng, Yue-Jin Wang, Ying-Qiang Wen

**Affiliations:** State Key Laboratory of Crop Stress Biology for Arid Areas and College of Horticulture, Northwest A&F University, Yangling, 712100 Shaanxi People’s Republic of China; Key Laboratory of Horticultural Plant Biology and Germplasm Innovation in Northwest China, Ministry of Agriculture, Yangling, 712100 Shaanxi People’s Republic of China; Boyce Thompson Institute for Plant Research, Cornell University, Ithaca, NY 14853 USA

**Keywords:** Grapevine (*Vitis vinifera* L.) (*Vitis pseudoreticulata*), CDPK, Synteny analysis, Phylogenetic tree, Subcellular localization, expression profiles

## Abstract

**Background:**

Calcium-dependent protein kinases (CDPKs) play vital roles in plant growth and development, biotic and abiotic stress responses, and hormone signaling. Little is known about the *CDPK* gene family in grapevine.

**Results:**

In this study, we performed a genome-wide analysis of the 12X grape genome (*Vitis vinifera*) and identified nineteen *CDPK* genes. Comparison of the structures of grape *CDPK* genes allowed us to examine their functional conservation and differentiation. Segmentally duplicated grape *CDPK* genes showed high structural conservation and contributed to gene family expansion. Additional comparisons between grape and *Arabidopsis thaliana* demonstrated that several grape *CDPK* genes occured in the corresponding syntenic blocks of *Arabidopsis*, suggesting that these genes arose before the divergence of grapevine and *Arabidopsis*. Phylogenetic analysis divided the grape *CDPK* genes into four groups. Furthermore, we examined the expression of the corresponding nineteen homologous *CDPK* genes in the Chinese wild grape (*Vitis pseudoreticulata*) under various conditions, including biotic stress, abiotic stress, and hormone treatments. The expression profiles derived from reverse transcription and quantitative PCR suggested that a large number of *VpCDPKs* responded to various stimuli on the transcriptional level, indicating their versatile roles in the responses to biotic and abiotic stresses. Moreover, we examined the subcellular localization of VpCDPKs by transiently expressing six VpCDPK-GFP fusion proteins in *Arabidopsis* mesophyll protoplasts; this revealed high variability consistent with potential functional differences.

**Conclusions:**

Taken as a whole, our data provide significant insights into the evolution and function of grape CDPKs and a framework for future investigation of grape *CDPK* genes.

**Electronic supplementary material:**

The online version of this article (doi:10.1186/s12870-015-0552-z) contains supplementary material, which is available to authorized users.

## Background

Calcium (Ca^2+^), a universal second messenger in eukaryotes, mediates stimulus–response coupling in the regulation of diverse cellular functions [1, 2]. Various extracellular stimuli elicit specific calcium signatures that can be recognized by different calcium sensors. The three main classes of Ca^2+^ sensors identified in plants are: CaMs (calmodulins) and CaM-like proteins, CBLs (calcineurin B-like proteins), and CDPKs (calcium-dependent protein kinases) [3–5]. The CDPKs, also termed CPKs, consist of a variable N-terminal domain, a conserved serine/threonine kinase domain, an auto-inhibitory junction region, and a C-terminal regulatory CaM-like domain [6]. Unlike the other Ca^2+^ sensors, CDPKs have both Ca^2+^ sensing and responding activities due to their unique, CaM-like domain and protein kinase domain, which convert upstream Ca^2+^ signals into downstream phosphorylation events and cellular responses [7, 8].

Genome-wide analysis, together with comparative genomics, provides an effective way to understand the structures and functions of members of a gene family, using the insights gained from evolutionary relationships and experimental data. The original, systematic report on the *CDPK* gene family in *Arabidopsis thaliana* identified 34 *CDPK* gene family members [9], and was followed by research in rice (*Oryza sativa*) [10] and wheat (*Triticum aestivum*) [11]. Recently, genome-wide analyses of the *CDPK* gene family have been reported in maize (*Zea mays L*) [12] and poplar (*Populus trichocarpa*) [13]. Meanwhile, more and more investigations of *CDPK* genes have also involved horticultural plants, such as alfalfa [14], potato [15], strawberry [16], and tomato [17].

Work in *Arabidopsis* showed that CDPKs function in immune and stress signaling, growth and development, and hormone responses. AtCPKs play vital parts in immune signaling pathways; for example, AtCPK1 activates NADPH oxidase, resulting in an oxidative burst [18], and phosphorylates PAL (Phenylalanine ammonia-lyase) resulting in accumulation of salicylic acid (SA) [19]. AtCPK4/5/6/11 phosphorylates a specific subgroup of WRKY transcription factors, WRKY8/28/48, which participate in NLR-dependent restriction of pathogen growth. In addition, AtCPK5 activates RBOH to induce a reactive oxygen species (ROS) burst [20, 21]. CPKs also function in the response to abiotic stress. For example, *AtCPK*s help to enhance drought tolerance by responding to abscisic acid (ABA), leading to induction of expression of genes such as *AtCPK*4/*11* [22], and stomatal closure via induction of *AtCPK3/6* [23]. *AtCPK4/11* [22] and *AtCPK23* [24] trigger plant salt tolerance via controlling of K^+^ channels. In hormone signaling, studies have systematically examined ABA [25, 26], and a few studies have examined MeJA [27], SA [19], and ethylene [28]. The rich sequence and functional information from *Arabidopsis* enables us to extrapolate the functions of the orthologous genes in other species.

Grapevine is one of the most important fruit crops in the world. However, most cultivated grapevine varieties (*Vitis vinifera*) are susceptible to many pathogens and are sensitive to abiotic stresses. By contrast, Chinese wild grapevine (*Vitis pseudoreticulata*) accession Baihe-35-1 has demonstrated resistance to multiple diseases and to various environmental stresses [29, 30]. Previous examination of the *CDPK* gene family in *Vitis vinifera* identified 17 members [31], and in *Vitis amurensis* isolated 13 members [32]. However, prior work focused on evolutionary relationships with only a few transcriptional analyses in some tissues and developmental stages. In this study, we employed bioinformatics and publicly available data to identify and analyse grape *CDPK* genes on a genome-wide scale in *Vitis pseudoreticulata*, identifying two more members. Furthermore, we measured the expression of the *CDPK* genes (*VpCDPKs*) in Chinese wild grapevine *Vitis pseudoreticulata* accession Baihe-35-1 in response to various biotic and abiotic stresses as well as multiple phytohormone treatments. In addition, we showed that the VpCDPKs have different subcellular localizations when transiently expressed in *Arabidopsis* mesophyll protoplasts. More significantly, comparing *Arabidopsis* and grapevine *CDPK* gene structures, evolution, and experimental data provides insights on the functions of *VpCDPKs*. Our results provide a set of potential candidate *CDPK* genes for future genetic modification of pathogen resistance and stress tolerance in grapevine.

## Results

### Characteristics of grape *CDPK* genes

**Fig. 1:**
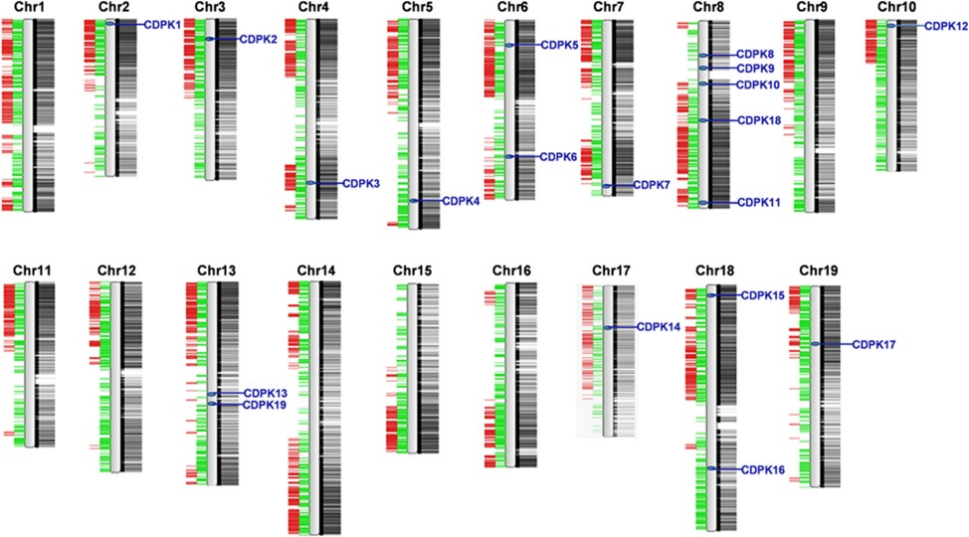
Chromosomal distribution of *CDPK* genes in the grape genome. The chromosome number is shown at the top of each chromosome. The positions of the grape *CDPK* genes are marked by blue lines on the chromosomes. The black lines and blocks indicate the coding sequence of the grape genome. The green lines show tandem duplications and the red lines show segmental duplications

We identified *CDPK* genes by searching the Pfam database and obtained the HMM (Hidden Markov Model) profiles of protein kinase domain Pkinase (PF00069) and EF-hand domain EF-hand_7 (PF13499). Then we used BLAST-P to search the 12X grapevine (*V. vinifera*) genome using Pkinase and EF-hand HMM profiles. We also performed BLAST-P searches at NCBI using full-length amino acid of putative grape *CDPK* genes. After that, we identified a bunch of candidates (data not shown). By removing incomplete gene sequences, transcripts of the same genes, and redundant sequences, we identified nineteen non-redundant *CDPK* genes in the grape genome (Table 1). Among those with alternative splice variants, we selected the longest variant for further analysis. The sequences of the nineteen grape CDPKs were submitted to InterPro and SMART databases to confirm their Pkinase domains and EF-hand domains. Previous work identified seventeen *CDPK* genes in grapevine [31]. The seventeen genes were named based on their distributions and relative linear orders on the respective chromosomes. On this basis, we added two members (GSVIVT01025745001 and GSVIVT01027353001) and named them *VvCDPK18* and *VvCDPK19* according to their chromosomal locations. The nineteen putative *CDPK* genes mapped on eleven grape chromosomes (Fig. 1). Among them, eight chromosomes possess one *CDPK* gene, three possess two *CDPK* genes, and Chr. 8 possesses five *CDPK* genes.

**Table 1:** Characteristics of Grape CDPK genes

Name	Locus ID^a^	Length (kb)	No. of aa	N-Term. aa^b^	N-Myr.^c^	N-Pal.^d^	No. of EFs^e^	Chr.	Group	Homologs in *Vp.* ^*f*^	Identity^g^ (%)
*VvCDPK1*	GSVIVT01019446001	9.175	528	MGNCNGLP	N	Y	4	2	II	*VpCDPK1*	-
*VvCDPK2*	GSVIVT01023866001	5.366	561	MGNTCRGS	N	Y	4	3	I	*VpCDPK2*	99.5
*VvCDPK3*	GSVIVT01018778001	6.278	558	MGACLSAT	Y	N	4	4	IV	*VpCDPK3*	99.5
*VvCDPK4*	GSVIVT01010743001	16.91	554	MGGCISMP	Y	Y	4	5	III	*VpCDPK4*	-
*VvCDPK5*	GSVIVT01025249001	6.444	518	MGNCCASP	N	Y	4	6	III	*VpCDPK5*	97.7
*VvCDPK6*	GSVIVT01037295001	3.103	534	MGNCCSQG	Y	Y	4	6	II	*VpCDPK6*	-
*VvCDPK7*	GSVIVT01000238001	7.807	540	MGMYTSKD	Y	N	4	7	I	*VpCDPK7*	-
*VvCDPK8*	GSVIVT01022524001	11.179	568	MGNTCVGP	N	Y	4	8	I	*VpCDPK8*	-
*VvCDPK9*	GSVIVT01022606001	3.947	580	MGNNCVGS	N	Y	4	8	I	*VpCDPK9*	99.3
*VvCDPK10*	GSVIVT01011167001	18.233	527	MGNCCRSP	N	Y	4	8	III	*VpCDPK10*	99.8
*VvCDPK11*	GSVIVT01033306001	5.504	526	MGNCCVTP	N	Y	4	8	III	*VpCDPK11*	100
*VvCDPK12*	GSVIVT01012730001	8.352	545	MGCFSSKE	Y	Y	4	10	II	*VpCDPK12*	-
*VvCDPK13*	GSVIVT01001931001	6.935	569	MGNTCVGP	N	Y	4	13	I	*VpCDPK13*	-
*VvCDPK14*	GSVIVT01008077001	9.065	552	MGNCIACV	Y	Y	4	17	III	*VpCDPK14*	-
*VvCDPK15*	GSVIVT01008749001	5.975	523	MGFCFSRP	Y	Y	4	18	II	*VpCDPK15*	-
*VvCDPK16*	GSVIVT01034489001	21.654	626	MVIKTKIS	N	N	4	18	I	*VpCDPK16*	-
*VvCDPK17*	GSVIVT01037652001	5.084	536	MGICLSKG	Y	Y	4	19	II	*VpCDPK17*	-
*VvCDPK18*	GSVIVT01025745001	8.344	575	MGLCQGKP	ND	Y	2	8	IV	*VpCDPK18*	-
*VvCDPK19*	GSVIVT01027353001	3.399	520	MGQETRRL	N	N	3	13	IV	*VpCDPK19*	99.8

^a^ IDs are available in the Grape Genome Browser (12X) (http://www.genoscope.cns.fr/externe/GenomeBrowser/Vitis/)

^b^ First eight amino acids at the N-terminal of the corresponding protein. The amino acids underlined indicate putative palmitoylation sites

^c^ The myristoylation sites were predicted by the Myristoylator program (http://web.expasy.org/myristoylator/). ND, not determined

^d^ The palmitoylation sites were predicted by CSS-Palm 3.0 (http://csspalm.biocuckoo.org/)

^e^ Number of EF-hands were predicted by InterPro (http://www.ebi.ac.uk/interpro/scan.html)

^f^ Homologous CDPK genes in Chinese wild grapevine *Vitis pseudoreticulata*

^g^ Sequence identity calculated with amino acid sequences using Vector NTI

Characteristics of the nineteen *CDPK* genes are shown in Table 1. The lengths of the gene sequences vary widely, from 3.103 to 21.654 kb, but the numbers of predicted amino acids are relatively similar for most genes, around 520 to 580, except VvCDPK16 with 626 amino acids. Fifteen of the nineteen CDPKs are predicted to be palmitoylated and eight of the nineteen CDPKs are predicted to be myristoylated. The predicted proteins for six CDPK genes have both N-terminal myristoylation sites and palmitoylation sites. Nine CDPK genes encode proteins with only palmitoylation sites and two (*VvCDPK*3 and *VvCDPK*7) have only myristoylation sites. Besides these, *VvCDPK16* and *VvCDPK19* have neither myristoylation nor palmitoylation sites. Typical CDPKs have four EF-hands, but not necessarily [9, 33]. The seventeen previously identified CDPKs were predicted to each have four EF-hands. In addition, the two new members, VvCDPK18 and VvCDPK19 were predicted to have two EF-hands and three EF-hands, respectively.

### Grape *CDPK* genes possess typical domains but complicated intron–exon organization

**Fig. 2:**
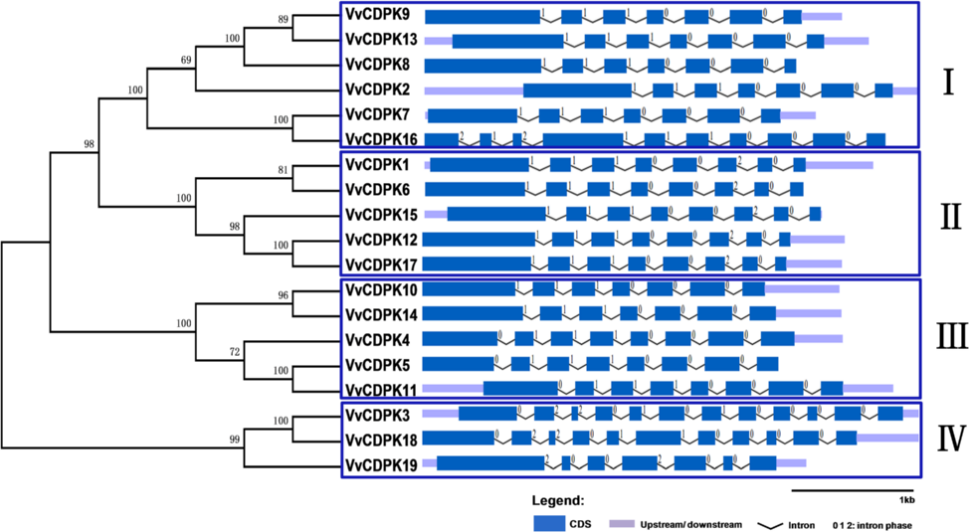
Phylogenetic relationships and intron-exon organization of grape CDPK genes. The unrooted phylogenetic tree was constructed using the full-length protein sequences of nineteen grape *CDPK* genes by the Neighbor-Joining method with 1,000 bootstrap replicates. The four subgroups are marked by square boxes and numbered with Roman numerals. The sizes of the exons are proportional to their sequence lengths

The predicted protein sequences of the nineteen grape CDPKs contain the four typical CDPK domains, including the N-terminal variable domain, the protein kinase domain, the junction domain, and the calmodulin-like domain (data not shown). The intron-exon organization can indicate the evolutionary relationships within multi-gene families [34]. As shown in Fig. 2, grape *CDPK* genes can be divided into four groups (I-IV) on the basis of the *Arabidopsis* classification [9]. Most of the grape *CDPK* genes have seven or eight exons, six or seven introns, and clear intron phase patterns. Group I members have seven exons, except *VvCDPK16* with ten exons, and their intron phases, with respect to the open reading frame, occur in the same pattern, with type-2 intron phases at the first three positions from the beginning and type-0 intron phases at the last three positions. All of the Group II members contain eight exons and also share the same intron phase pattern. Compared with the Group I members, Group II members have an additional exon. Group III can be divided into two subgroups. Subgroup 1 shares the same intron-exon organization as most of the Group I members, such as *VvCDPK10* and *VvCDPK14*, but subgroup 2 members, such as *VvCDPK4*, *VvCDPK5*, and *VvCDPK11*, have one more exon at the first position and one more type-0 phase intron. Compared with the other three groups, Group IV has a complicated intron-exon organization with differing numbers of exons and intron phases. Clustering the intron-exon structures of the nineteen *CDPK* genes by an unrooted phylogenetic tree suggests a connection between intron-exon structures and evolutionary relationships.

### Gene duplication and synteny analysis of grape *CDPK* genes

**Fig. 3:**
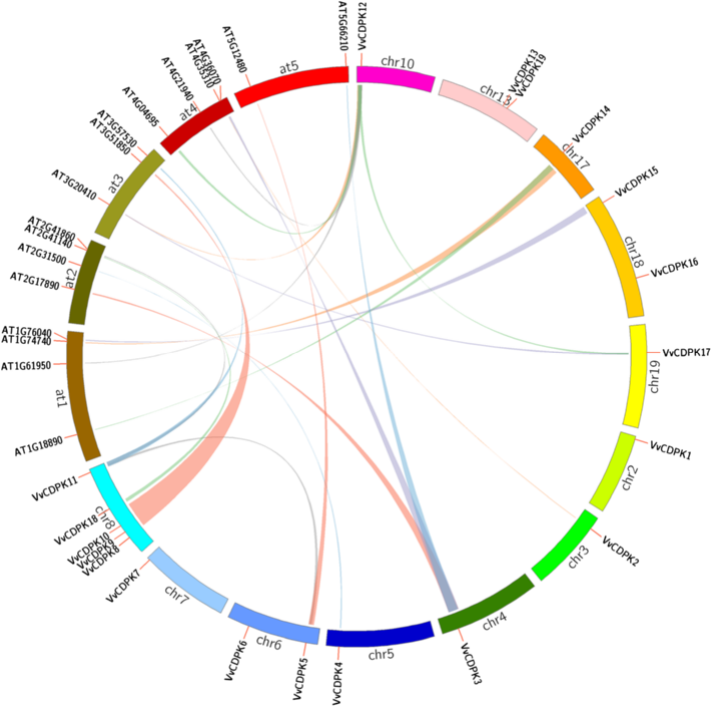
Segmental duplication of grape *CDPK* genes and synteny analysis of grape and *Arabidopsis CDPK* genes. Chromosomes of *V. vinifera* and *Arabidopsis* are shown in different colors and in circular form. The approximate positions of the *AtCDPK* and *VvCDPK* genes are marked with a short red line on the circle. Colored curves denote the syntenic relationships between grape and *Arabidopsis CDPK* genes

Gene duplication and divergence are important in gene family expansion [35] and in the evolution of novel functions [36]. Grapevine has undergone whole-genome duplications during its evolutionary history [37]. To examine the effect of duplications on the *CDPK* gene family, we obtained tandem duplication and segmental duplication gene pairs from PGDD (Plant Genome Duplication Database) and visualized them using Circos. In this study, we identified two segmental duplication pairs of grape *CDPK* genes (*VvCDPK5* and *VvCDPK11*, *VvCDPK12* and *VvCDPK17*) (Fig. 3) but did not find tandem duplication events among the grape *CDPK* genes.

To further explore the origin and evolution of grape *CDPK* genes, we investigated the syntenic relationship between grapevine and *Arabidopsis*. The synteny analysis showed that grape *CDPK* genes can be divided into two types (Fig. 3, Additional file 1. The first type of syntenic genes has a single grape gene that corresponds to a single *Arabidopsis* gene, including *VvCDPK2*-AT4G35310 (*AtCPK5*), *VvCDPK4*-AT2G31500 (*AtCPK24*), *VvCDPK5*-AT5G12480 (*AtCPK7*), *VvCDPK10*-AT3G51850 (*AtCPK13*), *VvCDPK15*-AT1G76040 (*AtCPK29*), and *VvCDPK17*-AT3G20410 (*AtCPK9*). The second type has a single grape *CDPK* gene that corresponds to multiple *Arabidopsis* genes, including *VvCDPK3*-AT2G17890 (*AtCPK16*)/AT4G36070 (*AtCPK18*)/ AT5G66210 (*AtCPK28*), *VvCDPK11*-AT2G41860 (*AtCPK14*)/ AT3G57530 (*AtCPK32*), *VvCDPK12*-AT1G61950 (*AtCPK19*)/ AT3G20410 (*AtCPK9*)/ AT4G04695 (*AtCPK31*)/ AT4G21940 (*AtCPK15*), and *VvCDPK14*-AT1G74740 (*AtCPK30*)/ AT1G18890 (*AtCPK10*). These results provide insights that will assist in prediction of the functions of grape CDPKs.

### Phylogenetic analysis of the grape *CDPK* genes

**Fig. 4:**
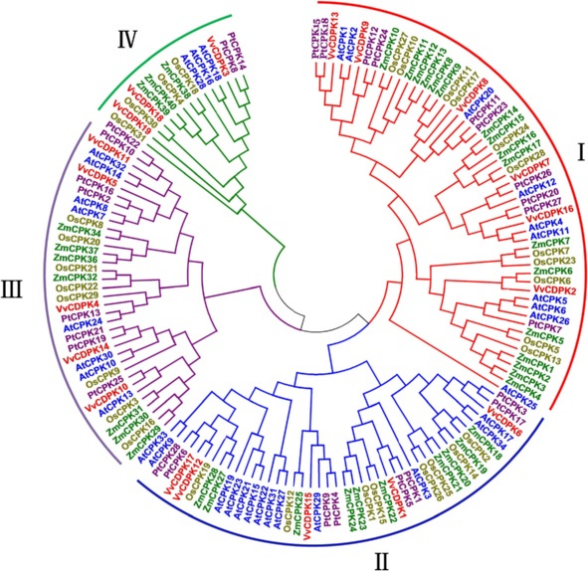
Phylogenetic analysis of grape *CDPK* genes. The full-length amino acid sequences of *CDPK* genes from grape (Vv, red), *Arabidopsis* (At, blue), *Populus trichocarpa* (Pt, purple), rice (Os, brown) and maize (Zm, green) were aligned by ClustalX and the phylogenetic tree was constructed using the Neighbor-Joining method with 1000 bootstrap replicates by MEGA 5.0. The four subgroups are marked with distinct colors and denoted by Roman numerals

To investigate the evolutionary relationships and functional associations, we constructed a neighbor-joining tree using the full-length amino acid sequences of CDPKs from grape, *Arabidopsis*, rice, maize, and poplar (Fig. 4, Additional file 2). The phylogenetic analysis indicated that nineteen *VvCDPKs* can be divided into four groups. Of the four groups, three groups appear to be well-defined, except Group IV, according to the distributions of branches. Most of the *CDPK* genes from monocots (rice and maize) clustered into one sub-branch, as did the *CDPK* genes from the eudicots (*Arabidopsis*, poplar and grape). Also, grape *CDPK* genes clustered more often with poplar *CDPK* genes than with *Arabidopsis CDPK* genes. For the four Groups of *VvCDPKs*, Group I contains six *VvCDPK* genes. Five of the six genes have homologs in *Arabidopsis* or poplar, or both, except *VvCDPK2*, which clustered with *ZmCPK6* and *OsCPK6*. Group II contains five grape *CDPK* genes and clustered into two subgroups. Among these genes, *VvCDPK12* and *VvCDPK17* show high sequence similarity with each other. Group III contains five grape *CDPK* genes and Group IV, the smallest group, contains three grape *CDPK* genes. These phylogenetic relationships suggest evolutionary conservation of the basal architecture of the CDPK family.

### Subcellular localization of grape CDPKs

Most CDPKs possess either N-myristoylation sites, S-palmitoylation sites, or both. These acylation sites are believed to be involved in targeting to membranes [9]. Because those acylation sites are predicted to be present in grape CDPKs (Table 1), to determine if grape CDPKs localize to membranes, we cloned six *VpCDPKs* from Chinese wild *Vitis pseudoreticulata* accession Baihe-35-1 and assessed the subcellular localizations of the encoded VpCDPKs by transient expression assays in *Arabidopsis* protoplasts, using translational fusions to GFP.

**Fig. 5:**
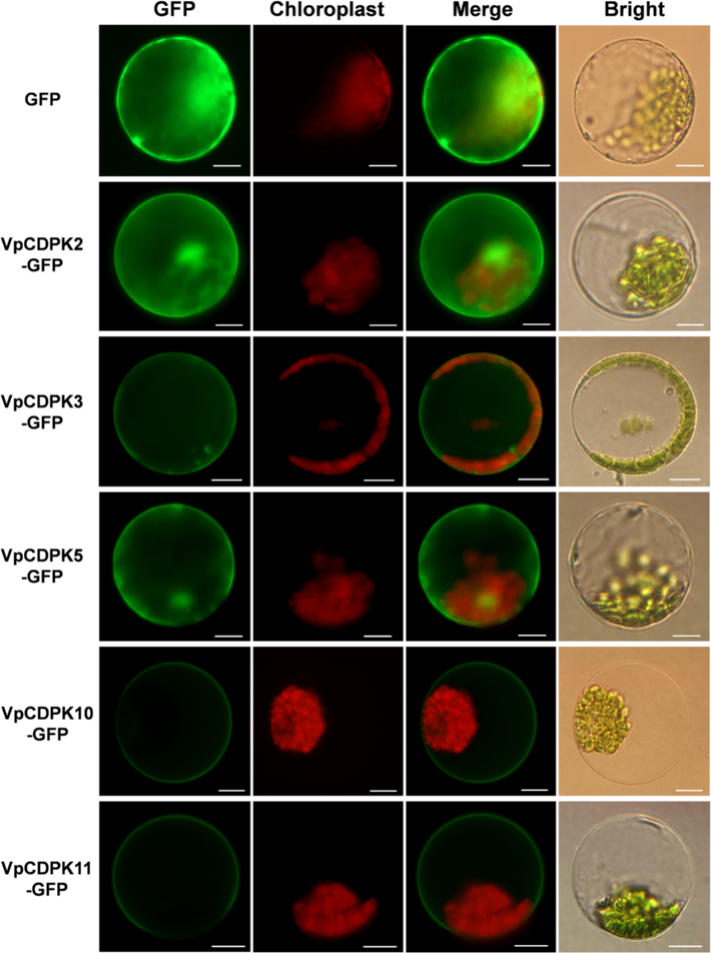
Subcellular localization of five VpCDPKs. The selected *CDPK* genes were cloned from Chinese wild grape (*Vitis pseudoreticulata*) and used to construct CaMV35S::CDPKs–GFP vectors in which GFP was fused at the C terminus. The five VpCDPK-GFP fusion proteins (VpCDPK2-GFP, VpCDPK3-GFP, VpCDPK5-GFP, VpCDPK10-GFP, and VpCDPK11-GFP) as well as GFP as the control, were transiently expressed in *Col-0 Arabidopsis* protoplasts and observed by fluorescence microscopy. The merged pictures include the green fluorescence channel (first panels) and the chloroplast autofluorescence channel (second panels). The corresponding bright field images are shown on the right. Bar = 5 μm

As shown in Fig. 5, five genes (*VpCDPK2*, *VpCDPK3*, *VpCDPK5*, *VpCDPK10*, and *VpCDPK11*) expressed VpCDPK-GFP fusion proteins in transformed *Arabidopsis* protoplasts. VpCDPK3-GFP, VpCDPK10-GFP, and VpCDPK11-GFP only localized on the plasma membrane. VpCDPK5-GFP localized on the plasma membrane and in the nucleus, and VpCDPK2-GFP localized in the nucleus and cytosol.

**Fig. 6:**
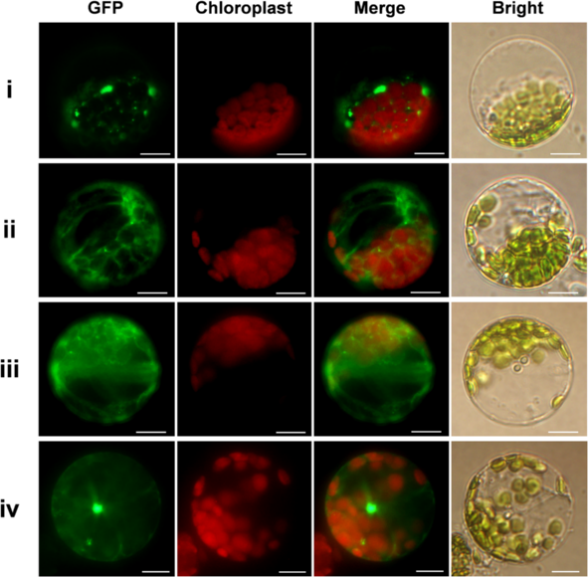
Subcellular localization of VpCDPK9. The Roman numerals (i-iv) represent the corresponding four subcellular localization patterns of VpCDPK9. *VpCDPK9* was cloned from Chinese wild grape (*Vitis pseudoreticulata*) and used to construct CaMV35S::VpCDPK9-GFP vectors in which GFP was fused at the C terminus. The VpCDPK9-GFP fusion protein, as well as GFP as a control, was transiently expressed in *Col-0 Arabidopsis* protoplasts and observed by fluorescence microscopy. The merged pictures include the green fluorescence channel (first panel) and the chloroplast autofluorescence channel (second panels). The corresponding bright field images are shown on the right. Bar = 5 μm

Unlike the other CDPKs, VpCDPK9 localized to four places (Fig. 6): (i) VpCDPK9 localized on some kind of plastids that could not be identified. Considering the closest gene in *Arabidopsis* is *AtCPK1*, which localizes to lipid bodies and peroxisomes [19, 38], the plastids that VpCDPK9-GFP localized in may well include lipid bodies and peroxisomes. (ii) VpCDPK9-GFP localized to the biomembrane system, most likely on the endoplasmic reticulum (ER), as well as on vesicles. (iii) VpCDPK9-GFP also showed extra fluorescence in the cytosol. (iv) We also detected VpCDPK9-GFP in the nucleus. The complexity of VpCDPK9 subcellular localization suggests its functional diversity and variety.

### Expression of grape *CDPK* genes in Chinese wild grape (*Vitis pseudoreticulata*)

Increasingly evidence shows that plant CDPKs function in responses to biotic and abiotic stress and signal transduction [9, 33]. To further test how grape *CDPK* genes respond to various stresses, we measured the expression of grape *CDPK* genes in Chinese wild grape (*Vitis pseudoreticulata*) accession Baihe-35-1.

#### Biotic stress

**Fig. 7:**
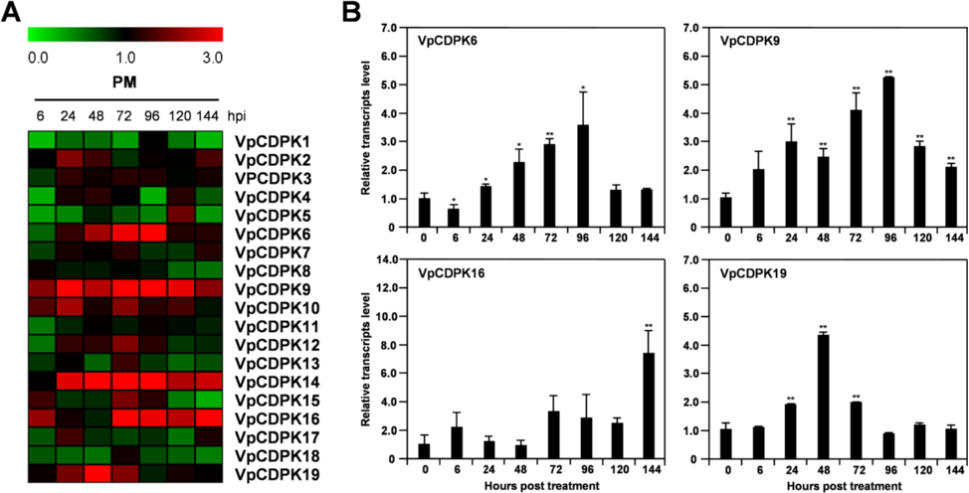
Expression of *CDPK* genes in Chinese wild grape (*Vitis pseudoreticulata*) during powdery mildew infection. Expression was measured by reverse transcription, followed by real-time, quantitative PCR, is indicated as fold-change of experimental treatments relative to control samples, and is visualized as heatmaps (A) and histogram (B). Grape *Actin1* (GenBank Accession number AY680701) was used as an internal control. The experiments were repeated three times and gave consistent results. Mean values and SDs were obtained from three biological and three technical replicates. (A) Expression profile of *VpCDPK* gene family under powdery mildew infection. The color scale represents log2 expression values, with red indicating increased transcript abundance and green indicating decreased transcript abundance. (B) Detailed expression levels of four *VpCDPK* genes that significantly up-regulated during powdery mildew infection. The data were showed as mean value ± SD. * and ** represent statistically significant (p < 0.05) or highly significant (p < 0.01), respectively

CDPKs act as vital sensors and responders in immune signaling [33]. In this study, we used RT-qPCR to perform a time course analysis of the transcript levels of *VpCDPKs* following inoculation of Chinese wild grape *Vitis pseudoreticulata* accession Baihe-35-1 with *Erysiphe necator*, the causal agent of grapevine powdery mildew (Fig. 7-a and Additional file 3). Five of the grape *CDPK* genes were up-regulated, including *VpCDPK6, VpCDPK9, VpCDPK14, VpCDPK16*, and *VpCDPK19*, with transcript levels that increased up to 3.0-fold (*p < 0.05*) but differed in response time and degree. For example, *VpCDPK9* transcript rapidly increased in abundance and reached a peak of 5.3-fold at 96 h post inoculation (hpi) (Fig. 7-b). Similarly, *VpCDPK14* transcript also rapidly increased and remained at high levels (Additional file 3). *VpCDPK6* (Additional file 3) and *VpCDPK19* (Fig. 7-b) shared similar expression patterns, with a slow increase in transcript levels to a peak and gradual plateau. *VpCDPK16* (Fig. 7-b) transcript abundance increased to nearly 3.3-fold, nearly instantly at 72 hpi, and remained at high levels. By contrast, several *CDPK* genes were down-regulated. For instance, *VpCDPK1* and *VpCDPK18* (Additional file 3) transcript levels decreased to 0.3- to 0.5-fold. These results demonstrate that several *CDPK* genes responded to powdery mildew inoculation in Chinese wild grapevine *V. pseudoreticulata* accession Baihe-35-1, suggesting their vital roles in immune signaling and responses.

#### Abiotic stress

Previous work showed that CDPKs participate in abiotic stress responses, including responses to salt and osmotic stress [22, 39] and to temperature stress [33]. In this study, we used NaCl treatment, and incubation at low or high temperature (4 °C or 42 °C) to understand how *VpCDPKs* responded to these abiotic stresses on the transcriptional level. Our expression data show consistency with the expression of *CDPK* genes in *Vitis vinifera* [31] and *Vitis amurensis* [32].

**Fig. 8:**
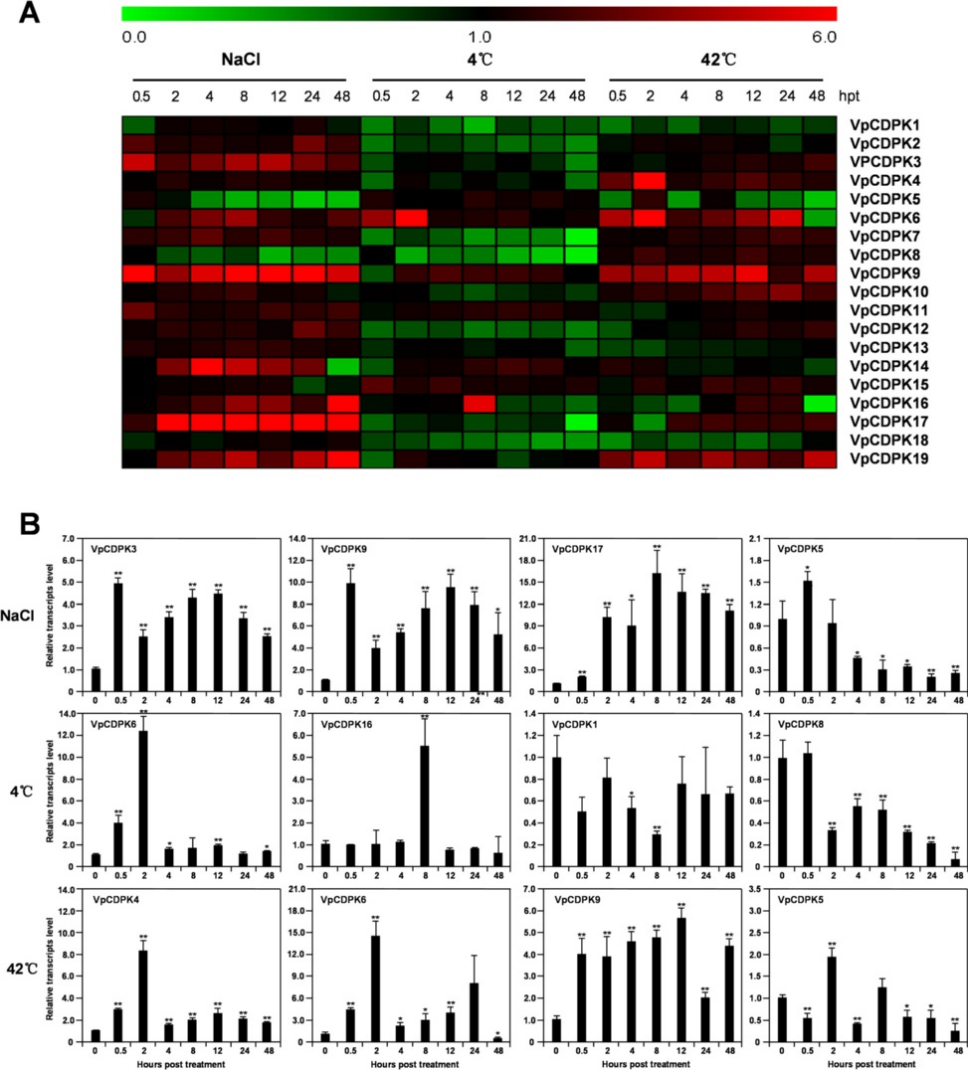
Expression of *CDPK* genes in Chinese wild grape (*Vitis pseudoreticulata*) under salt and temperature stress treatments. Expression was measured by reverse transcription, followed by real-time, quantitative PCR, is indicated as fold-change of experimental treatments relative to control samples, and is visualized as heatmaps **a** and histogram **b**. Grape *Actin1* (GenBank Accession number AY680701) was used as an internal control. The experiments were repeated three times and gave consistent results. Mean values and SDs were obtained from three biological and three technical replicates. **a** Expression profile of *VpCDPK* gene family under NaCl, 4 °C, and 42 °C treatments. The color scale represents log2 expression values, with red indicating increased transcript abundance and green indicating decreased transcript abundance. **b** Detailed expression levels of four VpCDPK genes showed unusual expression patterns under NaCl, 4 °C, and 42 °C treatments. The data were showed as mean value ± SD. * and ** represent statistically significant (p < 0.05) or highly significant (p < 0.01), respectively

As shown in Fig. 8 (see also Additional files 4, 5, 6), almost all nineteen *CDPK* genes were up- or down-regulated to at least one abiotic stress treatment, but some responded rather slightly. As a whole, the *VpCDPK* genes responded to NaCl treatment much more strongly than to temperature stress (Fig. 8-a). *VpCDPK6* and *VpCDPK9* transcript abundance increased in all three treatments, but none of the *CDPK* genes showed continuously low transcript levels in all treatments.

After NaCl treatment (Fig. 8 and Additional file 4), twelve of the nineteen *CDPK* genes were up-regulated, and six genes (*VpCDPK3, VpCDPK9, VpCDPK14, VpCDPK16, VpCDPK17*, and *VpCDPK19*) responded strongly with transcript levels increasing to 5.0- to 16-fold higher than the control sample (*p < 0.05*). *VpCDPK9*, the most rapidly responding gene, reached a peak of nearly 10-fold at 0.5 h post treatment (hpt) and rapidly decreased to 3.9-fold at 2 hpt, before its transcript levels increased from 3.9-fold to 9.7-fold at 2 h to 12 hpt (Fig. 8-b). The homologous gene of *VpCDPK9* in *Vitis amurensis*, *VaCPK1*, was also highly up-regulated under NaCl treatment [32]. In addition, *VpCDPK17* showed a peak of 16.3-fold at 8 hpt and increased transcript abundance of more than 9.0-fold from 2 to 48 hpt (Fig. 8-b), similar with the expression pattern of its homologs, *VaCPK9* [32] and *VvCPK17* [31]. We noted that the margins of treated leaves became dry from the outside to the inside and the plants had dry-dead leaves after 48 hpt, which might relate to the rapid down-regulation of *VpCDPK14* at 48 hpt. By contrast, *VpCDPK5* (Fig. 8-b) and *VpCDPK8* (Additional file 4) transcript abundance decreased after 2 hpt, indicating their probable negative regulatory roles. The *CDPK* genes that responded rapidly and strongly most likely participate in the NaCl stress response.

For 4 °C treatments (Fig. 8 and Additional file 5), only *VpCDPK6*, *VpCDPK9* and *VpCDPK16* (Fig. 8-a) showed up-regulation (*p < 0.05*). *VpCDPK6* was rapidly up-regulated, reached a peak of 12.4-fold at 2 hpt, and then declined to lower than 2.0- fold (Fig. 8-b). Moreover, *VpCDPK16* had a peak of 7.7-fold at 8 hpt but then fluctuated around 1.0-fold during the rest of the treatment time (Fig. 8-b). The *VpCDPK* genes with decreased transcript abundance might not participate in the signaling response to cold stress but their low activities might result in pervasive down-regulation of transcription. However, *VpCDPK8* (Fig. 8-b) was an exception, as its transcript levels decreased rapidly at 2 hpt and remained at a steady low level. Intriguingly, *VaCPK20,* as the homolog of *VpCDPK8* in *Vitis amurensis*, showed significant up-regulation under 10 °C treatment [32].

We also measured the transcript levels of *VpCDPK* genes in response to 42 °C treatment. We found that five *CDPK* genes (*VpCDPK4, VpCDPK6, VpCDPK9, VpCDPK10*, and *VpCDPK19*) significantly responded to 42 °C treatment (the peaks are over than 3.0- fold, *p < 0.05*); of these, *VpCDPK6* (Fig. 8-b) had a strong response at 2 hpt, with its transcript abundance increasing to 14.6-fold and remaining at a high level. The transcript abundance of *VpCDPK9* (Fig. 8-b) and *VpCDPK19* (Additional file 6) increased, similar to *VpCDPK6* (Fig. 8-b), although *VpCDPK6* transcripts were almost undetectable at 48 hpt. In particular, *VpCDPK4* (Fig. 8-b) responded to 42 °C treatment rapidly and intensely, reaching a peak of more than 8.3-fold and then returning to nearly normal transcript levels at 4 hpt. Taken together, our data showed that expression of *VpCDPKs* responded to low and high temperatures (4 °C and 42 °C) suggest that the *VpCDPKs* may play key roles in the response to temperature stress.

#### Hormone treatment

**Fig. 9:**
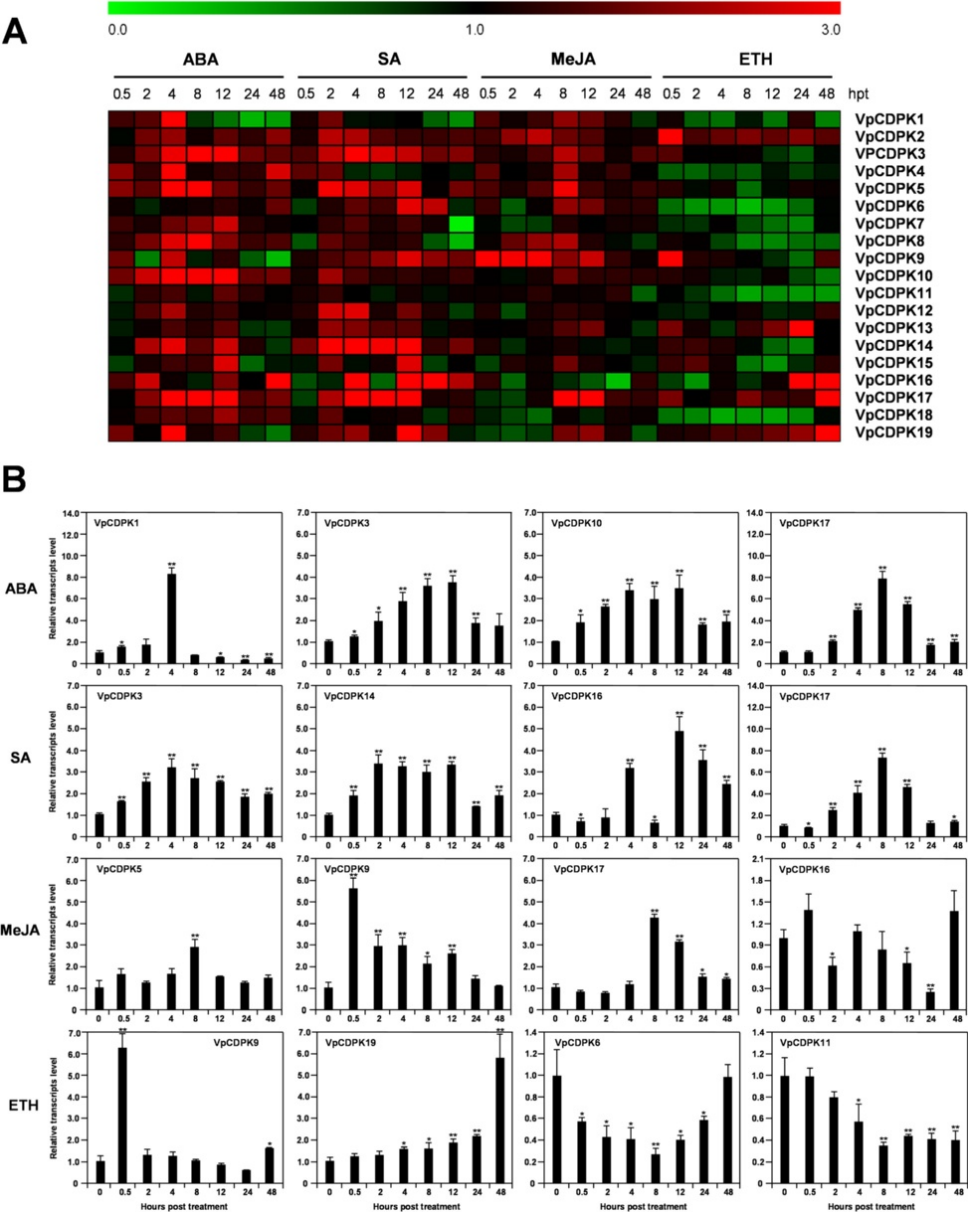
Expression of *CDPK* genes in Chinese wild grape (*Vitis pseudoreticulata*) in response to treatment with plant hormones. Expression was measured by reverse transcription, followed by real-time, quantitative PCR, is indicated as fold-change of experimental treatments relative to control samples, and is visualized as heatmaps (**a**) and histogram (**b**). Grape *Actin1* (GenBank Accession number AY680701) was used as an internal control. The experiments were repeated three times and gave consistent results. Mean values and SDs were obtained from three biological and three technical replicates. **a** Expression profile of *VpCDPK* gene family under ABA, SA, MeJA, and ethylene (ETH) treatments. The color scale represents log2 expression values, with red indicating increased transcript abundance and green indicating decreased transcript abundance. **b** Detailed expression levels of four VpCDPK genes showed unusual expression patterns under ABA, SA, MeJA, and ethylene (ETH) treatments. The data were showed as mean value ± SD. * and ** represent statistically significant (p < 0.05) or highly significant (p < 0.01), respectively

Plant hormones such as ABA, SA, MeJA, and ethylene have well-established roles in modulating plant signaling networks [40]. In this study, hormone treatments resulted in a wide variety of changes in the transcript levels of *VpCDPK* genes (Fig. 9 and Additional files 7, 8, 9, 10).

An increasing body of evidence has shown that CDPKs regulate ABA-mediated signal transduction in plants [22, 23, 41]. Our experimental data suggest that 16 of the 19 *VpCDPK* genes respond to ABA treatment (Fig. 9 and Additional file 7). Among them, eight *VpCDPK* genes (*VpCDPK1, 3, 4, 5, 10, 16, 17, 19*) showed significant up-regulation in response to ABA (the peaks are over than 3.0- fold, *p < 0.05*). However, *VpCDPK1* (Fig. 9-b) transcript levels rapidly increased up to 8.3-fold at 4 hpt and then decreased to lower than 0.3-fold at 8 hpt. Interestingly, the majority of the responsive *CDPK* genes showed a similar pattern, with their transcript abundance increasing rapidly to a peak at 4 hpt and remained at high levels until 12 hpt.

Similar to the ABA response, 13 of the 19 *VpCDPK* genes also showed positive regulation under SA treatment (Fig. 9 and Additional file 8). Six genes (*VpCDPK3, 5, 14, 16, 17, 19*) were significantly up-regulated (the peaks are over than 3.0-fold, *p < 0.05*), exhibiting a steady or gradual increase from 2 to 12 or 2 to 24 hpt. Interestingly, the expression of *VpCDPK6, VpCDPK9, VpCDPK14,* and *VpCDPK16* was also induced after inoculation with powdery mildew pathogen (Fig. 7). For instance, *VpCDPK9* showed positive regulation by inoculation of powdery mildew and peaked (~5.5-fold) at 96 hpi (Fig. 7-b), while under SA treatment, *VpCDPK9* was also up-regulated and peaked (~2.8-fold) at 12 hpt (Additional file 8). The expression pattern after SA treatment and powdery mildew inoculation, suggests the vital roles of grape *CDPK* genes in immune signaling.

In contrast with ABA and SA, the *VpCDPKs* showed more limited responses to MeJA treatment. A total of ten genes (*VpCDPK1, 2, 3, 4, 5, 6, 8, 9, 17, 19*) were up-regulated; and among them, only three *VpCDPK* genes (*VpCDPK5, 9, 17*) showed distinct up-regulation (the peaks are over than 3.0-fold, *p < 0.05*) (Fig. 9 and Additional file 9). In particular, their transcript abundance peaked at 4 or 8 hpt, except for *VpCDPK9* (Fig. 9-b), which strongly and rapidly responded to the treatment from 0.5 to 12 hpt with MeJA and peaked at 0.5 hpt. That might indicate its functional significance in the MeJA signaling pathway.

The expression changes caused by ethylene treatment (Fig. 9 and Additional file 10) were distinct from those caused by the other hormone treatments. For ethylene treatment, only a handful of *VpCDPK* genes (*VpCDPK2, 9, 13, 16, 17, 19*) were up-regulated (*p < 0.05*), while more genes showed down-regulation. For example, two up-regulated genes, *VpCDPK2* (Additional file 10) and *VpCDPK9* (Fig. 9-b), their transcript abundance increased rapidly at 0.5 hpt, but then increased only slightly or returned to normal transcript levels, respectively. Chinese wild grapevine Baihe-35-1 seems to be very sensitive to ethylene treatment and almost all the leaves of Baihe-35-1 plants fell at 48 hpt, so we had to use the fallen leaves for RNA extraction. *VpCDPK6, VpCDPK7, VpCDPK8, VpCDPK11*, and *VpCDPK18* had decreased transcript abundance lower than 0.5- fold during the treatment time, suggesting their potential negative regulatory functions in plant responses to ethylene signaling.

## Discussion

### Identification of Grape *CDPK* genes

Previously, Chen [31] investigated the *CDPK* gene family in grapevine and identified 17 members, which were predicted to have the typical CDPK gene structure with a Ser/Thr protein kinase domain and four EF-hands. Considering the complexity and variability in the calmodulin-like domain, we performed BLAST-P searches in NCBI using the putative grape *CDPK* genes predicted by Pkinase (PF00069) and EF-hand_7 (PF13499) HMM profiles, and identified the previously discovered 17 CDPKs and two more members (Fig. 1). Compared with *Arabidopsis* CDPKs [9] and rice CDPKs [10], we found that GSVIVT01025745001 (LOC100246588 in NCBI, designated here as *VvCDPK18*) possesses a similar domain organization to *AtCPK25* and *OsCPK30*, which are predicted to have no EF-hand with default parameters in Pfam but are predicted to have EF-hands in PROSITE and InterPro. In addition, the phylogenetic tree showing that GSVIVT01025745001 (*VvCDPK18*) and *OsCPK30* are homologous genes provides more evidence (Fig. 4). Therefore, we considered GSVIVT01025745001 (*VvCDPK18*) as members of the grape *CDPK* gene family and the same with GSVIVT01027353001 (LOC100250591 in NCBI, designated here as *VvCDPK19*). It is worth to mention that a homolog of *VvCDPK19* in *Vitis amurensis*, named as *VaCPK25*, has been identified as a *CDPK* gene [32]. Both of these two genes (*VpCDPK19* and *VaCPK25*) are same in gene length and phylogenetic relationship. Consider that, *VpCDPK19* can also be identified as a *CDPK* gene. These two new members of the grape *CDPK* gene family were designated *VvCDPK18* and *VvCDPK19* on the basis of their chromosomal locations.

### Structural characteristics of grape *CDPK* genes

The structural conservation and divergence of grape *CDPK* genes led to gene family expansion and functional conservation or differentiation. Structural characteristics such as acylation sites and intron-exon structures show the details of gene family expansion and divergence.

Most of CDPKs possess acylation sites, including N-myristoylation sites and S-palmitoylation sites, which are generally believed to function in membrane targeting [9]. In *Arabidopsis*, 21 *AtCPKs* have both myristoylation and palmitoylation sites and all of them target exclusively to membranes, especially to the plasma membrane, although several have not been determined [33]. Among our identified nineteen grape CDPK genes, six contain both myristoylation and palmitoylation sites, nine contain only palmitoylation sites, two contain only myristoylation sites, and two do not contain any acylation site (Table 1). We tested the subcellular localization of six VpCDPK proteins. VpCDPK2, VpCDPK5, VpCDPK10, and VpCDPK11 have palmitoylation sites while VpCDPK3 has a myristoylation site (Table 1) and they all localized on the plasma membrane (Fig. 5), consistent with results in *Arabidopsis* [33].

Three pivotal mechanisms contribute to gene family evolution and expansion: exon/intron gain or loss, exonization/ pseudo-exonization, and insertion /deletion [42]. Previous work reported that grape *CDPK* genes have a single origin and can be dated back to green algae, before plants colonized the land [43]. For the four groups in Fig. 2, Group IV was the earliest one that expanded from the evolutionary branch [31]. As a result, it has the longest evolutionary history, leading to complex intron-exon organization. Both Group II and Group III originated from Group I in evolutionary history, and show similar structural divergence. All of the members of Group II contain one more exon and a phase-2 intron at the sixth position, indicating that there was an intron insertion in the last exon, which contributed to the generation of Group II and expansion of the *VvCDPK* family. Group III can be separated into two subgroups in evolutionary history, as reflected in their intron-exon structures. One subgroup was similar to Group I in intron-exon structures and the other one contains one more exon and a phase-0 intron at the first position, suggesting that there was an intron insertion in the first exon. Our result is consistent with the previous work that can be reflected in the origin and evolutionary history of *VvCPKs* [31]. The intron-exon divergence was closely related to the evolutionary relationship of the grape CDPK family and might result in functional diversity.

### Evolutionary relationships of grape *CDPK* genes

To study the evolutionary relationships among different *VvCDPK* genes and the history of the *VvCDPK* gene family, as well as to further study their gene function, we investigated gene duplication events, syntenic regions, and phylogenetic relationships among the *VvCDPK* genes.

Segmental duplications and tandem duplications are the main mechanisms leading to gene family expansion [44]. These processes may lead to functional redundancy, sub-functionalization and neo-functionalization. No tandem duplication involving *VvCDPK* genes was discovered in the grapevine genome, but two segmental duplications were found, *VvCDPK5*-*VvCDPK11* and *VvCDPK12-VvCDPK17* (Fig. 3, Additional file 1). Previously, Chen identified three segmental duplications [31]. Except for *VvCDPK5*-*VvCDPK11* and *VvCDPK12*-*VvCDPK17*, they also identified triplet-duplicated genes, *VvCPK8-VvCPK9-VvCPK13*. However, the method they used was not clearly presented, so we cannot follow their method. Anyway, both of Chen and us indeed identified the two duplicated gene pairs, *VvCDPK5*-*VvCDPK11* and *VvCDPK12-VvCDPK17*. These two duplicated gene pairs are quite similar in gene length, acylation sites, and intron-exon organization (Table 1, Fig. 2). Furthermore, they possess the closest phylogenetic relationship among grape *CDPK* genes (Fig. 4). However, despite the fact *VvCDPK12* and *VvCDPK17* are phylogenetically closest among CDPKs from other species, these two genes have different syntenic genes in *Arabidopsis* (Fig. 3, Additional file 1), which means the duplication events happened before the evolutionary divergence of *Arabidopsis* and grapevine. Both *VvCDPK5* and *VvCDPK11* showed closer phylogenetic relationships with *Arabidopsis CDPK* genes than that with each other, indicating that this duplication event might also have happened before the divergence of *Arabidopsis* and grapevine. From what has been discussed above, *VvCDPK5-VvCDPK11* and *VvCDPK12-VvCDPK17* likely have different functions but still possess potential functional connections and similarities.

Comparative genomics approaches structure genomes into syntenic blocks that exhibit conserved features across the genomes [45]. The synteny analysis provides evolutionary and functional connections between grape and *Arabidopsis* syntenic genes. Furthermore, a large number of syntenic relationships suggest that some of the grape *CDPK* genes arose before the divergence of the *Arabidopsis* and grapevine lineages. Ten grape *CDPK* genes were found to have syntenic relationships with *Arabidopsis* genes (Fig. 3) All of these *VvCDPK* genes show close phylogenetic relationships with the corresponding *AtCDPK* genes (Fig. 4), suggesting their potential functional similarities. Interestingly, all of the grape *CDPK* genes in Group III (Fig. 4) have syntenic genes in *Arabidopsis*, suggesting that Group III CDPK genes are relatively conserved over evolutionary history.

Analysis of the phylogenetic tree revealed that CDPK homologs among several monocots and eudicots clustered into four distinct groups, which correspond to the clades formerly identified in green plants [43]. Previously, Hamel [43] examined the CDPK families from green algae to land plants to demonstrate that CDPK families are conserved among land plants, whereas CDPKs from green algae have continued to evolve independently. Supporting this, our data suggest that CDPKs from monocots and eudicots cluster separately (Fig. 4) and evolutionary relationships of the four clades are relatively conserved. The previous work [31] also constructed a phylogenetic tree that gives the consistent results and conclusions.

### Functional prediction of grape *CDPK* genes by comparison with *Arabidopsis*

Comparative genomics provides an effective way to understand the structure and function of genomes by translating knowledge gained from model species to the species of interest. Combining synteny analysis with phylogenetic analysis provides new insights for further investigating the functions of the grape *CDPK* genes by comparing orthologous genes between two species, in this case, between grapevine and *Arabidopsis*. Our study provided substantial evidence to help predict the functional conservation or divergence of *CDPK* genes between grapevine and *Arabidopsis*.

Chinese wild grape (*Vitis pseudoreticulata*) accession Baihe-35-1, compared with the sequenced *Vitis vinifera*, reported to have remarkable resistance to both biotic and abiotic stress [29, 30]. In addition, *Vitis amurensis* is also a wild grapevine species with a high level of resistance to multiple stresses [32]. Our expression profile of *VpCDPK* genes showed consistency with that reported in *Vitis vinifera* [31] and *Vitis amurensis* [32]. For example, the homologous *CDPK* genes, *VpCDPK3/VvCPK3/VaCPK16* and *VpCDPK17/VvCDPK17/VaCPK17* showed distinct up-regulation under salt stress treatment [31, 32] (Fig. 8-b). However, there are also some differences on genes expression, e.g., *VaCPKs* respond to cold stress much significantly than *VpCDPKs*, probably because of *Vitis amurensis* shows strong capacity of cold-resistance.

Two duplicated gene pairs, *VpCDPK5*/*VpCDPK11* and *VpCDPK12*/*VpCDPK17*, as discussed above, provide experimental evidence to support our predictions and views. *VpCDPK5* and *VpCDPK11* each have particular syntenic genes in *Arabidopsis* showing a complicated evolutionary history. In addition, the experimental data showed that these two genes have distinct expression patterns under a range of treatments. For example, *VpCDPK5* was obviously up-regulated under hormone treatments (except ethylene), while *VpCDPK11* was only slightly up-regulated under ABA treatment (Fig. 9 and Additional files 7, 8, 9, 10). Also, for abiotic stress treatments, *VpCDPK5* was down-regulated under NaCl and 42 °C treatments, but *VpCDPK11* transcripts remained at constant levels (Fig. 8 and Additional files 4, 6). Furthermore, subcellular localization analysis demonstrated that VpCDPK5-GFP localized on plasma membrane and in the nucleus, but VpCDPK11-GFP only localized on plasma membrane (Fig. 5). The aforementioned information indicated that *VpCDPK5* and *VpCDPK11* might well have undergone neo-functionalization. Moreover, *VvCDPK11* in *V. vinifera* cv. Corvina expressed in almost all of the organs, whereas *VvCDPK5* only expressed in pollen [31]. However, we tested *VpCDPK5* in the Chinese wild grape *V. pseudoreticulata* responded to hormone treatments (Fig. 9) in leaves, suggesting that *VpCDPK5* was not only transcribed in pollen. For *VpCDPK11*, its low expression under multiple treatments (Figs. 7, 8, 9) and high expression in almost all tissues [31] indicates that its function most likely relates to housekeeping genes. The other duplicated gene pair, *VpCDPK12* and *VpCDPK17* were quite different. The transcript levels of these two genes showed similar tendencies under abiotic stress, ABA and SA treatments (Figs. 8, 9 and Additional files 4, 5, 6, 7, 8), however, *VpCDPK17* seemed to respond much strongly than *VpCDPK12*. Based on Chen [31], *VvCDPK17* had high transcript levels in almost all tissues while *VvCDPK12* had low transcript levels. These data suggest that the duplicated gene pair *VpCDPK12/VpCDPK17* probably have undergone sub-functionalization and *VpCDPK12* tends to be non-functional.

It is worth noting that *VpCDPK9* is unique in the grape *CDPK* family. For one thing, it showed an unusual expression pattern under various treatments, being strongly up-regulated, except for 4 °C treatment (Fig. 7, 8, 9). Its transcript abundance increased to a high level during most or all of the treatment (Figs. 7, 8, 9). Subcellular localization also demonstrated its considerable differences with the other grape CDPKs we examined. The other VpCDPKs have only one pattern of localization (Fig. 5), while VpCDPK9 shows four patterns, including in some kind of plastids, in biomembranes, in the cytosol, or in the nucleus (Fig. 6). Testing why VpCDPK9 has four patterns of localization, and the relationship between its localization and function, will require more experimental data. The genes that are phylogenetically close to *VpCDPK9* in *Arabidopsis* are *AtCPK1* and *AtCPK2* (Fig. 4). *VpCDPK9* has structural and functional commonalities with *AtCPK1*. For example, *AtCPK1* expression is rapidly induced by fungal elicitors and loss-of-function mutants of *AtCPK1* exhibit higher susceptibility to pathogen infection [19]. Ectopic expression of *AtCPK1* enhanced NADPH oxidase activity and the oxidative burst in tomato protoplasts [18]. Cold-stress can also induce *AtCPK1* transcripts via phosphoprotein signals [46]. Our data also showed that *VpCDPK9* not only responded to powdery mildew infection and abiotic stress, but also responded to hormone treatments (Figs. 7, 8, 9). These results are consistent with the expression profile of *AtCPK1*. Future work will be directed toward identification of potential defense components that may be directly or indirectly regulated by VpCDPK9 in grapevine Baihe-35-1.

*VpCDPK2*, whose *Arabidopsis* homolog is *AtCPK5*, is found in the chromosomal region syntenic with *AtCPK5*, and plays positive regulatory roles in various treatments (Figs. 7, 8, 9). Results on *AtCPK5* show high consistency with our results on *VpCDPK2*. (i) *AtCPK5* was reported to localize to the plasma membrane and the nucleus [47, 48], consistent with our data showing that *VpCDPK2* also localized to the plasma membrane and nucleus (Fig. 5). (ii) *AtCPK5* can localize to the nucleus where it interacts with and phosphorylates WRKY8, 28, and 48 to activate defense genes [48], while VpCDPK2 also localized in the nucleus (Fig. 5), suggesting it has similar functions with AtCPK5. (iii) AtCPK5 also phosphorylates RBOHD in vivo, resulting in H_2_O_2_ production and leading to cell death [21, 49], involving in immune signaling. *VpCDPK2* responded to powdery mildew inoculation with up-regulated transcript levels (Fig. 7), indicating its potential function in immune signaling and that *VpCDPK2* most likely participates in the analogous pathways to those mentioned above for *Arabidopsis*. These structural and locational similarities provide indications of functional consistency.

*VpCDPK10* has a syntenic gene in *Arabidopsis*, *AtCPK13*. Further analysis provides valuable comparisons of these two genes and helps predict the function of *VpCDPK10*. In our study, *VpCDPK10* localized on the plasma membrane (Fig. 5), and *AtCPK13* was also reported to localize on the plasma membrane [50]. *VpCDPK10* expression did not respond to stress treatments but was distinctly up-regulated in response to ABA treatment (Fig. 8). AtCPK13 specifically inhibits KAT1 and KAT2 shaker channels [50], which participate in ABA-induced stomatal movements by affecting CDPK phosphorylation [41]. These insights indicate that *VpCDPK*10 plays important roles in ABA- and Ca^2+^-mediated stomatal regulation.

Another intriguing gene, *VpCDPK16*, is phylogenetically close to *AtCPK4* and *AtCPK11* (Fig. 4), which are activated by ABA and phosphorylate the C-terminus of ACS6 in ethylene biosynthesis [28]. Meanwhile, AtCPK4 and AtCPK11 can phosphorylate the ABA-responsive transcription factors ABF1 and ABF4 and lead to salt insensitivity in seed germination and decrease tolerance of seedlings to salt stress [22]. Sustained *AtCPK4* and *AtCPK11* activation directly phosphorylated WRKY transcription factors involved in immune signaling [51]. Expression patterns suggest that *VpCDPK16* responded to powdery mildew inoculation as well as NaCl, ABA, and SA treatments (Figs. 7, 8, 9), showing high functional consistency with *AtCPK4* and *AtCPK11*. This evidence indicates that *VpCDPK16* might participate in similar pathways as *AtCPK4* and *AtCPK11* in *Arabidopsis*.

In addition to stress, *CDPK* genes also play important roles in growth and development process. On this regard, plant hormones are mainly responsible. For instance, several plant hormones may play central roles in the control of ripening in the grape berry [52]. As a support, Kühn et al. [53] investigated the aminocyclopropane-1-carboxylate (ACC) synthases genes in first developed grape berries, which pathway is related to ethylene signaling. For *CDPK* genes, several genes including *AtCPK16* [54], *NtCDPK2* [55] and *LeCDPK2* [56] were reported to phosphorylate ACC synthases to trigger ethylene biosynthesis and accumulation. Coincidence is that *VpCDPK9* and *VpCDPK13*, as homologs of *NtCDPK2* and *LeCDPK2*, are positively regulated under ethylene treatment, indicating their possible functions involved in ethylene signaling. However, *CDPKs* functions in plants growth and development process still need more experimental evidence to deeply understand their biological roles and the pathways they involved in.

## Conclusions

So far, little systematic analysis of the *CDPK* family has been reported in grapevine, and the functions of most *CDPK* genes remain unclear. However, accumulating evidence indicates that CDPKs play important roles in response to a broad variety of abiotic and biotic stresses and biological processes. In this paper, genome-wide identification, evolutionary, and expression analyses were carried out to provide a framework for further analysis of grape *CDPK* genes in defining their biological functions and pathways during stress responses as well as growth and development. Expression profiles showed grape *CDPK* genes respond to various stresses and hormone treatments; moreover, analysis of CDPK subcellular localization gave evidence as to their functions. Comparisons of the grape and *Arabidopsis* genomes and expression profiles provide novel insights into the functions of less well-studied genes according to their better-understood homologs. By prediction and experimental data, we speculated that *CDPK* gene family might participate in responses to pathogen, cold, heat and salt stress, and the related biological processes might covering the regulation of gene expression, control of the ion channel, regulation of the enzyme activity, mediation of the cross-talk between signaling pathways and so on. These observations may lay the foundation for future functional analysis of grape *CDPK* genes to unravel their biological roles.

## Methods

### Identification of grape *CDPK* genes

To identify the *CDPK* genes in grapevine, we downloaded the Hidden Markov Model (HMM) profiles of the core protein kinase domain (PF00069) and EF-hand domain (EF-hand_7, PF13499) from Pfam database (http://pfam.xfam.org/). Then we performed a BLAST-P search in the Grape Genome Database (12X) (http://www.genoscope.cns.fr/externe/GenomeBrowser/Vitis/) using the HMM profiles as queries with e-value of 0.01. We also performed BLAST-P searches at NCBI using full-length amino acid sequences of the primarily identified grape *CDPK* genes and chose the candidates of e-value lower than 1e^−60^. All putative *CDPK* genes were manually verified with the InterProScan program (http://www.ebi.ac.uk/Tools/pfa/iprscan/) to confirm their completeness and existence of the core domains. Among those with alternative splice variants, we selected the longest variant for further analysis. Sequences of *Arabidopsis*, rice, maize, and poplar *CDPK* genes were obtained from the *Arabidopsis* Information Resource (TAIR, https://www.arabidopsis.org/), rice genome database in TIGR (http://rice.tigr.org), maize genome database (http://www.maizesequence.org/index.html), and Phytozome (http://www.phytozome.net/), respectively.

### Chromosomal localization and synteny analysis

Grape *CDPK* genes were mapped to chromosomes by identifying their chromosomal locations, as obtained from the Grape Genome Database (12 X) (http://www.genoscope.cns.fr/externe/GenomeBrowser/Vitis/) and NCBI Map Viewer (http://www.ncbi.nlm.nih.gov/mapview/). The segmental and tandem duplication regions, as well as chromosomal location, were established using PLAZA v3.0 Dicots (http://bioinformatics.psb.ugent.be/plaza/versions/plaza_v3_dicots/). For synteny analysis, synteny blocks within the grape genome and between grape and *Arabidopsis* genomes were downloaded from the Plant Genome Duplication Database and visualized using Circos (http://circos.ca/).

### Gene structure and phylogenetic analysis

Myristoylation and palmitoylation sites were predicted by Myristoylator (http://web.expasy.org/myristoylator/) and CSS-Palm 3.0 (http://csspalm.biocuckoo.org/), respectively. The intron-exon organization analysis was carried out using GSDS 2.0 (http://gsds.cbi.pku.edu.cn/) by alignment of the cDNA sequences with their corresponding genomic DNA sequences, and the results were consistent with the phylogenetic analysis. Multiple alignments of the identified grape CDPK amino acid sequences were performed using ClustalX. The phylogenetic tree was constructed with MEGA5.0 using the Neighbor-Joining method and the bootstrap test carried out with 1,000 replicates [57].

### Plant material and treatments

The Chinese wild grapevine *V. pseudoreticulata* accession Baihe-35-1 was grown in the grapevine germplasm resources greenhouse of Northwest A&F University in China, at temperatures of 22 to 27 °C, relative humidity of 60 to 80 %, and without supplemental lighting. When shoots of vines were 40–50 cm in length, the third and fourth fully expanded young grapevine leaves beneath the apex were selected for treatments. Plants of *Arabidopsis thaliana* ecotype Col-0 were grown at 22 °C, 75 % relative humidity, and under short-day (8 h light at 125 μmol · m^−2^ · s^−1^, 16 h dark) conditions for 4 to 5 weeks before transformation.

A grapevine powdery mildew (*Erysiphe necator)* isolate NAFU1 (GenBank accession no. KJ539202) was collected from a vineyard in Northwest China, and maintained on the leaves of *V. vinifera* cv. Thompson seedless, which was soil-grown in pots. The pathogen was sub-cultured onto fresh leaves every twenty days. The leaves of Baihe-35-1 were inoculated by touching the adaxial epidermis with sporulating colonies on the surface of the ‘Thompson Seedless’ infected leaves. Plants were then incubated in the greenhouse. Inoculated leaves were collected at 0, 6, 24, 48, 72, 96, 120, and 144 h post-inoculation (hpi). Inoculations were repeated three times.

For salt stress treatments, four-month-old soil-grown plants were irrigated with 300 mM NaCl. Treated leaves were collected at 0, 0.5, 2, 4, 8, 12, 24, and 48 h post-treatment (hpt). For cold treatment, plants were first grown at 22 °C, and then transferred to 4 °C, and treated leaves were collected at 0, 0.5, 2, 4, 8, 12, 24, and 48 h post-treatment (hpt). For high temperature treatment, plants were first grown at 22 °C, and then transferred to 42 °C, and treated leaves were collected at 0, 0.5, 2, 4, 8, 12, 24, and 48 h post-treatment (hpt). The leaves of Baihe-35-1 were sprayed with solution of 0.1 mM abscisic acid (ABA), 1 mM salicylic acid (SA), 0.1 mM methyl jasmonate (MeJA), or 0.5 g/L ethephon (Eth), and then collected for RNA isolation. The treated leaves were collected at 0, 2, 4, 8, 12, 24, and 48 h post-treatment (hpt). Another set of control plants were similarly treated with distilled water. All plants were treated in the light and three independent experiments were performed.

### Reverse transcription quantitative PCR

Total RNA of grape leaves was extracted using the E.Z.N.A. Plant RNA Kit (Omega, Guangzhou, China) according to the manufacturer’s instructions. First-strand cDNA was synthesized from 2 μg total RNA using PrimeScript RTase (Takara, Dalian, China). Quantitative PCR (qPCR) was carried out using SYBR green (Takara, Dalian, China) on an IQ5 real time PCR machine (Bio-Rad, Hercules, CA, USA) according to the manufacturer’s instructions. Thermal cycling consisted of a hold at 95 °C for 30 s, followed by 40 cycles of 95 °C for 30 s and 58 °C for 30 s. After amplification, samples were kept at 50 °C for 1 min and the temperature was raised gradually by 0.5 °C every 10 s to perform the melt-curve analysis. Grape *VpActin* (accession no. AY680701) was amplified as an internal control. All reactions were performed in triplicate in each experiment and three biological repeats were conducted. Primers used for RT-qPCR are listed in Additional file 11. Each relative expression level was analyzed with IQ5 software using the Normalized Expression method (2^-△△CT^ method). Expressional data consist of three replicated treatments and controls, which were calculated as 2-log-based values and were divided by the control.

### Statistical analysis

The statistical analysis was performed with the SPSS 19.0 software. The data were showed as mean value ± SD. We examined the homoscedasticity of our data by F-test. The significance of the differential expression between treatments and controls (0 hpt) was verified by performing Student’s *t*-test. p < 0.05 and p < 0.01 were taken as statistically significant or highly significant, respectively. The biological significance of RPKM was set as a fold change greater than 2-fold or less than 0.5-fold.

### Subcellular localization of VpCDPK

The predicted full-length coding sequences of grape *VpCDPK* genes, including *VpCDPK2*, *VpCDPK3*, *VpCDPK5*, *VpCDPK9*, *VpCDPK10*, and *VpCDPK11*, were amplified by high-fidelity Taq HS-mediated PCR from cDNA of the Chinese wild grapevine *V. pseudoreticulata* accession Baihe-35-1 leaves. The amplified PCR products were digested with *Sal*I and *Xho*I and fused in-frame with *GFP* in the *Sal*I and *Xho*I site of the pBI221 vector containing the CaMV *35S* promoter (Clontech, Beijing, China) resulting in plasmids pVpCDPKs-GFP. Primers used for cloning genes and for constructing vectors are shown in Additional file 11.

For transient expression of VpCDPKs-GFP in *Arabidopsis* mesophyll protoplasts, DNA of the corresponding pVpCDPK-GFP plasmids was transformed into Col-0 leaves using a previously described method [58]. After transformation, Col-0 leaves were kept in darkness at room temperature for 16–18 h before examination by fluorescence microscopy. Images were acquired using an Olympus BX-51 inverted fluorescence microscope (Olympus, Japan). The image data were processed using Adobe Photoshop (Mountain View, CA, USA). All transient expression assays were repeated at least three times.

### Availability of supporting data

Phylogenetic data (alignments and phylogenetic trees) supporting the results of this article have been deposited in TreeBASE respository and is available under the URL http://purl.org/phylo/treebase/phylows/study/TB2:S17752.

Sequence data of the isolated *VpCDPK* genes in this article can be found in GenBank (http://www.ncbi.nlm.nih.gov) under the accessions of KR153945- KR153946 and KR153948- KR153951.

## Additional files

Additional file 1:
**The syntenic relationships among grape and** ***Arabidopsis CDPK*** **genes.**
Additional file 2:
**The CDPKs amino acid sequences used to construct the phylogenetic tree.**
Additional file 3:
**Detailed expression profiling of the remaining 15** ***VpCDPK*** **genes during powdery mildew infection. Detailed expression levels were measured by RT-qPCR.** Grape *Actin1* (GenBank Accession number AY680701) was used as an internal control. The experiments were repeated three times and gave consistent results. Mean values and SDs were obtained from three biological and three technical replicates. The data were showed as mean value ± SD. * and ** represent statistically significant (p<0.05) or highly significant (p<0.01), respectively. Additional file 4:
**Detailed expression profiling of the remaining 15** ***VpCDPK*** **genes under NaCl treatment.** Detailed expression levels were measured by RT-qPCR. Grape *Actin1* (GenBank Accession number AY680701) was used as an internal control. The experiments were repeated three times and gave consistent results. Mean values and SDs were obtained from three biological and three technical replicates. The data were showed as mean value ± SD. * and ** represent statistically significant (p<0.05) or highly significant (p<0.01), respectively. Additional file 5:
**Detailed expression profiling of the remaining 15** ***VpCDPK*** **genes under 4°C treatment.** Detailed expression levels were measured by RT-qPCR. *Actin1* (GenBank Accession number AY680701) was used as an internal control. The experiments were repeated three times and gave consistent results. Mean values and SDs were obtained from three biological and three technical replicates. The data were showed as mean value ± SD. * and ** represent statistically significant (p<0.05) or highly significant (p<0.01), respectively. Additional file 6:
**Detailed expression profiling of the remaining 15** ***VpCDPK*** **genes under 42°C treatment.** Detailed expression levels were measured by RT-qPCR. *Actin1* (GenBank Accession number AY680701) was used as an internal control. The experiments were repeated three times and gave consistent results. Mean values and SDs were obtained from three biological and three technical replicates. The data were showed as mean value ± SD. * and ** represent statistically significant (p<0.05) or highly significant (p<0.01), respectively. Additional file 7:
**Detailed expression profiling of the rest remaining** ***VpCDPK*** **genes under ABA treatment.** Detailed expression levels were measured by RT-qPCR. *Actin1* (GenBank Accession number AY680701) was used as an internal control. The experiments were repeated three times and gave consistent results. Mean values and SDs were obtained from three biological and three technical replicates. The data were showed as mean value ± SD. * and ** represent statistically significant (p<0.05) or highly significant (p<0.01), respectively. Additional file 8:
**Detailed expression profiling of the remaining 15** ***VpCDPK*** **genes under SA treatment.** Detailed expression levels were measured by RT-qPCR. *Actin1* (GenBank Accession number AY680701) was used as an internal control. The experiments were repeated three times and gave consistent results. Mean values and SDs were obtained from three biological and three technical replicates. The data were showed as mean value ± SD. * and ** represent statistically significant (p<0.05) or highly significant (p<0.01), respectively. Additional file 9:
**Detailed expression profiling of the remaining 15** ***VpCDPK*** **genes under MeJA treatment.** Detailed expression levels were measured by RT-qPCR. *Actin1* (GenBank Accession number AY680701) was used as an internal control. The experiments were repeated three times and gave consistent results. Mean values and SDs were obtained from three biological and three technical replicates. The data were showed as mean value ± SD. * and ** represent statistically significant (p<0.05) or highly significant (p<0.01), respectively. (TIFF 4982 kb) Additional file 10:
**Detailed expression profiling of the remaining 15** ***VpCDPK*** **genes under ethylene treatment.** Detailed expression levels were measured by RT-qPCR. *Actin1* (GenBank Accession number AY680701) was used as an internal control. The experiments were repeated three times and gave consistent results. Mean values and SDs were obtained from three biological and three technical replicates. The data were showed as mean value ± SD. * and ** represent statistically significant (p<0.05) or highly significant (p<0.01), respectively. Additional file 11:
**Primers used in this study.**

## Abbreviations

CDPK: Calcium-dependent protein kinase
RT-qPCR: Reverse transcription-quantitative polymerase chain reaction
GFP: Green fluorescent protein
ABA: Abscisic acid
SA: Salicylic acid
MeJA: Methyl jasmonate
ETH: Ethylene
PM: Powdery mildew
Hpi: Hours post inoculation
Hpt: Hours post treatment

## References

[1] Sanders D, Brownlee C, Harper JF (1999). Communicating with calcium. Plant Cell.

[2] Kudla J, Batistic O, Hashimoto K (2010). Calcium Signals: The Lead Currency of Plant Information Processing. Plant Cell.

[3] Snedden WA, Fromm H (1998). Calmodulin, calmodulin-related proteins and plant responses to the environment. Trends Plant Sci.

[4] Luan S, Kudla J, Rodriguez-Concepcion M, Yalovsky S, Gruissem W (2002). Calmodulins and calcineurin B-like proteins: Calcium sensors for specific signal response coupling in plants. Plant Cell.

[5] Sanders D, Pelloux J, Brownlee C, Harper JF (2002). Calcium at the crossroads of signaling. Plant Cell.

[6] Harmon AC, Gribskov M, Gubrium E, Harper JF (2001). The CDPK superfamily of protein kinases. New Phytol.

[7] Ludwig AA, Romeis T, Jones JDG (2004). CDPK-mediated signalling pathways: specificity and cross-talk. J Exp Bot.

[8] Boudsocq M, Willmann MR, McCormack M, Lee H, Shan LB, He P (2010). Differential innate immune signalling via Ca^2+^ sensor protein kinases. Nature.

[9] Cheng SH, Willmann MR, Chen HC, Sheen J (2002). Calcium signaling through protein kinases. The *Arabidopsis* calcium-dependent protein kinase gene family. Plant Physiol.

[10] Ray S, Agarwal P, Arora R, Kapoor S, Tyagi AK (2007). Expression analysis of calcium-dependent protein kinase gene family during reproductive development and abiotic stress conditions in rice (*Oryza sativa* L. *ssp indica*). Mol Genet Genomics.

[11] Li AL, Zhu YF, Tan XM, Wang X, Wei B, Guo HZ (2008). Evolutionary and functional study of the CDPK gene family in wheat (*Triticum aestivum* L.). Plant Mol Biol.

[12] Kong XP, Lv W, Jiang SS, Zhang D, Cai GH, Pan JW (2013). Genome-wide identification and expression analysis of calcium-dependent protein kinase in maize. BMC Genomics.

[13] Zuo R, Hu RB, Chai GH, Xu ML, Qi G, Kong YZ (2013). Genome-wide identification, classification, and expression analysis of CDPK and its closely related gene families in poplar (*Populus trichocarpa*). Mol Biol Rep.

[14] Davletova S, Meszaros T, Miskolczi P, Oberschall A, Torok K, Magyar Z (2001). Auxin and heat shock activation of a novel member of the calmodulin like domain protein kinase gene family in cultured alfalfa cells. J Exp Bot.

[15] Raices M, Ulloa RM, MacIntosh GC, Crespi M, Tellez-Inon MT (2003). StCDPK1 is expressed in potato stolon tips and is induced by high sucrose concentration. J Exp Bot.

[16] Llop-Tous I, Dominguez-Puigjaner E, Vendrell M (2002). Characterization of a strawberry cDNA clone homologous to calcium-dependent protein kinases that is expressed during fruit ripening and affected by low temperature. J Exp Bot.

[17] Chico JM, Raices M, Tellez-Inon MT, Ulloa RM (2002). A calcium-dependent protein kinase is systemically induced upon wounding in tomato plants. Plant Physiol.

[18] Xing T, Wang XJ, Malik K, Miki BL (2001). Ectopic expression of an *Arabidopsis* calmodulin-like domain protein kinase-enhanced NADPH oxidase activity and oxidative burst in tomato protoplasts. Mol Plant Microbe In.

[19] Coca M, Segundo B (2010). *AtCPK*1 calcium-dependent protein kinase mediates pathogen resistance in *Arabidopsis*. Plant J.

[20] Cheng SH, Sheen J, Gerrish C, Bolwell GP (2001). Molecular identification of phenylalanine ammonia-lyase as a substrate of a specific constitutively active *Arabidopsis* CDPK expressed in maize protoplasts. Febs Lett.

[21] Dubiella U, Seybold H, Durian G, Komander E, Lassig R, Witte CP (2013). Calcium-dependent protein kinase/NADPH oxidase activation circuit is required for rapid defense signal propagation. Proc Natl Acad Sci U S A.

[22] Zhu SY, Yu XC, Wang XJ, Zhao R, Li Y, Fan RC (2007). Two calcium-dependent protein kinases, CPK4 and CPK11, regulate abscisic acid signal transduction in *Arabidopsis*. Plant Cell.

[23] Mori IC, Murata Y, Yang Y, Munemasa S, Wang YF, Andreoli S (2006). CPK6 and CPK3 Function in ABA Regulation of Guard Cell S-Type Anion- and Ca^2+^- Permeable Channels and Stomatal Closure. PLoS Biol.

[24] Ma SY, Wu WH (2007). AtCPK23 functions in *Arabidopsis* responses to drought and salt stresses. Plant Mol Biol.

[25] Choi HI, Park HJ, Park JH, Kim S, Im MY, Seo HH (2005). *Arabidopsis* calcium-dependent protein kinase AtCPK32 interacts with ABF4, a transcriptional regulator of abscisic acid-responsive gene expression, and modulates its activity. Plant Physiol.

[26] Zhao R, Sun HL, Mei C, Wang XJ, Yan L, Liu R (2011). The *Arabidopsis* Ca^2+^-dependent protein kinase CPK12 negatively regulates abscisic acid signaling in seed germination and post-germination growth. New Phytol.

[27] Munemasa S, Hossain MA, Nakamura Y, Mori IC, Murata Y (2011). The *Arabidopsis* Calcium-Dependent Protein Kinase, CPK6. Functions as a Positive Regulator of Methyl Jasmonate Signaling in Guard Cells. Plant Physiol.

[28] Luo XJ, Chen ZZ, Gao JP, Gong ZZ (2014). Abscisic acid inhibits root growth in *Arabidopsis* through ethylene biosynthesis. Plant J.

[29] Wang YJ, Liu Y, He P, Chen J, Lamicanra O, Lu J (1995). Evaluation of foliar resistance to *Uncinula necator* in Chinese wild *Vitis* species. Vitis.

[30] Wen YQ, Wang XP, Xiao SY, Wang YJ (2012). Ectopic expression of *VpALDH2B4*, a novel aldehyde dehydrogenase gene from Chinese wild grapevine (*Vitis pseudoreticulata*), enhances resistance to mildew pathogens and salt stress in *Arabidopsis*. Planta.

[31] Chen F, Fasoli M, Tornielli GB, Dal Santo S, Pezzotti M, Zhang LS (2013). The Evolutionary History and Diverse Physiological Roles of the Grapevine Calcium-Dependent Protein Kinase Gene Family. PLoS One.

[32] Dubrovina AS, Kiselev KV, Khristenko VS (2013). Expression of calcium-dependent protein kinase (CDPK) genes under abiotic stress conditions in wild-growing grapevine *Vitis amurensis*. J Plant Physiol.

[33] Boudsocq M, Sheen J (2013). CDPKs in immune and stress signaling. Trends Plant Sci.

[34] Zhang JQ, Guo C, Liu GF, Li ZN, Li XM, Bao MZ (2011). Genetic alteration with variable intron/exon organization amongst five PI-homoeologous genes in *Platanus acerifolia*. Gene.

[35] Vision TJ, Brown DG, Tanksley SD (2000). The origins of genomic duplications in *Arabidopsis*. Science.

[36] Hughes AL (1994). The Evolution of Functionally Novel Proteins after Gene Duplication. P Roy Soc B-Biol Sci.

[37] Jaillon O (2007). The grapevine genome sequence suggests ancestral hexaploidization in major angiosperm phyla. Nature.

[38] Dammann C, Ichida A, Hong BM, Romanowsky SM, Hrabak EM, Harmon AC (2003). Subcellular targeting of nine calcium-dependent protein kinase isoforms from *Arabidopsis*. Plant Physiol.

[39] Latz A, Mehlmer N, Zapf S, Mueller TD, Wurzinger B, Pfister B (2013). Salt Stress Triggers Phosphorylation of the *Arabidopsis* Vacuolar K Channel TPK1 by Calcium-Dependent Protein Kinases (CDPKs). Mol Plant.

[40] Fujita M, Fujita Y, Noutoshi Y, Takahashi F, Narusaka Y, Yamaguchi-Shinozaki K (2006). Crosstalk between abiotic and biotic stress responses: a current view from the points of convergence in the stress signaling networks. Curr Opin Plant Biol.

[41] Zou JJ, Wei FJ, Wang C, Wu JJ, Ratnasekera D, Liu WX (2010). *Arabidopsis* Calcium-Dependent Protein Kinase CPK10 Functions in Abscisic Acid- and Ca^2+^-Mediated Stomatal Regulation in Response to Drought Stress. Plant Physiol.

[42] Xu GX, Guo CC, Shan HY, Kong HZ (2012). Divergence of duplicate genes in exon-intron structure. Proc Natl Acad Sci U S A.

[43] Hamel LP, Sheen J, Seguin A (2014). Ancient signals: comparative genomics of green plant CDPKs. Trends Plant Sci.

[44] Cannon SB, Mitra A, Baumgarten A, Young ND, May G (2004). The roles of segmental and tandem gene duplication in the evolution of large gene families in *Arabidopsis thaliana*. BMC Plant Biol.

[45] Ghiurcuta CG, Moret BME (2014). Evaluating synteny for improved comparative studies. Bioinformatics.

[46] Bohmer M, Romeis T (2007). A chemical-genetic approach to elucidate protein kinase function in planta. Plant Mol Biol.

[47] Lu SX, Hrabak EM (2013). The myristoylated amino-terminus of an *Arabidopsis* calcium-dependent protein kinase mediates plasma membrane localization. Plant Mol Biol.

[48] Gao X, He P (2013). Nuclear dynamics of *Arabidopsis* calcium-dependent protein kinases in effector- triggered immunity. Plant Signal Behav.

[49] Ma Y, Zhao YC, Walker RK, Berkowitz GA (2013). Molecular Steps in the Immune Signaling Pathway Evoked by Plant Elicitor Peptides: Ca^2+^-Dependent Protein Kinases, Nitric Oxide, and Reactive Oxygen Species Are Downstream from the Early Ca^2+^ Signal. Plant Physiol.

[50] Ronzier E, Corratge-Faillie C, Sanchez F, Prado K, Briere C, Leonhardt N (2014). CPK13, a noncanonical Ca^2+^-dependent protein kinase, specifically inhibits KAT2 and KAT1 shaker K^+^ channels and reduces stomatal opening. Plant Physiol.

[51] Gao X, Chen X, Lin W, Chen S, Lu D (2013). Bifurcation of *Arabidopsis* NLR Immune Signaling via Ca^2+^-Dependent Protein Kinases. PLoS Pathol.

[52] Kühn N, Guan L, Dai ZW, Wu BH, Lauvergeat V, Gomès E (2014). Berry ripening: recently heard through the grapevine. J Exp Bot.

[53] Kühn N, Abello C, Godoy F, Delrot S, Patricio AJ (2014). Differential Behavior within a Grapevine Cluster: Decreased Ethylene-Related Gene Expression Dependent on Auxin Transport Is Correlated with Low Abscission of First Developed Berries. PLoS One.

[54] Huang S J, Chang C L, Wang P H, Tsai MC, Hsu PH, Chang F. A type III ACC synthase, ACS7, is involved in root gravitropism in *Arabidopsis thaliana*. J Exp Bot. 2013: ert241. 10.1093/jxb/ert241 PMC3808318 23943848

[55] Ludwig AA, Saitoh H, Felix G, Freymark G, Miersch O, Wasternack C (2005). Ethylene-mediated cross-talk between calcium-dependent protein kinase and MAPK signaling controls stress responses in plants. Proc Natl Acad Sci U S A.

[56] Kamiyoshihara Y, Iwata M, Fukaya T, Tatsuki M, Mori H (2010). Turnover of LeACS2, a wound-inducible 1-aminocyclopropane-1-carboxylic acid synthase in tomato, is regulated by phosphorylation/ dephosphorylation. The Plant J.

[57] Tamura K, Peterson D, Peterson N, Stecher G, Nei M, Kumar S (2011). MEGA5: Molecular Evolutionary Genetics Analysis using Maximum Likelihood, Evolutionary Distance, and Maximum Parsimony Methods. Mol Biol Evol.

[58] Yoo SD, Cho YH, Sheen J (2007). *Arabidopsis* mesophyll protoplasts: a versatile cell system for transient gene expression analysis. Nat Protocols.

