# Supplementary material for: Genome-wide Identification and Expression Analysis of the CDPK Gene Family in Grape, Vitis spp
Source: BMC Plant Biol. 2015 Jun 30;15:164. doi: 10.1186/s12870-015-0552-z (PMC4485369; doi:10.1186/s12870-015-0552-z)
Supplement: Additional file 1: — The syntenic relationships among grape and Arabidopsis CDPK genes. [file 12870_2015_552_MOESM1_ESM.doc]

**Additional file 1. The syntenic relationships among grape and *Arabidopsis*** CDPK genes and within grape CDPK genes.

| **Block ID** | **Block 1 in *Arabidopsis*** | | | |  | **Block 2 in Grape** | | | | |
| --- | --- | --- | --- | --- | --- | --- | --- | --- | --- | --- |
|  | **Chr** | **Start** | **Stop** | **GeneID** |  | **Chr** | **Start** | **Stop** | **Gene ID** | **Gene** |
| 103 | at1 | 6426898 | 6560489 | AT1G18890 |  | chr17 | 7218050 | 5809177 | GSVIVT01008077001 | *VvCDPK14* |
| 44 | at1 | 22882215 | 23044855 | AT1G61950 |  | chr10 | 1118628 | 433319 | GSVIVT01012730001 | *VvCDPK12* |
| 101 | at1 | 27910314 | 28115229 | AT1G74740 |  | chr17 | 8394515 | 5809177 | GSVIVT01008077001 | *VvCDPK14* |
| 121 | at1 | 28524493 | 28783248 | AT1G76040 |  | chr18 | 1777433 | 70305 | GSVIVT01008749001 | *VvCDPK15* |
| 284 | at2 | 7634274 | 7990586 | AT2G17890 |  | chr4 | 20841170 | 18395062 | GSVIVT01018778001 | *VvCDPK3* |
| 296 | at2 | 13399128 | 13450095 | AT2G31500 |  | chr5 | 22089719 | 21889277 | GSVIVT01010743001 | *VvCDPK4* |
| 320 | at2 | 17044963 | 17161136 | AT2G41140 |  | chr8 | 11612540 | 12357537 | GSVIVT01025745001 | *VvCDPK18* |
| 329 | at2 | 17323495 | 17493922 | AT2G41860 |  | chr8 | 22354933 | 21572153 | GSVIVT01033306001 | *VvCDPK11* |
| 339 | at3 | 7079617 | 7192453 | AT3G20410 |  | chr10 | 1213743 | 132655 | GSVIVT01012730001 | *VvCDPK12* |
| 413 | at3 | 7096348 | 7273947 | AT3G20410 |  | chr19 | 6519950 | 7014427 | GSVIVT01037652001 | *VvCDPK17* |
| 478 | at3 | 19130768 | 19419782 | AT3G51850 |  | chr8 | 10796852 | 4597152 | GSVIVT01011167001 | *VvCDPK10* |
| 475 | at3 | 21199488 | 21428253 | AT3G57530 |  | chr8 | 22372711 | 21237910 | GSVIVT01033306001 | *VvCDPK11* |
| 502 | at4 | 1870422 | 2429850 | AT4G04695 |  | chr10 | 256137 | 1176476 | GSVIVT01012730001 | *VvCDPK12* |
| 505 | at4 | 11532971 | 11719828 | AT4G21940 |  | chr10 | 1298924 | 433319 | GSVIVT01012730001 | *VvCDPK12* |
| 578 | at4 | 16796256 | 16826061 | AT4G35310 |  | chr3 | 2875914 | 2962563 | GSVIVT01023866001 | *VvCDPK2* |
| 594 | at4 | 16865488 | 17279201 | AT4G36070 |  | chr4 | 20881776 | 18296443 | GSVIVT01018778001 | *VvCDPK3* |
| 769 | at5 | 4044622 | 4136688 | AT5G12480 |  | chr6 | 2862140 | 1607345 | GSVIVT01025249001 | *VvCDPK5* |
| 739 | at5 | 26397672 | 26555859 | AT5G66210 |  | chr4 | 20807243 | 18530894 | GSVIVT01018778001 | *VvCDPK3* |
