# Supplementary material for: Genome-wide Identification and Expression Analysis of the CDPK Gene Family in Grape, Vitis spp
Source: BMC Plant Biol. 2015 Jun 30;15:164. doi: 10.1186/s12870-015-0552-z (PMC4485369; doi:10.1186/s12870-015-0552-z)
Supplement: Additional file 2: — The CDPKs amino acid sequences used to construct the phylogenetic tree. [file 12870_2015_552_MOESM2_ESM.pdf]

Table S3. The CDPKs amino acid sequences used to construct the phylogenetic tree.

>VvCDPK1
MGNCNGLPSGDTQFQINAGRDGGVRPSSHAHSSNGLNVRPPSPSPPPPPRTAAPVVHRPLA
GNGRVLGRPMEDVRQTYIFGRELGRGQFGVTYLVTHKETKEQFACKSIATRKLVNRDDIE
DVRREVQIMHHLTGHRNIVELKGAYEDRHSVNLVMELCAGGELFDRIIAKGHYSERAAA
ALCRQIVTVVHNCHTMGVMHRDLKPENFLFLSTAEDSPLKATDFGLSVFFKPGDVFKDLV
GSAYYVAPEVLRRSYGAEADIWSAGVILFILLSGVPPFWGENEQSIFDTILRGHIDFSSDPW
PSISNSAKDLVKKMLRADPKERLTAIDVLNHPWMKEDGASDKPIDIAVLTRVKQFRAMNK
LKKVALKVIAENLSEEEIIGLKEMFKSMDTDNSGTITYEELKNGLPKLGTKLSESEVRQLM
EAADVDGNGTIDYIEFISATMHMNRMEREDHLYRAFEYFDKDKSGYITMEELEHALKRY
NMGDEKTIKEIIAEVDTDHDGRINYEEFAAMMRKGNPDLITNRRRK
>VvCDPK2
MGNTCRGSFRGKYFQGYGQPEEQSTSKRNSDRSNSDYSPSSLNSQQLVSQEFAKENPKKE
TRVSILSPTNKDGTMRRGVDNQSYYVLGHKTANIRDLYSLGRKLGQGQFGTTYLCTDMA
TGIEYACKSISKRKLISREDVEDVRREIQIMHHLAGHKNIVTIKGAYEDPLYVHIVMELCAG
GELFDRIIQRGHYSERKAAELTKIIVGVVEACHSLGVMHRDLKPENFLLVNKDDDFSLKAI
DFGLSVFFKPGQVFTDVVGSPYYVAPEVLCKHYGPEADVWTAGVILYILLSGVPPFWAET
QQGIFDAVLKGFIDFESEPWPLISDSAKDLIRKMLCSRPADRLTAHEVLCHPWICENGVAPD
RSLDPAVLSRLKQFSAMNKLKKMALRVIAESLSEEEIAGLREMFKAMDTDSSGAITFDELK
AGLRRYGSTLKESEIRDLMDAADVDNSGTIDYGEFIAATVHLNKLEREEHLVAAFQYFDK
DGSGYITVDELQQACAEHNMTDVFLEDIIKEVDQDNDGRIDYSEFVAMMQKGNAGIGRR
TMRNSLNMSMRDAPGAF
>VvCDPK3
MGACLSATKVRSSNSNTTANAAATKNTRPRGSSKTSNKNQQKKPQEGDRNRSNQQHRNP
QPQKVKDRANGRRGTGIIPCGKRTDFGYAKDFDARYTIGKLLGHGQFGYTYVATDKANG
DRVAVKRIEKNKMILPIAVEDVKREVKILEALTGHENVVQFHNAFEDDSYVYIVMELCEGG
ELLDRILAKKDSRYSEKDAAKVVRQMLKVAAECHLHGLVHRDMKPENFLFKSTKEDSPL
KATDFGLSDFIKPGKKFQDIVGSAYYVAPEVLRRKSGPESDVWSIGVITYILLCGKRPFWD
KTEDGIFKEVLKNKPDFRRKPWPTISNGAKDFVKKLLVKDPRARLTAAQALSHPWVREG
GDASEIPIDISVLSNMREFVKYSHLKQFALRALASTLDDEELADLRDQFDAIDVDKNGSISL
EEMRQALAKDLPWKMKESRVLEILQAIDSNTDGLVDFTEFVAATLHVHQLEEHDSDKWR
QRSQAAFDKFDVDRDGFITPEELKLHTGLRGSIDPLLEEADIDKDGRISLAEFRRLLRTASIS
SRQVPSSSGFRNPRKI
>VvCDPK4
MGGCISMPAKAGQKKKAKAKKIIPESGDDVFRKSVTIRPISVLKEPSGKDIYKTYRLGKEL
GRGEFGVTHQCFDLETGEIFACKTISKSKLTTEIDIQDVRREVEIMKHLPKHPNIVRLKEAY
EDKDNVHLVMELCEGGELFDRIVARGHYTERAAADVTRSIVEILQICHQHGVMHRDLKPE
NFLFADASEASPLKAIDFGLSIFFKPGQRFNEIVGSPYYMAPEVLRRHYGPEVDVWSAGVI
LYILLCGVPPFWAETEEGIAQAIVKSVVDFERDPWPHVSEDAKDLVRSMLDPNPYNRLTVE
EVLAHPWIKNATSIPNVSLGENVRTRIKQFSLMNKFKKRVLRVVADNLPNEQRDGIRQIFH
MMDTDKNGNLSFEELKDGLHKIGHPVADPDVKMLIEAADMDGTGTLNCDEFVTISVHLR

KISSDENLSEAFRAFDKNDSGYIEFEELREALREDNLGPNNEQVIQDIIFDVDLDKDGRISY
DEFKAMMKTGMDWKMSSRQYSRAMLNALSMRIFKDKSMPLQNKSMQLENRSMLLKN
RSMRLQNQVFV
>VvCDPK5
MGNCCASPGSEKENPYLIDYNVLHGSVSRNRGPVLKDPTGRDISLKYELGREMGRGEFG
VTYMCTEKSTNEKYACKSIAKKKLRTAVDIEDVRREVQIMKRLPMHPNIVSLKDTFEDEN
AVHIVMELCEGGELFDRIVSRGHYTERAAAGVMRTIVEVVQICHKHGVMHRDLKPENFLF
ANKKEAAPLKIIDFGLSVDFKHGERFSEIVGSPYYMAPEVLKHNYGPEIDIWSAGVILYILL
CGIPPFWAETEQGVAQAIIRAVVDFKRDPWPKVSDKAKELVKKMLDPDPKKRLTAQEVLD
HPWLQDAKSVPNVSLGESVKARLKQFSMMNKLKKKALQVMAEHLSMEEVAGIKEAFKT
MDINNRGQINLDELRSGLQKLGQPIPDSDLQILMEAADLDGDGTLNYAEFVAVSIHIKKITN
EEHLHKAFAFFDQNQSGYIEIEELQNALADELSTNSEEVINAIMHDVDTDKDGRISYEEFA
AMMKAGTDWRKASRQYSRERFNTLSLKLTRDGKDGQ
>VvCDPK6
MGNCCSQGNTNDGPANDKGETIPEPTTNPETAAPESSAAQNKPAASSTPAASSGASTKPSK
PTPIGPVLGRPMEDVRTLYTIGKELGRGQFGVTHLCTSKATGEQFACKTIAKRKLVNKEDI
EDVRREVQIMHHLTGQPNIVELKGAYEDKQSVHLVMELCAGGELFDRIISKGHYTERGAA
SLLRTIVQIVHTCHSMGVVHRDLKPENFLLLNKDENAPLKATDFGLSVFFKQGEVFRDIVG
SAYYIAPEVLKRRYGPEVDIWSVGVMLYILLCGVPPFWAESEHGIFNAILRGHIDFTSDPWP
TISSGAKDLVRKMLTSDPKQRITAFQVLNHPWIKEDGEAPDTPLDNAVFERFKQFRAMNK
FKKVALRVIAGCLSEEEIMGLKQMFKGMDTDNSGTITLEELKQGLSKQGTKLSEYEVKQL
MEAADADGNGTIDYDEFITATMHLNRMDKEDHLYTAFQYFDKDNSGYITTEELEQALHEF
GMHDGRDIKEILNEVDGDNDGRINYDEFVTMMRKGNPEPNPKKRRDVFV
>VvCDPK7
MGMYTSKDFKNNKKTIIRESLPFSILPSTGERRASGICPFPPEMKKSSAGAPSKPTKPAWVL
PYKTQDLRTLYTIGQKLGQGQFGTTFLCTDKATGHNYACKSIPKRKLFCKEDYDDVWREI
QIMHHLSEHPNVVRIRGTYEDPVFVHLVMELCEGGELFDRIVQRGHYSEREAAKLIKTIVG
VVEGCHSLGVMHRDLKPENFLFDTTAEDAALKATDFGLSVFYKPGETFSDVVGSPYYVAP
EVLCKHYGPEADVWSAGVILYILLSGVPPFWAETETGIFRQILQGKLDFESEPWPCISETAK
ELLRKMLDRNPKKRLTAHEVLSHPWVVDDRMAPDKPLDSAVLSRLKQFSAMNKLKKMA
LRVIAEGLSEEEIGGLRELFKMIDTDNSGTITFDELKDGLKRVGSELMESEIRDLMNAADID
NSGTIDYGEFLAATVHLNKLEREENLVSAFSFFDKDKSGYITIDELQQACKEFGLSEAHLD
DMIKEIDQDNDGQIDYGEFAAMMRKGNGGIGRRTMRNNLNLGDVLGIPDMRLTN
>VvCDPK8
MGNTCVGPNLAANGFLQSVSAAVWRTRPPEDMLPPPNADGSSSGDNAGSDGAKGSDPP
MPVQSTPPETVKITAEVQKAEKSDGNSGKQKKQTHMKRLSSAGLQIDSVLQRNTENLKEI
YSLGRKLGQGQFGTTYLCVEKANGKEFACKSIAKRKLTTREDVEDVRREIQIMHHLAGHP
NVISIVGAFEDAVAVHVVMELCVGGELFDRIIQRGHYTERKAADLARVIVGVVEACHSLG
VMHRDLKPENFLFINQDEDSPLKTIDFGLSMFFRPGEIFTEVVGSPYYVAPEVLRKHYGPE
CDVWSAGVIIYILLSGVPPFWDETEQGIFEQVLKGDLDFVSEPWPSISDSAKDLVRKMLVR
DPKKRLTAHEVLCHPWVQVNGVAPDKPLDSAVLTRLKQFSAMNKLKKIAIRVIAESLSEEE
IAGLKEMFKMIDVDNSGNITLEELKTGLERVGADLKDSEIIRLMQAADIDNSGTIDYGEFV
AAMLHLNKIEKEDHLYAAFSYFDKDGSGYITQDELQQACEQFGLEAIHLEDVIREVDQDN
DGRIDYSEFVAMMQDRDFGKKGYKIT

>VvCDPK9
MGNNCVGSMVPEHGLFESISNSIWWTRASECMTSHSTGEGVSETQSKEQKSPPPVQNKPP
EEVELINPPEVMKITKEETKPTPTPKRPLLMKRLPSAGLEVDLVLKDKTDHLKEHYNLGR
KLGHGQFGTTFLCVEKETGKEYACKSIAKRKLLTRDDIEDVRREIQIMHHLAGHSNIISIKG
AYEDAVAVHLVMELCTGGELFDRIAKRGHYTERKAAQLARTIIGVVEACHSLGVMHRDL
KPENFLFVNEQEESLLKTIDFGLSVFFKPGEIFTDVVGSPYYVAPEVLRKCYGPEADVWSV
GVIIYILLSGVPPFWAESEQEIFQEVLHGDLNFSSDPWPHISESAKDLIRRILVRDPKKRLTA
HEVLCHPWIQVDGVAPDKTLDSAVISRLKQFSAMNKLKKMALRVIAENLSEEEIAGLKEM
FKIIDTDNSGQITFEELKAGLKRFGANLNEAEIYDLMQAADVDNSGTIDYGEFIAATFHLN
KIEREDHLFAAFSYFDKDGSGYITPDELQKACEEFGMEDVHLEEMIQEVDQDNDGRIDYN
EFVAMMQQGNNNDFGKKGLQNGISFGFRQPLPVY
>VvCDPK10
MGNCCRSPAAVAREDVKSANYSGHDHGRKDAGAGKKTITVLNGVSKDGIEEKYMVDRE
LGRGEFGVTYLCIDRDTRELLACKSISKRKLRTAVDVEDVRREVAIMKHLPKNSSIVSLKE
ACEDENAVHLVMELCEGGELFDRIVARGHYTERAAAAVTRTIVEVVQLCHKHGVIHRDLK
PENFLFANKKENSPLKAIDFGLSIFFKPGERFSEIVGSPYYMAPEVLKRNYGPEIDIWSAGVI
LYILLCGVPPFWAESEQGVAQAILRGLIDFKRDPWPNISESAKSLVRQMLEPDPKLRLTAKQ
VLEHSWLQNAKKAPNVPLGDVVKARLKQFSMMNRFKRKALRVIADHLSTEEVEDIKESF
KKMDTDNDGIVSIEELKSGLRKFGSQLAEAEVQMLIETVDTNGKGTLDYGEFVAVSLHLQ
RMANDEHLRKAFSYFDRDGNGYIERDELRDALMEDGADDCTDVANDIFQEVDTDKDGKI
SYDEFAAMMKTGTDWRKASRHYSRGRFNSLSIKLMKDGSLNLGNE
>VvCDPK11
MGNCCVTPVPSEKKKGKKKQNPFSLDYAANQGNGGSKLSVLKDPTGREIELRYELGREL
GRGEFGITHLCTDKSTGDVYACKSISKKKLRTAVDIDDVRREVEIMKHLPKHPNIVTLKDT
YEDDNAVHLVMELCEGGELFDRIVARGHYTERAAAAVTKTIVEVVQMCHKHGVMHRDL
KPENFLFANKKETAPLKAIDFGLSVFFKPGERFTEIVGSPYYMAPEVLKRNYGPEVDVWS
AGVILYILLCGVPPFWAETEQGVAQAIIRSVLDFKRDPWPKVSENAKDLVKKMLDPDPKR
RLSAQEVLDHPWLQNAKKAPNVSLGETVRARLKQFSMMNKLKKRALRVIAEHLSVEEV
AGIKEGFQLMDTGNKGKINMDELRVGLQKLGHQIPEQDLQILMEAGDVDGDGHLDYGEF
VAISVHLRKMGNDDHLLKAFEFFDQNNSGYIEIEELRDALAGELESNSEEVINAIIHDVDTD
KDGRISYDEFAAMMKAGTDWRKASRQYSRERFNNLSLKLIRDGSLEVRP
>VvCDPK12
MGCFSSKERVTERDIKGGRSERGGGGGGGGGRNHQGYQEVVVQHPPPVPAPQTAAPTQT
YHQPPPQPPLKPSHPNTRAIQKPDTILGRPFDDIKHYYTLGKELGRGQFGVTYLCTQNSTG
NTYACKSILKRKLVTKNDKEDIKREIQIMQHLTGQPNIVEFKGAYEDRHSVHLVMELCAG
GELFDRIISQGHYSERAAAAICRAIVNVVHICHFMGVMHRDLKPENFLLSSKDEAAMLKA
TDFGLSVFIEEGKVYRDIVGSAYYVAPEVLRRNYGKEIDIWSAGVILYILLSGVPPFWAETE
KGIFDAILQGEIDFESQPWPAISNGAKDLVRKMLTQDRNKRITSAQVLEHPWIREDGEASD
KPIDSAVLSRMKQFRAMNKLKKLALKVIAENLSEEEIKGLKAMFTNMDTDKSGTITYEEL
KSGLARLGSRLSETEVQQLMEAADVDGNGTIDYIEFITATMHRHRLERDEHLYKAFNYFD
KDNSGFITRDELENAMKEYGMGDEDSIKEIINEVDTDKDGRINYKEFCTMMRSGTQPPVK
LF
>VvCDPK13
MGNTCVGPSISKNGFFQSVSAAMWRSRAPEGSASYTNGETMDEPQATTKEPGSPLPVQN

KPPEQMTIPKEEQPTKPKKPHQIKRVSSAGLRIESVLQTKTGNFKEFFILGRKLGQGQFGTT
FLCVQKATRKEYACKSIAKRKLLTDEDVEDVRREIQIMHHLAGHPNVISIEGAYEDAVAVH
VVMELCKGGELFDRIIQHGHYTERKAAELTRTIVGVVEACHSLGVMHRDLKPENFLLVNE
EEDSLLKTIDFGLSVFFKPGEKFTDVVGSPYYVAPEVLRKRYGPEADVWSAGVILYILLSG
VPPFWAETEQGIFEQVLHGDLDFSSDPWPSISESAKDLVRRMLVRDPRRRLTAHEVLCHPW
VQVDGVAPDKPLDSAVLSRLKQFSAMNKLKKMALIVIAENLSEEEIAGLKEMFKMIDTDN
SGQITFEELKAGLKRVGANLKESEIYDLMHAADVDNNGTIDYGEFIAATLHLNKVEREDH
LFAAFSYFDKDGSGYITPDELQQACEEFGLEDVRLEEMIREVDQDNDGRIDYNEFVAMMQ
KGNPGIGKKGLQTSFSMGFREALKH
>VvCDPK14
MGNCIACVKVDSAEDHRSNHSNSNGHRRKKKTKERRPNPYADDQVRSPAPIRVLKDVIPL
SHRTRIGDKYVLGRELGRGEFGITYLCTDRETRDSLACKSISKKKLRTAVDIEDVRREVSIM
SSLPDHPNIVKLKATYEDSEAVHLVMELCEGGELFDRIVARGHYSERAAAGVARTIAEVVR
MCHENGVIHRDLKPENFLFANKRESSPLKAIDFGLSVFFRPGERFSEIVGSPYYMAPEVLK
RNYGPEVDIWSAGVILYILLCGVPPFWAETEQGVALAILRGVIDFKREPWPQISDNAKSLVR
QMLEQDPRKRLTAQQVLEHSWLQNAKKAPNVPLGDIVRTRLKQFSCMNRFKKKAMRVI
AEHLSVEEVEVIRDMFTLMDTDNDGKVTYEELKAGLRKVGSQLGEPEIKLLMEVADVDG
NGVLDYGEFVAVTIHLQRMENDEHFQRAFMFFDKDGNGFIDLIELQEALADESGETDADV
VNEIMREVDTDKDGRINYDEFVAMMKTGTDWRKASRQYSRERFKSLSLNLMKDGSLHL
EDRITGQSIAV
>VvCDPK15
MGFCFSRPRDIPISSSSSSDGDFTNHHYQPIPISSSKESLDIPPPLSMSVPKPPTSSQIGTVLGR
PLCEITSIYDIGKELGRGQFGITYLCTEKSTGLKYACKSISKRKLKSGKDIEDVKREILILEHL
TGQPNIVEFKGAYEDKQNLHLVMELCSGGELFDRITAKGSYSEREAADICRQIVTVVHVC
HFMGVMHRDLKPENFLMVSREENSPLKATDFGLSVFIEDNEVYKDVVGSAYYVAPEVLR
RSYGKEIDVWSAGVILYILLSGVPPFWGENEKSIFDAVLQGYVDFDSAPWPSISSSAKDLIK
KMLMKDPKRRITASDALNHPWLREDGEASDKPIDSAVLVRMKQFRAMNKLKKLALKVIA
ENLSEEDIKGLKQMFNNMDTDRSGTITFEELKTGLSRLGSKLSELEIKQLMDAVDVDQNG
TLDYTEFITATMQRHRLEKEENLFKAFQFFDKDGSGFITREELKQAMTQYGMGDEATIDE
VIDDVDTDKDGRINYEEFVAMMKKGPQDTELKQR
>VvCDPK16
MVIKTKISYGIRDSVNQHDNVKALLKAIDEQFVTSDKALASTRIMKFSSLRLTDVSGVREH
LMQMRDIVAQLKTLEVEIGGKMELGESALMAMERNDQNQAKKKGKGACVATPSFLNPC
GLKLLKRQLRKGRKKVEKMRKEASKPRAVLPNATPRLREEYQIGRKLGQGQYGITYLCT
HKSSGTHFACKSIPKRKLVCKEDYDDVLREIQIMHHLSEHPHVVQIKATYEDSVFVHLVME
LCAGGELFDRIIQRGHYSERQAAHLIKIIVGVVEACHSLGVMHRDLKPENFLFESTDEDAK
LKATDFGLSVFYKPGEVFFEVVGSPYYVAPEVLRKHYGHEVDVWSAGVILYILLSGVPPF
WAENDTGIFKEILKGKLDFKSDPWPSISESAKDLIKKMLEMDPKKRISAHEVLCHPWIVDD
RVAPDKPLDSAVLSRLKQFSAMNKLKKMALRVIAERLSEEEIGGLKELFKMIDTDNSGTIT
FEELKEGLRKVGSELMESEIKTLMDAADIDNSGTIDYGEFLAATLHLNKMEREENLIAAFS
FFDKDGSGYITIDELQQACREFGLGDAHLDEMIREIDQDNDGRIDYGEFTAMMRKGDGGI
GSRTMRNNLNFNLADAFGINDTT
>VvCDPK17
MGICLSKGKRGSEPAYNGYGSGAVAGGVYDRIQERPAAVQQPPHQLPEKRVPAAQPPMKP

PTVALSPKPVHRPDTILGKSFEDVKLHYTIGKELGRGQFGVTYLCTENSTGLQYACKSISK
RKLVTKNDKEDIRREIQIMQDLSGQPNIVEFKGAYEDRLSVHLVMELCAGGELFDRIIAKG
HYSERAAASICRAIVNVVHICHFMGVMHRDLKPENFLLSSKGENALLKATDFGLSVFIEEG
KVYRDIVGSAYYVAPEVLRRRYGKEIDIWSAGVILYILLSGVPPFWAETEKGIFDAILQGHI
DFETSPWPSISSSAKDLVRKMLTQDPQKRITSAQVLEHPWIKEDGEASDKPIDSAVLSRMK
QFRAMNKLKKLALKVIAENLSEEEIQGLKAMFTNMDTDKSGTITYEELKSGLARLGSKLT
EAEVQQLMEAADVDGNGTIDYIEFITATVNRHKLERDEHLFKAFQYFDKDSSGFITRDELK
AAMKEHGMGDDDTIAEIISEVDTDNDDKINYGEFCSMMRGGTQQGLKVF
>VvCDPK18
MGLCQGKPIENPQTQSQDLIIPGDGELSTNTQTTKTPKFPFYSPSPLPSGFKNSPANSSVSST
PLRIFKRPFPPPSPAKHIRALLARRHGSVKPNEATIPEGNECEVGLDKNFGFSKQFVAHYEM
GEEVGRGHFGYTSSAKAKKGSLKGQDVAVKVIAKSKMTTAIAIEDVRREVKILRALTGHK
NLVQFYEAYEDDDNVYIVMELCRGGELLDRILARGGKYSEEDAKAVMVQILNVTAFCHL
QGVVHRDLKPENFLFISKEENSPLKAIDFGLSDYVKPDERLNDIVGSAYYVAPEVLHRSYG
TEADMWSVGVIAYILLCGSRPFWARTESGIFKAVLKADPSFDEAPWPSLSSDAIDFVKRLL
NKDYRKRLTAAQALSHPWLANYHDVKIPLDMIVYKLVKAYIFSSSLRKSALAALAKTLSI
AQLAYFREQFTLLGPNKSGFVSLQNFKTAVTKNSTDAIKDSRVLEYVSMVGSLQYRKLDF
EEFCAAAISVLQLEGMESWEQHARRGYELFEKDGNRPIMIEELASELGLSPSVPVHVVLQ
DWIRHSDGKLSFLGFVRLLHGPSRAFPKA
>VvCDPK19
MGQETRRLLDEYEVSDVLGRGGFSVVRRGTRKSSSGENPVAIKTLKRCGQTNLPGLPRNR
GSEKRVASMAFPTWKQVSISDALLTNEILVMRKIVEHVSPHPNVINLHDVYEDPSGVHLVL
ELCSGGELFDRIVAQARYSEAGAAAVVKQLAEGLKALHQANIIHRDLKPENCLFLDKSED
ATLKIMDFGLSSVEEFTDPVVGLFGSIDYVSPEVLSQGKISSASDMWSLGVILYILLSGYPPF
IAQSNRQKQQMIIAGDFSFYEKTWKNISSSAKQLISSLLTVDPERRPTAHQLLQHPWVMGD
SAKQDQMDAEIVSRLQSFNARRKFRAAAIASVWSSTVFLRTKKLKTLVGSHDLTQEELEN
LRIHFKEICLKGDNATLSEFEQVLKAMNMSSLIPLAGRIFDLFDNNRDGTVDMREILCGFSS
LRNSQGDDALRLCFQMYDTDRSGCITKEEVASMLRALPDDCLPADITEPGKLDEIFDLMD
ANSDGKVTFEEFKTAMQRDSSLQDVVLSSLRPL
>AtCPK1
MGNTCVGPSRNGFLQSVSAAMWRPRDGDDSASMSNGDIASEAVSGELRSRLSDEVQNKP
PEQVTMPKPGTDVETKDREIRTESKPETLEEISLESKPETKQETKSETKPESKPDPPAKPKKP
KHMKRVSSAGLRTESVLQRKTENFKEFYSLGRKLGQGQFGTTFLCVEKTTGKEFACKSIA
KRKLLTDEDVEDVRREIQIMHHLAGHPNVISIKGAYEDVVAVHLVMECCAGGELFDRIIQR
GHYTERKAAELTRTIVGVVEACHSLGVMHRDLKPENFLFVSKHEDSLLKTIDFGLSMFFK
PDDVFTDVVGSPYYVAPEVLRKRYGPEADVWSAGVIVYILLSGVPPFWAETEQGIFEQVL
HGDLDFSSDPWPSISESAKDLVRKMLVRDPKKRLTAHQVLCHPWVQVDGVAPDKPLDSA
VLSRMKQFSAMNKFKKMALRVIAESLSEEEIAGLKEMFNMIDADKSGQITFEELKAGLKR
VGANLKESEILDLMQAADVDNSGTIDYKEFIAATLHLNKIEREDHLFAAFTYFDKDGSGYI
TPDELQQACEEFGVEDVRIEELMRDVDQDNDGRIDYNEFVAMMQKGSITGGPVKMGLEK
SFSIALKL
>AtCPK2
MGNACVGPNISGNGFLQTVTAAMWRPRIGAEQASSSSHGNGQVSKEAASEPATDQVQNK
PPEPITMPSSKTNPETKLKPDLEIQPEEKKEKVLAEETKQKVVPEESKQEVPPEESKREVVV

QPESAKPETKSESKPETTKPETTSETKPETKAEPQKPKHMRRVSSAGLRTESVLQRKTENF
KEFYSLGRKLGQGQFGTTFLCLEKGTGNEYACKSISKRKLLTDEDVEDVRREIQIMHHLA
GHPNVISIKGAYEDVVAVHLVMELCSGGELFDRIIQRGHYTERKAAELARTIVGVLEACHS
LGVMHRDLKPENFLFVSREEDSLLKTIDFGLSMFFKPDEVFTDVVGSPYYVAPEVLRKRY
GPESDVWSAGVIVYILLSGVPPFWAETEQGIFEQVLHGDLDFSSDPWPSISESAKDLVRKM
LVRDPKRRLTAHQVLCHPWVQIDGVAPDKPLDSAVLSRMKQFSAMNKFKKMALRVIAES
LSEEEIAGLKQMFKMIDADNSGQITFEELKAGLKRVGANLKESEILDLMQAADVDNSGTI
DYKEFIAATLHLNKIEREDHLFAAFSYFDKDESGFITPDELQQACEEFGVEDARIEEMMRD
VDQDKDGRIDYNEFVAMMQKGSIMGGPVKMGLENSISISLKH
>AtCPK3
MGHRHSKSKSSDPPPSSSSSSSGNVVHHVKPAGERRGSSGSGTVGSSGSGTGGSRSTTSTQ
QNGRILGRPMEEVRRTYEFGRELGRGQFGVTYLVTHKETKQQVACKSIPTRRLVHKDDIE
DVRREVQIMHHLSGHRNIVDLKGAYEDRHSVNLIMELCEGGELFDRIISKGLYSERAAAD
LCRQMVMVVHSCHSMGVMHRDLKPENFLFLSKDENSPLKATDFGLSVFFKPGDKFKDLV
GSAYYVAPEVLKRNYGPEADIWSAGVILYILLSGVPPFWGENETGIFDAILQGQLDFSADP
WPALSDGAKDLVRKMLKYDPKDRLTAAEVLNHPWIREDGEASDKPLDNAVLSRMKQFR
AMNKLKKMALKVIAENLSEEEIIGLKEMFKSLDTDNNGIVTLEELRTGLPKLGSKISEAEIR
QLMEAADMDGDGSIDYLEFISATMHMNRIEREDHLYTAFQFFDNDNSGYITMEELELAMK
KYNMGDDKSIKEIIAEVDTDRDGKINYEEFVAMMKKGNPELVPNRRRM
>AtCPK4
MEKPNPRRPSNSVLPYETPRLRDHYLLGKKLGQGQFGTTYLCTEKSSSANYACKSIPKRKL
VCREDYEDVWREIQIMHHLSEHPNVVRIKGTYEDSVFVHIVMEVCEGGELFDRIVSKGCF
SEREAAKLIKTILGVVEACHSLGVMHRDLKPENFLFDSPSDDAKLKATDFGLSVFYKPGQ
YLYDVVGSPYYVAPEVLKKCYGPEIDVWSAGVILYILLSGVPPFWAETESGIFRQILQGKID
FKSDPWPTISEGAKDLIYKMLDRSPKKRISAHEALCHPWIVDEHAAPDKPLDPAVLSRLKQ
FSQMNKIKKMALRVIAERLSEEEIGGLKELFKMIDTDNSGTITFEELKAGLKRVGSELMES
EIKSLMDAADIDNSGTIDYGEFLAATLHINKMEREENLVVAFSYFDKDGSGYITIDELQQA
CTEFGLCDTPLDDMIKEIDLDNDGKIDFSEFTAMMKKGDGVGRSRTMRNNLNFNIAEAFG
VEDTSSTAKSDDSPK
>AtCPK5
MGNSCRGSFKDKLDEGDNNKPEDYSKTSTTNLSSNSDHSPNAADIIAQEFSKDNNSNNNS
KDPALVIPLREPIMRRNPDNQAYYVLGHKTPNIRDIYTLSRKLGQGQFGTTYLCTEIASGV
DYACKSISKRKLISKEDVEDVRREIQIMHHLAGHGSIVTIKGAYEDSLYVHIVMELCAGGE
LFDRIIQRGHYSERKAAELTKIIVGVVEACHSLGVMHRDLKPENFLLVNKDDDFSLKAIDF
GLSVFFKPGQIFTDVVGSPYYVAPEVLLKRYGPEADVWTAGVILYILLSGVPPFWAETQQG
IFDAVLKGYIDFESDPWPVISDSAKDLIRRMLSSKPAERLTAHEVLRHPWICENGVAPDRAL
DPAVLSRLKQFSAMNKLKKMALKVIAESLSEEEIAGLREMFQAMDTDNSGAITFDELKAG
LRKYGSTLKDTEIHDLMDAADVDNSGTIDYSEFIAATIHLNKLEREEHLVAAFQYFDKDGS
GFITIDELQQACVEHGMADVFLEDIIKEVDQNNDGKIDYGEFVEMMQKGNAGVGRRTMR
NSLNISMRDA
>AtCPK6
MGNSCRGSFKDKIYEGNHSRPEENSKSTTTTVSSVHSPTTDQDFSKQNTNPALVIPVKEPI
MRRNVDNQSYYVLGHKTPNIRDLYTLSRKLGQGQFGTTYLCTDIATGVDYACKSISKRKL
ISKEDVEDVRREIQIMHHLAGHKNIVTIKGAYEDPLYVHIVMELCAGGELFDRIIHRGHYSE

RKAAELTKIIVGVVEACHSLGVMHRDLKPENFLLVNKDDDFSLKAIDFGLSVFFKPGQIFK
DVVGSPYYVAPEVLLKHYGPEADVWTAGVILYILLSGVPPFWAETQQGIFDAVLKGYIDFD
TDPWPVISDSAKDLIRKMLCSSPSERLTAHEVLRHPWICENGVAPDRALDPAVLSRLKQFS
AMNKLKKMALKVIAESLSEEEIAGLRAMFEAMDTDNSGAITFDELKAGLRRYGSTLKDT
EIRDLMEAADVDNSGTIDYSEFIAATIHLNKLEREEHLVSAFQYFDKDGSGYITIDELQQSC
IEHGMTDVFLEDIIKEVDQDNDGRIDYEEFVAMMQKGNAGVGRRTMKNSLNISMRDV
>AtCPK7
MGNCCGNPSSATNQSKQGKPKNKNNPFYSNEYATTDRSGAGFKLSVLKDPTGHDISLQY
DLGREVGRGEFGITYLCTDKETGEKYACKSISKKKLRTAVDIEDVRREVEIMKHMPKHPN
VVSLKDSFEDDDAVHIVMELCEGGELFDRIVARGHYTERAAAAVMKTIVEVVQICHKQG
VMHRDLKPENFLFANKKETSALKAIDFGLSVFFKPGEQFNEIVGSPYYMAPEVLRRNYGP
EIDVWSAGVILYILLCGVPPFWAETEQGVAQAIIRSVIDFKRDPWPRVSDSAKDLVRKMLEP
DPKKRLTAAQVLEHTWILNAKKAPNVSLGETVKARLKQFSVMNKLKKRALRVIAEHLSV
EEAAGIKEAFEMMDVNKRGKINLEELKYGLQKAGQQIADTDLQILMEATDVDGDGTLNY
SEFVAVSVHLKKMANDEHLHKAFNFFDQNQSGYIEIDELREALNDELDNTSSEEVIAAIMQ
DVDTDKDGRISYEEFVAMMKAGTDWRKASRQYSRERFNSLSLKLMRDGSLQLEGET
>AtCPK8
MGNCCASPGSETGSKKGKPKIKSNPFYSEAYTTNGSGTGFKLSVLKDPTGHDISLMYDLG
REVGRGEFGITYLCTDIKTGEKYACKSISKKKLRTAVDIEDVRREVEIMKHMPRHPNIVSLK
DAFEDDDAVHIVMELCEGGELFDRIVARGHYTERAAAAVMKTILEVVQICHKHGVMHRD
LKPENFLFANKKETSALKAIDFGLSVFFKPGEGFNEIVGSPYYMAPEVLRRNYGPEVDIWS
AGVILYILLCGVPPFWAETEQGVAQAIIRSVIDFKRDPWPRVSETAKDLVRKMLEPDPKKRL
SAAQVLEHSWIQNAKKAPNVSLGETVKARLKQFSVMNKLKKRALRVIAEHLSVEEVAGI
KEAFEMMDSKKTGKINLEELKFGLHKLGQQQIPDTDLQILMEAADVDGDGTLNYGEFVA
VSVHLKKMANDEHLHKAFSFFDQNQSDYIEIEELREALNDEVDTNSEEVVAAIMQDVDT
DKDGRISYEEFAAMMKAGTDWRKASRQYSRERFNSLSLKLMREGSLQLEGEN
>AtCPK9
MGNCFAKNHGLMKPQQNGNTTRSVEVGVTNQDPPSYTPQARTTQQPEKPGSVNSQPPPW
RAAAAAPGLSPKTTTKSNSILENAFEDVKLFYTLGKELGRGQFGVTYLCTENSTGKKYAC
KSISKKKLVTKADKDDMRREIQIMQHLSGQPNIVEFKGAYEDEKAVNLVMELCAGGELFD
RIIAKGHYTERAAASVCRQIVNVVKICHFMGVLHRDLKPENFLLSSKDEKALIKATDFGLS
VFIEEGKVYRDIVGSAYYVAPEVLRRRYGKEVDIWSAGIILYILLSGVPPFWAETEKGIFDAI
LEGHIDFESQPWPSISSSAKDLVRRMLTADPKRRISAADVLQHPWLREGGEASDKPIDSAV
LSRMKQFRAMNKLKKLALKVIAENIDTEEIQGLKAMFANIDTDNSGTITYEELKEGLAKL
GSKLTEAEVKQLMDAADVDGNGSIDYIEFITATMHRHRLESNENLYKAFQHFDKDSSGYI
TIDELESALKEYGMGDDATIKEVLSDVDSDNDGRINYEEFCAMMRSGNPQQQQPRLF
>AtCPK10
MGNCNACVRPDSKESKPSSKPKKPNRDRKLNPFAGDFTRSPAPIRVLKDVIPMSNQTQISD
KYILGRELGRGEFGITYLCTDRETHEALACKSISKRKLRTAVDIEDVRREVAIMSTLPEHPN
VVKLKASYEDNENVHLVMELCEGGELFDRIVARGHYTERAAAAVARTIAEVVMMCHSNG
VMHRDLKPENFLFANKKENSPLKAIDFGLSVFFKPGDKFTEIVGSPYYMAPEVLKRDYGP
GVDVWSAGVIIYILLCGVPPFWAETEQGVALAILRGVLDFKRDPWPQISESAKSLVKQML
DPDPTKRLTAQQVLAHPWIQNAKKAPNVPLGDIVRSRLKQFSMMNRFKKKVLRVIAEHLS
IQEVEVIKNMFSLMDDDKDGKITYPELKAGLQKVGSQLGEPEIKMLMEVADVDGNGFLD

YGEFVAVIIHLQKIENDELFKLAFMFFDKDGSTYIELDELREALADELGEPDASVLSDIMRE
VDTDKDGRINYDEFVTMMKAGTDWRKASRQYSRERFKSLSINLMKDGSLHLHDALTGQ
TVPV
>AtCPK11
METKPNPRRPSNTVLPYQTPRLRDHYLLGKKLGQGQFGTTYLCTEKSTSANYACKSIPKR
KLVCREDYEDVWREIQIMHHLSEHPNVVRIKGTYEDSVFVHIVMEVCEGGELFDRIVSKG
HFSEREAVKLIKTILGVVEACHSLGVMHRDLKPENFLFDSPKDDAKLKATDFGLSVFYKP
GQYLYDVVGSPYYVAPEVLKKCYGPEIDVWSAGVILYILLSGVPPFWAETESGIFRQILQG
KLDFKSDPWPTISEAAKDLIYKMLERSPKKRISAHEALCHPWIVDEQAAPDKPLDPAVLSR
LKQFSQMNKIKKMALRVIAERLSEEEIGGLKELFKMIDTDNSGTITFEELKAGLKRVGSEL
MESEIKSLMDAADIDNSGTIDYGEFLAATLHMNKMEREENLVAAFSYFDKDGSGYITIDEL
QSACTEFGLCDTPLDDMIKEIDLDNDGKIDFSEFTAMMRKGDGVGRSRTMMKNLNFNIA
DAFGVDGEKSDD
>AtCPK12
MANKPRTRWVLPYKTKNVEDNYFLGQVLGQGQFGTTFLCTHKQTGQKLACKSIPKRKLL
CQEDYDDVLREIQIMHHLSEYPNVVRIESAYEDTKNVHLVMELCEGGELFDRIVKRGHYS
EREAAKLIKTIVGVVEACHSLGVVHRDLKPENFLFSSSDEDASLKSTDFGLSVFCTPGEAF
SELVGSAYYVAPEVLHKHYGPECDVWSAGVILYILLCGFPPFWAESEIGIFRKILQGKLEFEI
NPWPSISESAKDLIKKMLESNPKKRLTAHQVLCHPWIVDDKVAPDKPLDCAVVSRLKKFS
AMNKLKKMALRVIAERLSEEEIGGLKELFKMIDTDKSGTITFEELKDSMRRVGSELMESEI
QELLRAADVDESGTIDYGEFLAATIHLNKLEREENLVAAFSFFDKDASGYITIEELQQAWK
EFGINDSNLDEMIKDIDQDNDGQIDYGEFVAMMRKGNGTGGGIGRRTMRNSLNFGTTLPD
ESMNV
>AtCPK13
MGNCCRSPAAVAREDVKSNYSGHDHARKDAAGGKKSAPIRVLSDVPKENIEDRYLLDRE
LGRGEFGVTYLCIERSSRDLLACKSISKRKLRTAVDIEDVKREVAIMKHLPKSSSIVTLKEA
CEDDNAVHLVMELCEGGELFDRIVARGHYTERAAAGVTKTIVEVVQLCHKHGVIHRDLK
PENFLFANKKENSPLKAIDFGLSIFFKPGEKFSEIVGSPYYMAPEVLKRNYGPEIDIWSAGVI
LYILLCGVPPFWAESEQGVAQAILRGVIDFKREPWPNISETAKNLVRQMLEPDPKRRLTAK
QVLEHPWIQNAKKAPNVPLGDVVKSRLKQFSVMNRFKRKALRVIAEFLSTEEVEDIKVMF
NKMDTDNDGIVSIEELKAGLRDFSTQLAESEVQMLIEAVDTKGKGTLDYGEFVAVSLHLQ
KVANDEHLRKAFSYFDKDGNGYILPQELCDALKEDGGDDCVDVANDIFQEVDTDKDGRI
SYEEFAAMMKTGTDWRKASRHYSRGRFNSLSIKLMKDGSLNLGNE
>AtCPK14
MGNCCGTAGSLIQDKQKKGFKLPNPFSNEYGNHHDGLKLIVLKEPTGHEIKQKYKLGREL
GRGEFGVTYLCTEIETGEIFACKSILKKKLKTSIDIEDVKREVEIMRQMPEHPNIVTLKETYE
DDKAVHLVMELCEGGELFDRIVARGHYTERAAASVIKTIIEVVQMCHKHGVMHRDLKPE
NFLFANKKETASLKAIDFGLSVFFKPGERFNEIVGSPYYMAPEVLRRSYGQEIDIWSAGVIL
YILLCGVPPFWAETEHGVAKAILKSVIDFKRDPWPKVSDNAKDLIKKMLHPDPRRRLTAQ
QVLDHPWIQNGKNASNVSLGETVRARLKQFSVMNKLKKRALRVIAEHLSVEETSCIKERF
QVMDTSNRGKITITELGIGLQKLGIVVPQDDIQILMDAGDVDKDGYLDVNEFVAISVHIRK
LGNDEHLKKAFTFFDKNKSGYIEIEELRDALADDVDTTSEEVVEAIILDVDTNKDGKISYD
EFATMMKTGTDWRKASRQYSRDLFKCLSLKLMQDGSLQSNGDTK
>AtCPK15

MGCFSSKHRNTESDIINGSVQSSIPTNQPENHVSRDVLKPQKPPSPQIPTTTQSNHHHQQES
KPVNQQIEKKHVLTQPLKPIVFRETETILGKPFEEIRKLYTLGKELGRGQFGITYTCKENST
GNTYACKSILKRKLTRKQDIDDVKREIQIMQYLSGQENIVEIKGAYEDRQSIHLVMELCGG
SELFDRIIAQGHYSEKAAAGVIRSVLNVVQICHFMGVIHRDLKPENFLLASTDENAMLKAT
DFGLSVFIEEGKVYRDIVGSAYYVAPEVLRRSYGKEIDIWSAGIILYILLCGVPPFWSETEKG
IFNEIIKGEIDFDSQPWPSISESAKDLVRKLLTKDPKQRISAAQALEHPWIRGGEAPDKPIDS
AVLSRMKQFRAMNKLKKLALKVIAESLSEEEIKGLKTMFANMDTDKSGTITYEELKNGLA
KLGSKLTEAEVKQLMEAADVDGNGTIDYIEFISATMHRYRFDRDEHVFKAFQYFDKDNSG
FITMDELESAMKEYGMGDEASIKEVIAEVDTDNDGRINYEEFCAMMRSGITLPQQGKILP
VQ
>AtCPK16
MGLCFSSAAKSSGHNRSSRNPHPHPPLTVVKSRPPRSPCSFMAVTIQKDHRTQPRRNATAK
KTPTRHTPPHGKVREKVISNNGRRHGETIPYGKRVDFGYAKDFDHRYTIGKLLGHGQFGY
TYVATDKKTGDRVAVKKIDKAKMTIPIAVEDVKREVKILQALTGHENVVRFYNAFEDKNS
VYIVMELCEGGELLDRILARKDSRYSERDAAVVVRQMLKVAAECHLRGLVHRDMKPENF
LFKSTEEDSPLKATDFGLSDFIKPGKKFHDIVGSAYYVAPEVLKRRSGPESDVWSIGVISYIL
LCGRRPFWDKTEDGIFKEVLKNKPDFRRKPWPTISNSAKDFVKKLLVKDPRARLTAAQAL
SHPWVREGGDASEIPIDISVLNNMRQFVKFSRLKQFALRALATTLDEEELADLRDQFDAID
VDKNGVISLEEMRQALAKDHPWKLKDARVAEILQAIDSNTDGFVDFGEFVAAALHVNQL
EEHDSEKWQQRSRAAFEKFDIDGDGFITAEELRMHTGLKGSIEPLLEEADIDNDGKISLQE
FRRLLRTASIKSRNVRSPPGYLISRKV
>AtCPK17
MGNCCSHGRDSADNGDALENGASASNAANSTGPTAEASVPQSKHAPPSPPPATKQGPIGP
VLGRPMEDVKASYSLGKELGRGQFGVTHLCTQKATGHQFACKTIAKRKLVNKEDIEDVR
REVQIMHHLTGQPNIVELKGAYEDKHSVHLVMELCAGGELFDRIIAKGHYSERAAASLLR
TIVQIVHTCHSMGVIHRDLKPENFLLLNKDENSPLKATDFGLSVFYKPGEVFKDIVGSAYYI
APEVLKRKYGPEADIWSIGVMLYILLCGVPPFWAESENGIFNAILRGHVDFSSDPWPSISPQ
AKDLVKKMLNSDPKQRLTAAQVLNHPWIKEDGEAPDVPLDNAVMSRLKQFKAMNNFKK
VALRVIAGCLSEEEIMGLKEMFKGMDTDSSGTITLEELRQGLAKQGTRLSEYEVQQLMEA
ADADGNGTIDYGEFIAATMHINRLDREEHLYSAFQHFDKDNSGYITMEELEQALREFGMN
DGRDIKEIISEVDGDNDGRINYDEFVAMMRKGNPDPIPKKRRELSFK
>AtCPK18
MGLCFSSPKATRRGTGSRNPNPDSPTQGKASEKVSNKNKKNTKKIQLRHQGGIPYGKRID
FGYAKDFDNRYTIGKLLGHGQFGFTYVATDNNNGNRVAVKRIDKAKMTQPIEVEDVKREV
KILQALGGHENVVGFHNAFEDKTYIYIVMELCDGGELLDRILAKKDSRYTEKDAAVVVR
QMLKVAAECHLRGLVHRDMKPENFLFKSTEEGSSLKATDFGLSDFIKPGVKFQDIVGSAY
YVAPEVLKRRSGPESDVWSIGVITYILLCGRRPFWDKTQDGIFNEVMRKKPDFREVPWPTI
SNGAKDFVKKLLVKEPRARLTAAQALSHSWVKEGGEASEVPIDISVLNNMRQFVKFSRLK
QIALRALAKTINEDELDDLRDQFDAIDIDKNGSISLEEMRQALAKDVPWKLKDARVAEILQ
ANDSNTDGLVDFTEFVVAALHVNQLEEHDSEKWQQRSRAAFDKFDIDGDGFITPEELRLQ
TGLKGSIEPLLEEADVDEDGRISINEFRRLLRSASLKSKNVKSPPGTEHIICHNLLDGICIEDT
EERTSAVRFEYVSQVL
>AtCPK19
MGCLCINLKKKVKKPTPDISGEQNTEVKSREITPKEQPRQRQPAPRAKFQIVVQPHKLPLP

LPQPQEKQKLINHQKQSTLQQPEPILGRPFEDIKEKYSLGRELGRGQFGITYICTEISSGKNF
ACKSILKRKLIRTKDREDVRREIQIMHYLSGQPNIVEIKGAYEDRQSVHLVMELCEGGELF
DKITKRGHYSEKAAAEIIRSVVKVVQICHFMGVIHRDLKPENFLLSSKDEASSMLKATDFG
VSVFIEEGKVYEDIVGSAYYVAPEVLKRNYGKAIDIWSAGVILYILLCGNPPFWAETDKGIF
EEILRGEIDFESEPWPSISESAKDLVRNMLKYDPKKRFTAAQVLEHPWIREGGEASDKPIDS
AVLSRMKQLRAMNKLKKLAFKFIAQNLKEEELKGLKTMFANMDTDKSGTITYDELKSGL
EKLGSRLTETEVKQLLEDADVDGNGTIDYIEFISATMNRFRVEREDNLFKAFQHFDKDNSG
FISRQELETAMKEYNMGDDIMIKEIISEVDADNDGSINYQEFCNMMKSCSQSHQSKLVQP
N
>AtCPK20
MGNTCVGPNLNPNGFLQSVSAAVWRNQKPDDSIKSSKDESSRKKNDKSVNGDDSNGHVS
STVDPAPSTLPTPSTPPPPVKMANEEPPPKPITENKEDPNSKPQKKEAHMKRMASAGLQID
SVLGRKTENLKDIYSVGRKLGQGQFGTTFLCVDKKTGKEFACKTIAKRKLTTPEDVEDVR
REIQIMHHLSGHPNVIQIVGAYEDAVAVHVVMEICAGGELFDRIIQRGHYTEKKAAELARII
VGVIEACHSLGVMHRDLKPENFLFVSGDEEAALKTIDFGLSVFFKPGETFTDVVGSPYYV
APEVLRKHYSHECDVWSAGVIIYILLSGVPPFWDETEQGIFEQVLKGDLDFISEPWPSVSES
AKDLVRRMLIRDPKKRMTTHEVLCHPWARVDGVALDKPLDSAVLSRLQQFSAMNKLKKI
AIKVIAESLSEEEIAGLKEMFKMIDTDNSGHITLEELKKGLDRVGADLKDSEILGLMQAAD
IDNSGTIDYGEFIAAMVHLNKIEKEDHLFTAFSYFDQDGSGYITRDELQQACKQFGLADVH
LDDILREVDKDNDGRIDYSEFVDMMQDTGFGKMGLKVS
>AtCPK21
MGCFSSKHRKTQNDGGEKSIPINPVQTHVVPEHRKPQTPTPKPMTQPIHQQISTPSSNPVSV
RDPDTILGKPFEDIRKFYSLGKELGRGQFGITYMCKEIGTGNTYACKSILKRKLISKQDKED
VKREIQIMQYLSGQPNIVEIKGAYEDRQSIHLVMELCAGGELFDRIIAQGHYSERAAAGIIR
SIVNVVQICHFMGVVHRDLKPENFLLSSKEENAMLKATDFGLSVFIEEGKVYRDIVGSAY
YVAPEVLRRSYGKEIDIWSAGVILYILLSGVPPFWAENEKGIFDEVIKGEIDFVSEPWPSISES
AKDLVRKMLTKDPKRRITAAQVLEHPWIKGGEAPDKPIDSAVLSRMKQFRAMNKLKKLA
LKVIAESLSEEEIKGLKTMFANIDTDKSGTITYEELKTGLTRLGSRLSETEVKQLMEAADV
DGNGTIDYYEFISATMHRYKLDRDEHVYKAFQHFDKDNSGHITRDELESAMKEYGMGDE
ASIKEVISEVDTDNDGRINFEEFCAMMRSGSTQPQGKLLPFH
>AtCPK22
MGNCCGSKPLTASDIVSDQKQETILGKPLEDIKKHYSFGDELGKGKSYACKSIPKRTLSSEE
EKEAVKTEIQIMDHVSGQPNIVQIKGSYEDNNSIHIVMELCGGGELFDKIDALVKSHSYYS
EKDAAGIFRSIVNAVKICHSLDVVHRDLKPENFLFSSKDENAMLKAIDFGCSVYIKEGKTF
ERVVGSKYYIAPEVLEGSYGKEIDIWSAGVILYILLSGVPPFQTGIESIIVSTLCIVDAEIKEC
RLDFESQPWPLISFKAKHLIGKMLTKKPKERISAADVLEHPWMKSEAPDKPIDNVVLSRM
KQFRAMNKLKKLALKVIAEGLSEEEIKGLKTMFENMDMDKSGSITYEELKMGLNRHGSK
LSETEVKQLMEAVSADVDGNGTIDYIEFISATMHRHRLERDEHLYKAFQYFDKDGSGHIT
KEEVEIAMKEHGMGDEANAKDLISEFDKNNDGKIDYEEFCTMMRNGILQPQGKLLKRLY
MNLEELKTGLTRLGSRLSETEIDKAFQHFDKDNSGHITRDELESAMKEYGMGDEASIKEVI
SEVDTDNVSCTLQHIANISNIKQVLETL
>AtCPK23
MGCFSSKHRKTQNDGGGERSIPIIPVQTHIVDQVPDHRKPQIPSPSIPISVRDPETILGKPFED
IRKFYSLGRELGRGGLGITYMCKEIGTGNIYACKSILKRKLISELGREDVKTEIQIMQHLSG

QPNVVEIKGSYEDRHSVHLVMELCAGGELFDRIIAQGHYSERAAAGTIKSIVDVVQICHLN
GVIHRDLKPENFLFSSKEENAMLKVTDFGLSAFIEEGKIYKDVVGSPYYVAPEVLRQSYGK
EIDIWSAGVILYILLCGVPPFWADNEEGVFVEILKCKIDFVREPWPSISDSAKDLVEKMLTE
DPKRRITAAQVLEHPWIKGGEAPEKPIDSTVLSRMKQFRAMNKLKKLALKVSAVSLSEEEI
KGLKTLFANMDTNRSGTITYEQLQTGLSRLRSRLSETEVQQLVEASDVDGNGTIDYYEFIS
ATMHRYKLHHDEHVHKAFQHLDKDKNGHITRDELESAMKEYGMGDEASIKEVISEVDT
DNDGKINFEEFRAMMRCGTTQPKGKQYPFH
>AtCPK24
MGSCVSSPLKGSPFGKRPVRRRHSSNSRTSSVPRFDSSTNLSRRLIFQPPSRVLPEPIGDGIH
LKYDLGKELGRGEFGVTHECIEISTRERFACKRISKEKLRTEIDVEDVRREVEIMRCLPKHP
NIVSFKEAFEDKDAVYLVMEICEGGELFDRIVSRGHYTERAAASVAKTILEVVKVCHEHG
VIHRDLKPENFLFSNGTETAQLKAIDFGLSIFFKPAQRFNEIVGSPYYMAPEVLRRNYGPEI
DVWSAGVILYILLCGVPPFWAETEEGIAHAIVRGNIDFERDPWPKVSHEAKELVKNMLDA
NPYSRLTVQEVLEHPWIRNAERAPNVNLGDNVRTKIQQFLLMNRFKKKVLRIVADNLPNE
EIAAIVQMFQTMDTDKNGHLTFEELRDGLKKIGQVVPDGDVKMLMDAADTDGNGMLSC
DEFVTLSIHLKRMGCDEHLQEAFKYFDKNGNGFIELDELKVALCDDKLGHANGNDQWIK
DIFFDVDLNKDGRISFDEFKAMMKSGTDWKMASRQYSRALLNALSIKMFKEDFGDNGPK
SHSMEFPIARKRAKLLDAPKNKSMELQISKTYKPSGLRN
>AtCPK25
MGNVCVHMVNNCVDTKSNSWVRPTDLIMDHPLKPQLQDKPPQPMLMNKDDDKTKLND
THGDPKLLEGKEKPAQKQTSQGQGGRKCSDEEYKKRAIACANSKRKAHNVRRLMSAGL
QAESVLKTKTGHLKEYYNLGSKLGHGQFGTTFVCVEKGTGEEYACKSIPKRKLENEEDV
EDVRREIEIMKHLLGQPNVISIKGAYEDSVAVHMVMELCRGGELFDRIVERGHYSERKAA
HLAKVILGVVQTCHSLGVMHRDLKPENFLFVNDDEDSPLKAIDFGLSMFLKPGENFTDVV
GSPYYIAPEVLNKNYGPEADIWSAGVMIYVLLSGSAPFWGETEEEIFNEVLEGELDLTSDP
WPQVSESAKDLIRKMLERNPIQRLTAQQVLCHPWIRDEGNAPDTPLDTTVLSRLKKFSATD
KLKKMALRVIAERLSEEEIHELRETFKTIDSGKSGRVTYKELKNGLERFNTNLDNSDINSL
MQIPTDVHLEDTVDYNEFIEAIVRLRQIQEEEANDRLESSTKV
>AtCPK26
MHRDLKPENFLLVNKDDDFSLKAIDFGLSVFFKPGQIFEDVVGSPYYVAPEVLLKHYGPE
ADVWTAGVILYILVSGVPPFWAETQQGIFDAVLKGHIDFDSDPWPLISDSAKNLIRGMLCS
RPSERLTAHQVLRHPWICENGVAPDRALDPAVLSRLKQFSAMNKLKQMALRVIAESLSEEE
IAGLKEMFKAMDTDNSGAITFDELKAGLRRYGSTLKDTEIRDLMEAADIDKSGTIDYGEFI
AATIHLNKLEREEHLLSAFRYFDKDGSGYITIDELQHACAEQGMSDVFLEDVIKEVDQDN
DGRIDYGEFVAMMQKGIVGRTMRKSINMSIRNNAVSQ
>AtCPK27
MGCFSSKELQQSKRTILEKPLVDITKIYILGEELGRGNFGLTRKCVEKSTGKTFACKTILKT
KLKDEECEEDVKREIRIMKQLSGEPNIVEFKNAYEDKDSVHIVMEYCGGGELYDKILALY
DVGKSYSEKEAAGIIRSIVNVVKNCHYMGVMHRDLKPENFLLTSNDDNATVKVIDFGCSV
FIEEGKVYQDLAGSDYYIAPEVLQGNYGKEADIWSAGIILYILLCGKSPFVKEPEGQMFNEI
KSLEIDYSEEPWPLRDSRAIHLVKRMLDRNPKERISAAEVLGHPWMKEGEASDKPIDGVV
LSRLKRFRDANKFKKVVLKFIAANLSEEEIKGLKTLFTNIDTDKSGNITLEELKTGLTRLGS
NLSKTEVEQLMEAADMDGNGTIDIDEFISATMHRYKLDRDEHVYKAFQHFDKDNDGHIT
KEELEMAMKEDGAGDEGSIKQIIADADTDNDGKINFEEFRTMMRTESSLQPEGELLPIIN

>AtCPK28
MGVCFSAIRVTGASSSRRSSQTKSKAAPTPIDTKASTKRRTGSIPCGKRTDFGYSKDFHDH
YTIGKLLGHGQFGYTYVAIHRPNGDRVAVKRLDKSKMVLPIAVEDVKREVQILIALSGHEN
VVQFHNAFEDDDYVYIVMELCEGGELLDRILSKKGNRYSEKDAAVVVRQMLKVAGECH
LHGLVHRDMKPENFLFKSAQLDSPLKATDFGLSDFIKPGKRFHDIVGSAYYVAPEVLKRRS
GPESDVWSIGVITYILLCGRRPFWDRTEDGIFKEVLRNKPDFSRKPWATISDSAKDFVKKLL
VKDPRARLTAAQALSHAWVREGGNATDIPVDISVLNNLRQFVRYSRLKQFALRALASTLD
EAEISDLRDQFDAIDVDKNGVISLEEMRQALAKDLPWKLKDSRVAEILEAIDSNTDGLVDF
TEFVAAALHVHQLEEHDSEKWQLRSRAAFEKFDLDKDGYITPEELRMHTGLRGSIDPLLD
EADIDRDGKISLHEFRRLLRTASISSQRAPSPAGHRNLR
>AtCPK29
MFECILAKTLVLYFEIRTSNHLIWLIFVGKVYRDIVGSAYYVAPEVLHRNYGKEIDVWSAG
VMLYILLSGVPPFWGETEKTIFEAILEGKLDLETSPWPTISESAKDLIRKMLIRDPKKRITAA
EALEHPWMTDTKISDKPINSAVLVRMKQFRAMNKLKKLALKVIAENLSEEEIKGLKQTFK
NMDTDESGTITFDELRNGLHRLGSKLTESEIKQLMEAADVDKSGTIDYIEFVTATMHRHRL
EKEENLIEAFKYFDKDRSGFITRDELKHSMTEYGMGDDATIDEVINDVDTDNDGRINYEEF
VAMMRKGTTDSDPKLIR
>AtCPK30
MGNCIACVKFDPDNSKPNQKKKPPRGRQRNPYDDPDGLRTHAPLRVIPMSHQSQISDKYI
LGRELGRGEFGITYLCTDRETREALACKSISKRKLRTAVDVEDVRREVTIMSTLPEHPNVV
KLKATYEDNENVHLVMELCEGGELFDRIVARGHYTERAAATVARTIAEVVRMCHVNGVM
HRDLKPENFLFANKKENSALKAIDFGLSVLFKPGERFTEIVGSPYYMAPEVLKRNYGPEV
DVWSAGVILYILLCGVPPFWAETEQGVALAILRGVLDFKRDPWSQISESAKSLVKQMLEPD
STKRLTAQQVLDHPWIQNAKKAPNVPLGDIVRSRLKQFSMMNRLKKKALRVIAEHLSIQE
VEVIRNMFTLMDDDNDGKISYLELRAGLRKVGSQLGEPEIKLLMEVADVNGNGCLDYGE
FVAVIIHLQKMENDEHFRQAFMFFDKDGSGYIESEELREALTDELGEPDNSVIIDIMREVDT
DKDGKINYDEFVVMMKAGTDWRKASRQYSRERFKSLSLNLMKDGSMHLHDALTGQSIA
V
>AtCPK31
MGCYSSKNLKQSKRTILEKPFVDIGKVYILGDELGQGQFGITRKCVEKTSGKTYACKTILK
TNLKSREDEEAVKREIRIMKHLSGEPNIVEFKKAYEDRDSVHIVMEYCGGGELFKKIEALS
KDGKSYSEKEAVEIIRPIVNVVKNCHYMGVMLRDLKPENFLLSSTDKNATVKAIDFGCSV
FIEEGEVHRKFAGSAYYIAPEVLQGKYGKEADIWSAGIILYILLCGKPPFVTEPEAQMFSEIK
SAKIDVDSESWKFIDVKAKHLVNRMLNRNPKERISAAEVLGHPWMKDGEASDKPIDGVV
LSRLKQFRDMNKLKKVALKVIAANLSEEEIKGLKTLFTNIDTDKSGTITLEELKTGLTRLGS
NLSKTEVEQLMEAADVDGNGTIDIDEFISATMHRYRLDRDDHVYQAFQHFDKDNDGHIT
KEELEMAMKEHGVGDEVSIKQIITEVDTDNDGKINFEEFRTMMRSGSSLQPQRELLPIK
>AtCPK32
MGNCCGTAGSLAQNDNKPKKGRKKQNPFSIDYGLHHGGGDGGGRPLKLIVLNDPTGREI
ESKYTLGRELGRGEFGVTYLCTDKETDDVFACKSILKKKLRTAVDIEDVRREVEIMRHMP
EHPNVVTLKETYEDEHAVHLVMELCEGGELFDRIVARGHYTERAAAAVTKTIMEVVQVC
HKHGVMHRDLKPENFLFGNKKETAPLKAIDFGLSVFFKPGERFNEIVGSPYYMAPEVLKR
NYGPEVDIWSAGVILYILLCGVPPFWAETEQGVAQAIIRSVLDFRRDPWPKVSENAKDLIR
KMLDPDQKRRLTAQQVLDHPWLQNAKTAPNVSLGETVRARLKQFTVMNKLKKRALRVI

AEHLSDEEASGIREGFQIMDTSQRGKINIDELKIGLQKLGHAIPQDDLQILMDAGDIDRDG
YLDCDEFIAISVHLRKMGNDEHLKKAFAFFDQNNNGYIEIEELREALSDELGTSEEVVDAII
RDVDTDKDGRISYEEFVTMMKTGTDWRKASRQYSRERFNSISLKLMQDASLQVNGDTR
>AtCPK33
MGNCLAKKYGLVMKPQQNGERSVEIENRRRSTHQDPSKISTGTNQPPPWRNPAKHSGAA
AILEKPYEDVKLFYTLSKELGRGQFGVTYLCTEKSTGKRFACKSISKKKLVTKGDKEDMR
REIQIMQHLSGQPNIVEFKGAYEDEKAVNLVMELCAGGELFDRILAKGHYSERAAASVCR
QIVNVVNICHFMGVMHRDLKPENFLLSSKDEKALIKATDFGLSVFIEEGRVYKDIVGSAYY
VAPEVLKRRYGKEIDIWSAGIILYILLSGVPPFWAETEKGIFDAILEGEIDFESQPWPSISNSA
KDLVRRMLTQDPKRRISAAEVLKHPWLREGGEASDKPIDSAVLSRMKQFRAMNKLKKLA
LKVIAENIDTEEIQGLKAMFANIDTDNSGTITYEELKEGLAKLGSRLTEAEVKQLMDAADV
DGNGSIDYIEFITATMHRHRLESNENVYKAFQHFDKDGSGYITTDELEAALKEYGMGDDA
TIKEILSDVDADNDGRINYDEFCAMMRSGNPQQPRLF
>AtCPK34
MGNCCSHGRDSDDNKEEPRPENGGGGVGAAEASVRASKHPPASPPPATKQGPIGPVLGRP
MEDVKSSYTLGKELGRGQFGVTHLCTQKATGLQFACKTIAKRKLVNKEDIEDVRREVQI
MHHLTGQPNIVELKGAYEDKHSVHLVMELCAGGELFDRIIAKGHYSERAAASLLRTIVQII
HTCHSMGVIHRDLKPENFLLLSKDENSPLKATDFGLSVFYKPGEVFKDIVGSAYYIAPEVL
RRKYGPEADIWSIGVMLYILLCGVPPFWAESENGIFNAILSGQVDFSSDPWPVISPQAKDLV
RKMLNSDPKQRLTAAQVLNHPWIKEDGEAPDVPLDNAVMSRLKQFKAMNNFKKVALRVI
AGCLSEEEIMGLKEMFKGMDTDNSGTITLEELRQGLAKQGTRLSEYEVQQLMEAADADG
NGTIDYGEFIAATMHINRLDREEHLYSAFQHFDKDNSGYITTEELEQALREFGMNDGRDIK
EIISEVDGDNDGRINYEEFVAMMRKGNPDPNPKKRRELSFK
>OsCPK1
MGNRTSRHHRAAPEQPPPQPKPKPQPQQQQQQWPRPQQPTPPPAAAPDAAMGRVLGRPM
EDVRATYTFGRELGRGQFGVTYLVTHKATGKRFACKSIATRKLAHRDDIEDVRREVQIMH
HLTGHRNIVELRGAYEDRHSVNLIMELCEGGELFDRIIARGHYSERAAAALCREIVAVVHS
CHSMGVFHRDLKPENFLFLSKSEDSPLKATDFGLSVFFKPGEHFKDLVGSAYYVAPEVLKR
NYGAEADIWSAGVILYILLSGVPPFWAESEDGIFDAVLRGHIDFSSEPWPSISNGAKDLVKK
MLRQDPKERLTSAEILNHPWIREDGEAPDKPLDITVISRMKQFRAMNKLKKVALKVVAEN
LSDEEITGLKEMFRSLDTDNSGTITLEELRSGLPKLGTKISESEIRQLMEAADVDGNGTIDY
AEFISATMHMNRLEKEDHILKAFEYFDKDHSGYITVDELEEALKKYDMGDDKTIKEIIAEV
DTDHDGRINYQEFVAMMRNNNPEIAPNRRRMF
>OsCPK2
MGNCCPGSGDAEPASSDASTGNGSSSFKAGASPSSAPAQNKPPAPIGPVLGRPMEDVRSIY
TIGKELGRGQFGVTSLCTHKATGQKFACKTIAKRKLSTKEDVEDVRREVQIMYHLAGQPN
VVELKGAYEDKQSVHLVMELCAGGELFDRIIAKGHYTERAAASLLRTIVEIIHTCHSLGVI
HRDLKPENFLLLSKDEDAPLKATDFGLSVFFKQGEVFKDIVGSAYYIAPEVLKRSYGPEAD
IWSVGVILYILLCGVPPFWAESEHGIFNSILRGQVDFTSDPWPRISASAKDLVRKMLNSDPK
KRISAYEVLNHPWIKEDGEAPDTPLDNAVMNRLKQFRAMNQFKKAALRVIAGCLSEEEIR
GLKEMFKSMDSDNSGTITVDELRKGLSKQGTKLTEAEVQQLMEAADADGNGTIDYDEFIT
ATMHMNRMDREEHLYTAFQYFDKDNSGCISKEELEQALREKGLLDGRDIKDIISEVDADN
DGRIDYSEFAAMMRKGNPEANPKKRRDVVI
>OsCPK3

MGNCCRSPAAAAREDVKSSHFPASAGKKKPHQARNGGVGGGGGGGGGGGGGGGAGQK
RLPVLGEEGCELIGGIDDKYALDRELGRGEFGVTYLCMDRDTKELLACKSISKRKLRTAVD
VEDVRREVAIMRHLPKSASIVSLREACEDEGAVHLVMELCEGGELFDRIVARGHYTERAA
ANVTRTIVEVVQLCHRHGVIHRDLKPENFLFANKKENSPLKAIDFGLSIFFKPGEKFSEIVG
SPYYMAPEVLKRNYGPEIDIWSAGVILYILLCGVPPFWAETEQGVAQAILRGNIDFKREPW
PNVSENAKDLVRRMLEPDPKLRLTAKQVLEHPWLQNAKKAPNVPLGDIVKSRLKQFSRM
NRFKRRALRVIADHLSAEEVEDIKEMFKAMDTDNDGIVSYEELKSGIAKFGSHLAESEVQ
MLIEAVDTNGKDALDYGEFLAVSLHLQRMANDEHLRRAFLFFDKDGNGYIEPEELREALV
DDGAGDSMEVVNDILQEVDTDKDGKISYDEFVAMMKTGTDWRKASRHYSRGRFNSLSM
KLIKDGSVKLVNE
>OsCPK4
MGACFSSHTATAAADGGSGKRQQRKGDHKGKLPDGGGGEKEKEAARVEFGYERDFEGR
YQVGRLLGHGQFGYTFAATDRASGDRVAVKRIDKAKMVRPVAVEDVKREVKILKELKGH
ENIVHFYNAFEDDSYVYIVMELCEGGELLDRILAKKNSRYSEKDAAVVVRQMLKVAAEC
HLHGLVHRDMKPENFLFKSTKEDSPLKATDFGLSDFIKPGKKFHDIVGSAYYVAPEVLKRR
SGPESDVWSIGVITYILLCGRRPFWNKTEDGIFREVLRNKPDFRKKPWPGISSGAKDFVKK
LLVKNPRARLTAAQALSHPWVREGGEASEIPVDISVLSNMRQFVKYSRFKQFALRALAST
LKEEELADLKDQFDAIDVDKSGSISIEEMRHALAKDLPWRLKGPRVLEIIQAIDSNTDGLV
DFEEFVAATLHIHQMAELDSERWGLRCQAAFSKFDLDGDGYITPDELRMVQHTGLKGSIE
PLLEEADIDKDGRISLSEFRKLLRTASMSNLPSPRGPPNPQPL
>OsCPK5
MGNTCGVTLRSKYFASFRGASQRHDEAGYAPVATSAAAAAAADEPAGKKAPRGSAAAA
DAPHAASMKRGAPAPAELTANVLGHPTPSLSEHYALGRKLGQGQFGTTYLCTDLATGVDY
ACKSIAKRKLITKEDVEDVRREIQIMHHLAGHRNVVAIKGAYEDPQYVHIVMELCAGGEL
FDRIIERGQFSERKAAELTRIIVGVIEACHSLGVIHRDLKPENFLLANKDDDLSLKAIDFGLS
VFFKPGQVFTDVVGSPYYVAPEVLRKCYGPEADVWTAGVILYILLSGVPPFWAETQQGIFD
AVLKGVIDFDSDPWPVISDSAKDLIRRMLNPRPKERLTAHEVLCHPWICDHGVAPDRPLDP
AVLSRIKQFSAMNKLKKMALRVIAESLSEEEIAGLKEMFKAMDTDNSGAITYDELKEGMR
KYGSTLKDTEIRDLMEAADVDNSGTIDYIEFIAATLHLNKLEREEHLVAAFSYFDKDGSGY
ITVDELQQACKEHNMPDAFLDDVIKEADQDNDGRIDYGEFVAMMTKGNMGVGRRTMR
NSLNISMR
>OsCPK6
MGNYYSCGASSTSSPTSPSLVDYYYCYHRYPSSCSSTSTATSSGGRMPIRSHQQRLSSPTAV
LGHETPALREVYTVGRKLGQGQFGTTYLCTQVSTGAEYACKSIAKRKLLSPEDVEDVRRE
IQIMHHLAGHGSVVTIQGAYEDNLYVHIVMELCEGGELFDRIVERGYFSERKAAEITRVIV
GVVEACHSLGVMHRDLKPENFLLKESSSSSSLKAIDFGLSVFFKPGQVFSDVVGSPYYVAP
EVLCKHYGPEADVWTAGVIVYILLSGVPPFWAETQQGIFDAVLRGSLDFDSDPWPTISDSA
KDLIRRMLRSPPRERLTAHQVLCHPWVCDDGVAPDRPLAPAVLSRLKQFSAMNRLKKMA
LRVIARNLSEEELAGLKEMFKAMDTDASGAITFDELKEGLRRYGSNLREAEIRDLMDAAD
VDKSGTIDYDEFIAATVHLNKLEREEHLLAAFAYFDRDGSGYITVDELEHACRDHNMADV
GIDDIIREVDQDNDGRIDYGEFVAMMKKGAIDIIGNGRLTIGRPTTATSDDPSPTISSSSR
>OsCPK7
MGNQCQNGTLGSDYHNRFPREHAVGYVQGDSYLDLKKFDDTWPEVNNFKPTAASILRR
GLDPTSINVLGRKTADLREHYIIGRKLGQGQFGTTYLCTEINTGCEYACKTIPKRKLITKED

VEDVRREIQIMHHLSGHKNVVAIKDVYEDGQAVHIVMELCAGGELFDRIQEKGHYSERK
AAELIRIIVSIVAMCHSLGVMHRDLKPENFLLLDKDDDLSIKAIDFGLSVFFKPGQVFTELV
GSPYYVAPEVLHKRYGPESDVWSAGVILYVLLSGVPPFWAETQQGIFDAVLKGHIDFQSDP
WPKISDSAKDLIRKMLSHCPSERLKAHEVLRHPWICENGVATDQALDPSVISRLKQFSAM
NKLKKLALRVIAERLSEEEIAGLREMFKAVDTKNRGVITFGELREGLRRFGAEFKDTEIGDI
MEAAHNDNNVTIHYEEFIAATLPLNKIEREEHLLAAFTYFDKDGSGYITVDKLQRACGEH
NMEDSLLEEIISEVDQNNDGQIDYAEFVAMMQGSNVGLGWQTMESSLNVHSLCWLFFQR
PVLRNESRHVCVTLISMLAFL
>OsCPK8
MGNCCGTPATAEEGGKRRRRGKQKKANPFTVAYNRAPSSAGAAAGRPGLMVLRDPTGR
DLGARYELGGELGRGEFGITYLCTEAETGDRYACKSISKRKLRTPVDVEDVRREVEIMRH
MPSHPNIVSLRAAYEDEDNVHLVMELCEGGELFDRIVARGHYTERAAAAVTRTIVEVVQM
CHRHGVMHRDLKPENFLYANKKDSSPLKAIDFGLSVFFRPGERFTEIVGSPYYMAPEVLK
RHYGPEVDVWSAGVILYILLCGVPPFWAETEQGVAQAIIRSVVDFKREPWPRVSEPAKDLV
KRMLDPNPMTRLTAEQVLEHPWLHDSKKMPDIPLGDAVRARLQQFAAMNKLKKKALKV
IAEHLSAEEAADIKDMFDKMDVSKNGQLTFEDFKAGIRKLGNQMPDSDLKILMDAADID
KNGILDYQEFVAVSIHVRKIGNDEHIQKAFSYFDQNKSGYIEIEELREALVDEIDGNDEDIIN
SIIRDVDTDKDGKISYDEFAVMMKAGTDWRKASRQYSRQRFSNLSLKLQKDGSISDDTQ
>OsCPK9
MGNTCCVAPATTDEVGAPPRDHHHAAKKSPAPSATTTTATRQRHGQEPKPKPKPRARAKP
NPYDWAPPRVLPARGGAAASAVRVLEGVVPHHPRLRVTDKYQLGRELGRGEFGVTHLAT
DRATRERLACKSIPKRRLRTAVDVADVRREVAIMASLPDHPALVRLRAAYEDADAVHLVM
ELCDGGELFDRIVARGRYTERAAAAAARTVAEVVRACHAHGVMHRDLKPENFLYAGKAE
DAQLKAIDFGLSVFFRPGERFREIVGSPYYMAPEVLRRDYGPEVDIWSAGVILYILLCGVPP
FWAETEQGVARAILRGAADFDREPWPRISRAAKSLVRQMLDVDPRRRPTAQQVLDHPWL
HHAARAPNVPLGDVVRARLKQFSLMNRLKKKAMRVIAEHLSVEEVEVIKDMFALMDTD
NNGRVTLQELKDGLTKVGSKLAEPEMELLMEAADVDGNGYLDYGEFVAVTIHLQRLSND
NHLRTAFLFFDKDGSGYIDRAELADALADDSGHADDAVLDHILREVDTDKDGRISYEEFV
AMMKSGTDWRKASRQYSRERFKTLSNSLIKDGSITMAR
>OsCPK10
MGNTCVGPSISKNGFFQSVSTVLWKARQDGDDALPGANGAPDGGGQGRLPAPPPPTSDA
PLAVQNKPPEHVKIVSTTDTASAEQDASKSSAGSDSGEAARPRPRVPPVKRVSSAGLLVGS
VLKRKTESLKDKYSLGRKLGQGQFGTTYLCVERATGKEFACKSILKRKLVTDDDVEDVR
REIQIMYHLAGHPNVISIRGAYEDAVAVHLVMELCAGGELFDRIVQKGHYTERKAAELAR
VIVGVVEVCHSMGVMHRDLKPENFLFADQTEEAALKTIDFGLSIFFRPGQVFTDVVGSPY
YVAPEVLKKKYGQEADVWSAGVIIYILLCGVPPFWAENEQGIFEEVLHGRLDFQSEPWPSI
SEGAKDLVRRMLVRDPKKRLTAHEVLRHPWVQVGGLAPDKPLDSAVLSRMKQFSAMNK
LKKMALRVIAENLSEDEIAGLKEMFKMIDTDNSGQITFEELKVGLKKVGANLQESEIYAL
MQAADVDNSGTIDYGEFIAATLHMNKIEREDHLFAAFQYFDKDGSGYITADELQLACEEF
GLGDVQLEEMIREVDEDNDGRIDYNEFVAMMQKPTMGLPAKKSGGLQNSFSIGFREALR
MS
>OsCPK11
MGNNCVGPSAAGQNGFFANVALWRPRPADAAPPALPPPSSAPSDQAPEPVTIPPSEHSSHH
SSRSTDPSTPTSAAEQPANKAAPKVKRVQSAGLLADSVLKRDVNTARLKDLYTIGKKLGQ

GQFGTTYLCVEKATGREFACKSIAKRKLLTQEDVEDVRREIQIMHHLAGHANVVSIVGAY
EDAVAVQLVMELCAGGELFDRIIQRGHYSEKAAAQLARVIVGVIEACHSLGVMHRDLKPE
NFLFIHQKEDSPLKAIDFGLSIFFKPGETFTDVVGSPYYVAPEVLMKHYGREVDVWSAGVI
IYILLSGVPPFWDESEQGIFEQVLKGDLDFSSEPWPNISESAKDLVRKMLIRDPKKRLTAHE
ALCHPWVCVDGVAPDKPLDSAVLSRLKQFSAMNKLKKMALRVIAESLSEEEIAGLKEMF
KMLDTDNSGHITLEELKTGLQRVGANLMDSEIDALMEAADIDNSGTIDYGEFIAATLHINK
VEKEDKLFAAFSYFDKDGSGYITQDELQKACEEFGIGDTRIEDIIGDIDQDNDGRIDYNEFV
EMMQKGNNAMGKMGQHSTGNFGLGEALKLR
>OsCPK12
MGNCFTKTYEIPITSGTMRRPASTAERSKARGGDEPGTWRRPSFPRHGAPPHRPPTGSSSA
AGALSRRASGGGGEMGPVLQRAMVSVRSLYQLDRKLGSGQFGTTYLCTERATGNRYAC
KSVSKRKLVRRTDVDDVRREITILQHLSGQPNIAEFRGAYEDNDHVHLVMEFCSGGELFD
RITAKGSYSERQAAAVCRDILTVVHVCHFMGVIHRDLKPENFLLASADDDAPLKAIDFGLS
VFIEEGKVYKDIVGSAYYVAPEVLQRNYGKEADIWSAGVILYILLCGTPPFWAETEKGIFD
AILVNQVDFSTSPWPSISESAKDLIRQMLHRDPQKRITASQALEHRWLKEGGASDRPIDSAV
LSRMKQFKAMNKLKQLALKVIAENLSPEEIKGLKQMFNNMDTDRSGTITVEELKVGLTK
LGSRISEAEVQKLMEAVDVDKSGSIDYSEFLTAMINKHKLEKEEDLLRAFQHFDKDNSGYI
TRDELEQAMAEYGMGDEANIKQVLDEVDKDKDGRIDYEEFVEMMRKGIQT
>OsCPK13
MGNACGGSLRSKYLSFKQTASQRHDTDDNNNAAAADSPKKPSRPPAAAKTDDHPVSASA
PAAAMRRGQAPADLGSVLGHPTPNLRDLYAMGRKLGQGQFGTTYLCTELSTGVDYACKS
ISKRKLITKEDIEDVRREIQIMHHLSGHKNVVAIKGAYEDQLYVHIVMELCAGGELFDRIIQ
RGHYSERKAAELTRIIVGVVEACHSLGVMHRDLKPENFLLANKDDDLSLKAIDFGLSVFF
KPGQTFTDVVGSPYYVAPEVLLKHYGPEADVWTAGVILYILLSGVPPFWAETQQGIFDAVL
KGFIDFDSDPWPVISESAKDLITKMLNPRPKERLTAHEVLCHPWIRDHGVAPDRPLDPAVLS
RIKQFSAMNKLKKMALRVIAESLSEEEIAGLKEMFQTMDADNSGAITYDELKEGLRKYGS
TLKDTEIRDLMDAADIDNSGTIDYIEFIAATLHLNKLEREEHLVAAFSYFDKDGSGYITVDE
LQQACKEHNMPDAFLDDVINEADQDNDGRIDYGEFVAMMTKGNMGVGRRTMRNSLNIS
MRDAPGAL
>OsCPK14
MGNCCPPGSSSEPDPPPASSGSSRPAGSAGAAASPATISPSAAPAPAKPPAPIGPVLGRPMED
VKSIYTVGKELGRGQFGVTSLCTHKATGQRFACKTISKRKLSTKEDVEDVRREVQIMYHL
AGQPGVVELKGAYEDKHAVHLVMELCAGGELFDRIIAKGHYTEHAASSLLRTIVEIIHTCH
SMGVIHRDLKPENFLLLSKDEHAPLKATDFGLSVFFKEGEVFRDIVGSAYYIAPEVLKRSY
GPEADIWSIGVMLYILLCGVPPFWAESEHGIFNSILRGHVDFSSEPWSRISHGAKDLVRRML
HSDPKQRISAYDVLNHPWIKEDGEAPDTPLDNAVLGRLKQFRAMNQFKKAALRVIAGCLS
EEEIRGLKEMFKSMDSDNSGTITVDELRKGLAKKGTKLTEAEVQQLMEAADADGNGTID
YEEFITATMHMNRMDREEHLYTAFQYFDKDNSGYITIEELEQALREKGLMDGREIKDIISE
VDADNDGRINYTEFVAMMRKGDPEANPKKRRDVVL
>OsCPK15
MVSSSSSPQSKPPKPKPKPLSPPMGARASRHRQSPDQSQSQSPSPHHKHHHHHQTTRAPKP
KPKPQPPPPQQPRSQPPPPPRHQPQQAPQQAAAEDGVGRVLGRPMEDVRATYTFGRELGR
GQFGVTYLATHKPTGRRYACKSIAARKLARPDDLDDVRREVHIMHHLTGHRNIVELRGAY
EDRHSVNLVMELCEGGELFDRIIARGHYSERAAAALCREIVSVVHSCHSMGVMHRDLKP

ENFLFLNKREDSPLKATDFGLSVFFKPGEQFRDLVGSAYYVAPEVLKRLYGAEADIWSAG
VILYILLSGVPPFWAENEDGIFDAVLQGHIDFSSEPWPSISSGAKDLVKRMLRQDPKERLTA
AEILNHPWIREDGEAPDKPLDITVISRMKQFRAMNKLKKVALKVVAENLSEEEIVGLKEM
FKSLDTDNSGTITLEELRAGLPKLGTKISESELRQLMEAADVDGNGSIDYVEFISATMHMN
RLEKEDHIYKAFEYFDKDHSGFITVDELEEALTKYDMGDEATIKEIIAEVDTDHDGRINYQ
EFVAMMKNNSPEIVPNRRRMF
>OsCPK16
MGNCCRSPAAAAREDVKTSHFPASTGGGKKKPHQARNGGGGGGGGGGGWEKKRLSV
LGEEGSEVNGGIEEKYALDRELGRGEFGVTYLCMDRCSRELLACKSISKRKLRTPVDVED
VRREVAIMRHLPRSASIVSLREACEDDGAVHLVMELCEGGELFDRIVARGHYTERAAAAV
TRTIVEVVQLCHRHGVIHRDLKPENFLFANKKENSPLKAIDFGLSIFFKPGEKFSEIVGSPYY
MAPEVLKRNYGPEIDIWSAGVILYILLCGVPPFWAETEQGVAQAILRGNIDFKREPWPNVS
DNAKDLVRQMLQPDPKLRLTAKQVLEHTWLQNAKKAPNVPLGDIVKSRLKQFSRMNRF
KRRALRVIADHLSAEEVEDIKDMFKVMDTDNDGIVSYEELKSGIAKFGSHLAESEVQMLI
EAVDTNGRGALDYGEFLAVSLHLQRMANGEHLRRAFLFFDKDGNGYIEPEELQEALVED
GATDIMEVVKDILQEVDTDKDGKISYEEFVAMMKTGTDWRKASRHYSRGRFNSLSIRLIK
DG
SVKLGNE
>OsCPK17
MGNTCVGPSSAADRHGFFHSVSLAVLWRPGGRAEPSQPPGYPPRESSHSSVTSSTAPERVT
IADSDLSSSTPNKGGNKPKVRRVQSAGLLADSVLKRDSERLKDLYTLGKKLGQGQFGTTY
QCVEKATGKVLACKSIAKRKLVSEEDVEDVRREIQIMHHLAGHPSVVSIVGAYEDAVAVH
LVMELCAGGELFDRIVQRGHYSEKAAAQLARVIIGVVEACHSLGVMHRDLKPENFLFVN
HKEDSPLKTIDFGLSIFFKPGENYSDVVGSPYYVAPEVLMKHYGREVDVWSAGVIIYILLS
GVPPFWDESEQGIFEKVLKGDLDFSSDPWPAISDSAKDLVRKMLNRDPRKRLTAHEALCH
PWVCVDGVAPDKPLDSAVLTRLKQFSAMNKLKKMALRVIAENLSEDEIAGLREMFKMLD
TDNSGQITLEELKTGLRRVGANLKDSEITTLMEAADIDNSGSIDYGEFIAATMHLNKVERE
DNLFAAFSYFDKDSSGYITQDELQKACEEFGIGDAHLEDIIKDIDQDNDGRIDYNEFVTMM
QKGNNPLGKKGQGQLSFGLREALKLG
>OsCPK18
MGLCSSSSARRDAGTPGGGNGAGNKDNAGRKGIVACGKRTDFGYDKDFEARYALGKLL
GHGQFGYTFAAVDRRSSERVAVKRIDKNKMVLPVAVEDVKREVKILKALQGHENVVHFY
NAFEDDNYVYIVMELCEGGELLDRILAKKDSRYSEKDAAVVVRQMLKVAAECHLHGLVH
RDMKPENFLFKSTKEDSSLKATDFGLSDFIRPGKHFRDIVGSAYYVAPEVLKRKSGPESDV
WSIGVITYILLCGRRPFWDKTEDGIFKEVLKNKPDFRRKPWPNITPCAKDFVQKLLVKDPR
ARLTAAQALSHEWVREGGQASDIPLDISVLHNMRQFVKYSRFKQFALRALASTLNAEELS
DLRDQFNAIDVDKNGTISLEELKQALAKDVPWRLKGPRVLEIVEAIDSNTDGLVDFEEFVA
ATLHVHQLVEHDTEKWKSLSQAAFDKFDVDGDGYITSDELRMQTGLKGSIDPLLEEADID
RDGKISLDEFRRLLKTASMSSRNVQTPRSVHRS
>OsCPK19
MGSCCSRATSPDSGRGGANGYGYSHQTKPAQTTPSYNHPQPPPPAEVRYTPSAMNPPVVP
PVVAPPKPTPDTILGKPYDDVRSVYSLGKELGRGQFGVTYLCTEIASGKQYACKSISKRKL
VSKADKEDIRREIQIMQHLSGQQNIVEFRGAYEDKSNVHVVMELCAGGELFDRIIAKGHY
SERAAATICRAVVNVVNICHFMGVMHRDLKPENFLLATKEENAMLKATDFGLSVFIEEGK

MYRDIVGSAYYVAPEVLRRNYGKEIDVWSAGVILYILLSGVPPFWAETEKGIFDAILQGEID
FESQPWPSISESAKDLVRKMLTQDPKKRITSAQVLQHPWLRDGEASDKPIDSAVLSRMKQF
RAMNKLKKMALKVIASNLNEEEIKGLKQMFTNMDTDNSGTITYEELKAGLAKLGSKLSE
AEVKQLMEAADVDGNGSIDYVEFITATMHRHKLERDEHLFKAFQYFDKDNSGFITRDELE
SALIEHEMGDTSTIKDIISEVDTDNDGRINYEEFCAMMRGGGMQQPMRLK
>OsCPK20
MGNCCVTPEGSGRGRKKQQQEQKQKQKEPKQQQQQQKKGKKPNPFSIEYNRSSAPSGH
RLVVLREPTGRDIAARYELGGELGRGEFGVTYLCTERETGDAYACKSISKKKLRTAVDIED
VRREVDIMRHLPKHPNIVTLRDTYEDDNAVHLVMELCEGGELFDRIVARGHYTERAAALV
TRTIVEVVQMCHKHGVMHRDLKPENFLFANKKETAALKAIDFGLSVFFTPGERFTEIVGSP
YYMAPEVLKRNYGPEVDVWSAGVILYILLCGVPPFWAETEQGVAQAIIRSVIDFKRDPWP
RVSDNAKDLVKGMLNPDPRRRLNAQQVLDHPWLQNIKKAPNVNLGETVKARLQQFSVM
NKFKKHALRVIAEHLSVEEVAGIKDMFEKMDLNKDNMINFDELKLGLHKLGHQMADAD
VQILMDAADVDGNGSLDYGEFVALSVHLRKIGNDEHLHKAFAYFDRNQSGYIEIDELRES
LADDLGANHEEVINAIIRDVDTDKDGKISYDEFAAMMKAGTDWRKASRQYSRERFTSLS
LKLQKDGSLQLTTTQ
>OsCPK21
MGGCYSAYASSRKLRGRISKISLVIPDPVPDAEAASPRKDGVDGDGDDVRGGGGGCDDG
GDVVAIATTTADEFARRYVLGKELGRGEFGVTRRCSDAATGEALACKTIRKHRRLAPPRVT
AAKAAAAHGEDVKREVAIMRRMSSASSSRGGGAASSAAVVRLREACEDAADGSVHLVM
ELCEGGELFDRIVARGHYSERAAANIFRTIVDVVQLCHSNGVIHRDLKPENFLFANKSEDS
PLKVIDFGLSVFFKPGDRFTEVVGSAYYMAPEVLRRSYGPEVDVWSAGVILYILLCGVPPF
WGDNDEKIAQAILRGAIDFNREPLPRVSANAKDLVRRMLDPNPSTRLTAKQVLEHPWLKN
ADTAPNVSLGDAVRARLQQFSAMNKFKKKALGVVARNLPGEEVDKYVQMFHHMDKDK
NGHLSLDELLEGLHINGQPVPEPEIRMLLEAADTDGNGTLDCDEFVTVSVHLKKMSNDEY
LAAAFNYFDKDGSGFIELDELREEVGPNEQAILEILRDVDTDKDGRISYQEFELMMKSGA
DWRNASRHFSRANFSTLSRRLCKDTLTP
>OsCPK22
MGGCSSAFAVSTRMIRFSRGRVPAAILPVTSNDEPCCSCSPENNNKNNDGGGGGCDGGEH
QKGKSWRRWQYRRCGGGGGGGGGRKNAILGDAADVKTAAGFAERYRLGAELGRGEFG
VTRRCSDAATGEALACKTIRRKRLRRCRGDAEDVRREVEILRRISALGAGADSVVRLRDA
CEDSDGVHLVMELCEGGELFDRIFARGHYTERAAAKLARTIVGVVQLCHENGVMHRDLK
PENFLFANKSEDSPLKAIDFGLSVFFKPGERFTQVVGSTYYMAPEVLNRSYGPEADVWSA
GVILYILLCGVPPFWGDNDEKTVTAILQGGINFQREPWPKVSPHAKDLVSKMLDPDPSTRL
TAKEVLEHPWLKNADRAPNVSLGEIVRSRLMQFSAMNKFKKKALGVVAKNLPVEEMDK
YTQMFHKMDKDNSGNLTLEDLKLGLQINGHPVPETEIEMLLEAGDIDGNGTLDCEEFVTV
LLHIKKMSNEEYLPKAFKFFDKDGNGFIEMEELMDALGDELGPTEQVVKDIIRDIDTDKD
GRISYQEFESMMISGSDWRNASRRYSKANFSSLSRKLCKGNS
>OsCPK23
MGNSCQNGTYGNNYQNSNRFQNDRFASRYVDGNDTEDCYSGSSRASLAGALRQGLNLK
SPVLGYKTPNVRELYTLGRELGQGQFGKTYLCTEISTGCQYACKTILKSNLRCVSDIEDVR
REIQIMHHLSGQKNIVTIKDTYEDEQAVHIVMELCAGGELFSKIQKRGHYSERKAAELIKII
VGIIETCHSHGVMHRDLKPENFLLLDADDEFSVKAIDFGLSVFFRPGQVFREVVGSPYYIA
PEVLEKRYGPEADIWTAGVILYVLLTGVPPFWADTQSGIYEKVLDGRIDFKSNRWPRISDS

AKDLIKKMLCPYPSERLKAHEVLKHPWICDNGVATNRALDPSVLPRLKQFSAMNRLKKLS
LQIIAERLSEEEIVGLREMFKAMDTKNRSVVTFGELKGLKRYSSVFKDTEINDLMEAADD
TTSTINWEEFIAAAVSLNKIEREKHLMAAFTYFDKDGSGFITVDKLQKACMERNMEDTFL
EEMILEVDQNNDGQIDYAEFVTMMQSNNFGLGWQTVESSLNVALREAPQVY
>OsCPK24
MQPDPSGSGGDGNANAKAKLAPPPVTAAGGRPVSVLPHKTANVRDHYRIGKKLGQGQF
GTTYLCVDKASGGEFACKSIPKRKLLCREDYEDVWREIQIMHHLSEHPNVVRIRGAYEDA
LFVHIVMELCAGGELFDRIVAKGHYTERAAAQLIRTIVAVVEGCHSLGVMHRDLKPENFLF
ASAAEDAPLKATDFGLSMFYKPGDKFSDVVGSPYYVAPEVLQKCYGPESDVWSAGVILYI
LLCGVPPFWAETEAGIFRQILRGKLDFESEPWPSISDSAKDLVRNMLCRDPTKRLTAHEVL
CHPWIVDDAVAPDKPIDSAVLSRLKHFSAMNKLKKMALRVIAESLSEEEIGGLKELFKMID
TDDSGTITFDELKEGLKRVGSELTEHEIQALMEAADIDNSGTIDYGEFIAATLHMNKLEREE
NLVSAFSFFDKDGSGFITIDELSQACREFGLDDLHLEDMIKDVDQNNDGQIDYSEFTAMM
RKGNAGGAGRRTMRNSLQLNLGEILNPSNS
>OsCPK25
MGQCCTGGGKAVAGDEAEPGTSKAAPPSRGTSSKNGSAKQQPCSPAAKAAATEAAAAAS
SSKKPAGPIGEVLERPMEEVRTTYSIGKELGRGQFGVTHLCTHKATGEKLACKTIAKRKLA
NKEDVDDVRREVQIMHHLSGQPNIVDLRGAYEDKHNVHLVMELCAGGELFDRIIARGHY
TERAAAALLRAIVGIVHTCHSMGVIHRDLKPENFLLLSKGDDAPLKATDFGLSVFFKEGE
VFRDIVGSAYYIAPEVLKRKYGPEADIWSIGVMLYIFLAGVPPFWAESENAIFTAILRGQIDL
ASEPWPKISSGAKDLVRKMLNINPKERLTAFQVLNHPWIKEDGDAPDVPLDNVVLNRLKQ
FRAMNQFKKAALRIIAGCLSEEEIKGLKEMFKNIDKDNSGTITLEELKNGLAKQGTKFSDN
EIEQLMEAADADGNGIIDYEEFVTATVHMNKMDREEHLYTAFQYFDKDNSGYITKEELEQ
ALKEQGLYDANEIKDVITDADSNNDGRIDYSEFVAMMRKGSGCAEATNPKKKRRDLVL
>OsCPK26
MGQCCTGGGKAVAGDEAEPGTSKAAPPSRGTSSKNGSAKQQPCSPAAKAAATEAAAAAS
SSKKPAGPIGEVLERPMEEVRTTYSIGKELGRGQFGVTHLCTHKATGEKLACKTIAKRKLA
NKEDVDDVRREVQIMHHLSGQPNIVDLRGAYEDKHNVHLVMELCAGGELFDRIIARGHY
TERAAAALLRAIVGIVHTCHSMGVIHRDLKPENFLLLSKGDDAPLKATDFGLSVFFKEGE
VFRDIVGSAYYIAPEVLKRKYGPEADIWSIGVMLYIFLAGVPPFWAESENAIFAAILRGQID
LASEPWPKISSGAKDLVRKMLNINPKERLTAFQVLNHPWIKEDGDAPDVPLDNVVLNRLK
QFRAMNQFKKAALRIIAGCLSEEEIKGLKEMFKNIDKDNSGTITLEELKNGLAKQGTKFSD
NEIEQLMEAADADGNGIIDYEEFVTATVHMNKMDREEHLYTAFQYFDKDNSGYITKEELE
QALKEQGLYDANEIKDVITDADSNNDGRIDYSEFVAMMRKGSGCAEATNPKKKRRDLVL
>OsCPK27
MGNVCIGPRRNFAKNGLLGILRPRHAAPSSPSQPTTTSRSIPVVLPSAPSSKPPPPTQTAPPV
PVVISEPPPPQPQPEPQPAAPSQPPPPQEQPSPPPPASSNTTQQPPPPQQRQQSRAKKPAHIKR
ISSAGLQVESVLRRKTENLKDKYSLGRKLGQGQFGTTYLCVDKANGGEYACKSIAKRKLL
TDEDVEDVRREIQIMHHLAGHPNIISIRGAYEDAVAVHVVMELCAGGELFDRIVRKGHYTE
RQAAGLARVIVAVVESCHSLGVMHRDLKPENFLFVGNEEDAPLKTIDFGLSMFFRPGEVF
TDVVGSPYYVAPEVLKKSYGQEADVWSAGVIIYILLCGVPPFWAETEQGIFEQVLHGTLDF
ESDPWPNVSDGAKDLLRKVLVRDPKKRLTAHEVLCHPWLQMSGSAPDKPLDSAVLSRLR
QFSAMNKLKKMALRVIAENLSEEEIAGLKEMFKMMDTDNSGQINYEELKAGLERVGAN
MKESEIYQLMQAADIDNSGTIDYGEFIAATLHLNKVEREDHLYAAFQYFDKDGSGYITSDE

LQQACDEFGIEDVRLEDMIGEVDQDNDGRIDYNEFVAMMQKTTTGFGKKGGHNFSGFRD
ALKSHS
>OsCPK28
MQPDPQPHGRGREKAAGAGPRLPPPVTAPSVGRPASVLPHKTANVRDHYRIGKKLGQGQ
FGTTYLCVGKPDGGEYACKSIPKRKLLCREDYEDVWREIQIMHHLSEHPNVVRIRGAYED
ALFVHIVMELCAGGELFDRIVAKGHYTERAAALLIRTIVGVVEGCHSLGVMHRDLKPENF
LFASTAEDAPLKATDFGLSVFYKPGDKFSDVVGSPYYVAPEVLQKIYGPEADVWSAGVIL
YILLCGVPPFWAETESGIFRQILRGKLDLESDPWPSISDSAKDLVRNMLIRDPTKRFTAHEV
LCHPWIVDDAVAPDKPIDSAVLSRLKHFSAMNKLKKMALRVIAESLSEEEIGGLKELFKMI
DTDNSGTITYDELKNGLKRVGSDLMEPEIQALMDAADIDNSGTIDYGEFLAATLHMNKLE
REENLVSAFTFFDKDGSGFITIDELSQACEQFGLSDVHLEDMIKDVDQNNDGQIDYSEFAA
MMRKGNAGGANAGGVTSTGGTGRRTMRNSLRVNLGDILKPNEN
>OsCPK29
MGNCCVSRPSGADKRRRCGSSTAPHTRGGRRVIGAANMRCLSTVSSVSDAARAVMSNEP
ATVLGNSGSSGNGGVMAAEEMLRRYEIGEELGRGEFGVTRRCRDAVTGERLACKSISKRK
LRSSVDVEDVRREVAIMRSLPAHANVVRLREAFEDADAVHLVMEVCEGGELFDRIVARGH
YTERAAAAVMRTIMDVVQHCHKNGVMHRDLKPENFLYANASENSPLKVIDFGLSVCFKP
GARFNEIVGSPYYMAPEVLKRNYGQEIDIWSAGVILYILLCGVPPFWAETDEGIAQAIIRSH
IDFQREPWPKVSDNAKDLVRRMLDPNPYTRLTAQQVLEHPWIQNASAAPNIPLGEAVRSR
LKQFTVMNKFKKKALLVVAEYLPTEELDAIRELFNMLDTKKKGHLTLEELRKGLQVIGHN
IHDTDVDMLMEAADIDGNGILDCKEFVTVSIHLKKIRSDEHLPKVFSFFDKNGSGYIEIEEL
KEALSPRGDQKSIDDIFLDVDIDKDGKISYEEFELMMSAGMDWRNASRQYSRAVYNTLSR
KIFKEVSLKLDHSGPLVAAGK
>OsCPK30
MGLCHGKPSQIPEPEAEEAAAAAGVAVAGAASPGPAAAAAAAAAAKPGTPKQPKFPFYLP
SPLPASSYKSSPANSSVASTPARGGLKRPFPPPSPAKHIRALLARRHGSVKPNEAPIPEGGET
EVGLDKGFGFSKHFFAKYELGDEVGRGHFGYTCSAKAKKGDHKGHDVAVKVIPKAKMT
TAIAIEDVRREVRILSSLTGHSNLVQFYDAFEDEDNVYIVMELCKGGELLDRILARGGKYS
EEDAKVVMVQILSVVSFCHLQGVVHRDLKPENFLFTSKDENSALKVIDFGLSDFVKPDER
LNDIVGSAYYVAPEVLHRSYGTEADMWSIGVIAYILLCGSRPFWARTESGIFRAVLKAEPSF
DEAPWPTLTAEAKDFVKRLLNKDYRKRMTAAQALSHPWIRNSQQVKIPLDMIIYKLMRA
YISSSSLRKSALRALAKTLTANQLFYLREQFELLGPNKNGYISLQNLKTALVKNSTDAMKD
SRVIDFVNTVCTLQYRKLDFEEFAASAVSVYQMEALETWEQHARRAYELFDKEGNRPIVI
EELASELGLGPSVPLHVVLQDWIRHADGKLSFLGFIKLLHGVSSRSIPKA
>OsCPK31
MRGLVLQGRQQSLQGRLRSSSKFCVSRPVAPRPCKACRRAARSGSSSCSRAVAVQVVASL
DYIATASDEGVLKLPARTELDPEEIKSVFGYPRNLLDSYYLGRVIGAGSFGVVREGIEVSTG
RRFAVKTVSKVPKRGSPTPRYLLKLRAEVEVMQQLGVSLNAVHLHDVFEDDVNVHMVM
ELCEGGALLERVESGVYSELYISKLVRSILRFIAQCHAKGIIYRDVNPDNFLFLTAEEDSPLK
ATDFGLSIRHYSHEPKLTSRSGTPAYMAPELVMQCYDEKADLWSVGMLAYQLLTGRFPFW
EDVRNETLSDVWKAILSSEIDWNAPELQPLSSAARDLLERLLQRNPVMRPSAAEALEHPW
LAQEGAANDMPLKGSVVQRLQRFATYTHLKQVVLRMITEDMRQRGKAPSFNSALQELFA
AYDKDKSGTISFEELAEGLRGQGYVVNESEVRQLMEKMDMDHDGNVGGDEFLATLIDW
GQVMQEQEWQSYVDQAFNRMDLDGDGFIDLDELLSELPAAYFHEPSSEDERISEAKRML

READENGDGRISKQEFYNLLRDNVAPDSLSMYDDRLSHNVAAMSV
>ZmCPK1
MGNACSGALRSKYLHSFKHAASQRHDSEYSASADDSPRKPTPPATTDAHAPAPPAAAMR
RGAAGATPDLGSVLGHPTPNLRDLYALGRKLGQGQFGTTYLCTELATGIDYACKSISKRKL
ITKEDVDDVRREIQIMHHLSGHKNVVAIKGAYEDQVYVHIVMELCAGGELFDRIIQRGHY
SERKAAALTRIIVGVVEACHSLGVMHRDLKPENFLLANRDDDLSLKAIDFGLSVFFKPGQ
VFTDVVGSPYYVAPEVLLKSYGPAADVWTAGVILYILLSGVPPFWAETQQGIFDAVLKGAI
DFDSDPWPVISDSAKDLIRRMLNPRSAERLTAHEVLCHPWIRDHGVAPDRPLDPAVLSRIK
QFSAMNKLKKMALRVIAESLSEEEIAGLKEMFQIMDTDNSGAITYDELKEGLRKYGSTLK
DTEIRDLMEAADIDNSGTIDYIEFIAATLHLNKLEREEHLVAAFSYFDKDGSGYITVDELQQ
ACKEHNMPDAFLDDVINEADQDNDGRIDYGEFVAMMTKGNMGVGRRTMRNSLNISMR
DAPGAF
>ZmCPK2
MRRGGAGAPPDLGSVLGHTTPNLRDLYALGRKLGQGQFGTTYLCTELATGIDYACKSISK
RKLITKEDVDDVRREIQIMHHLSGHKNVVAIKGAYEDQVYVHIVMELCAGGELFDRIIQR
GHYSERKAAALTRIIVGVVEACHSLGVMHRDLKPENFLLANRDDDLSLKAIDFGLSVFFK
PGQVFTDVVGSPYYVAPEVLLKSYGPAADVWTAGVILYILLSGVPPFWAETQQGIFDAVLK
GAIDFDSDPWPVISDSAKDLIRRMLNPRPAERLTAHEVLCHPWIRDHGVAPDRPLDPAVLS
RIKQFSAMNKLKKMALRVIAESLSEEEIAGLKEMFQTMDTDNSGAITYDELKEGLRKYGS
TLKDTEIRDLMDAADIDNSGTIDYIEFIAATLHLNKLEREEHLVAAFSYFDKDGSGYITVDE
LQLACKEHNMPDAFLDDVINEADQDNDGRIDYGEFVAMMTKGNMGVGRRTMRNSLNIS
MRDDLVCSET
>ZmCPK3
MGNACGGALRSKYQHSFKHAASSQRRASSEYSASAVADDSPRKPTQPPATTDAHAPPPPP
ATAAAAMRRGGAGAPPDLGSVLGHPTPNLRDLYALGRKLGQGQFGTTFLCTELATGVDY
ACKSISKRKLITREDVDDVRREIQIMHHLSGHTNVVAIKGAYEDQLYVHIVMELCAGGELF
DRIIQRGHYSERKAAELTRIIVGVVEACHSLGVMHRDLKPENFLLVNKDDDLSLKAIDFGL
SVFFKPGQVFTDVVGSPYYVAPEVLLKNYGPAADVWTAGVILYILLSGVPPFWAETQQGIF
DAVLKGVIDFDSDPWPVISDSAKDLIRRMLNPRSAKRLTAHEVLCHPWIRDHGVAPDRPL
DPAVLSRIKQFSAMNKLKKMALRVIAESLSEEEIAGLKEMFQTMDTDNSGAITYDELKEGL
RKYGSTLKDTEIRDLMDAADIDNNGTIDYIEFIAATLHLNKLEREEHLVAAFSYFDKDGSG
YITVDELQQACKEHNMPDAFLDDVINEADQDNDGRIDYGEFVAMMTKGNMGVGRRTM
RNSLNISMRDAPGAF
>ZmCPK4
MGNACGGALRSKHQHSFKHAASSQRHHASSEYSASAAADDSPRKPPPPPATTDAHAPPPP
PATAMRRGGAGAPPDLGSVLGHPTPNLRDLYALGRKLGQGQFGTTFLCTELATGVDYACK
SISKRKLITREDVDDVRREIQIMHHLSGHTNVVAIKGAYEDQLYVHIVMELCAGGELFDRII
QRGHYSERKAAELTRIIVGVVEACHSLGVMHRDLKPENFLLVNKDDDLSLKAIDFGLSVF
FKPGQVFTDVVGSPYYVAPEVLLKNYGPAADVWTAGVILYILLSGVPPFWAETQQGIFDA
VLKGVIDFDSDPWPVISDSAKDLIRRMLNPRSAERLTAHEVLCHPWIRDHGVAPDRPLDPA
VLSRIKQFSAMNKLKKMALRVIAESLSEEEIAGLKEMFQTMDTDNSGAITYDELKEGLRK
YGSTLKDTEIRDLMDAADIDNNGTIDYIEFIAATLHLNKLEREEHLVAAFSYFDKDGSGYIT
VDELQQACKEHNMPDAFLDDVINEADQDNDGRIDYGEFVAMMTKGNMGVGRRTMRNS
LNISMRDAPGAF

>ZmCPK5
MGNTCGVTLRSKYFASFRGSASQRRDAPGYSPVATSAAADEPSPHGNGKRATRPSQAAAA
ADGSAPPPAPGMRRGVPAPAELTANVLGHPTPSLRDHYALGRKLGQGQFGTTYLCTDLAT
GVDYACKSIAKRKLITKEDVEDVRREIQIMHHLAGHRNVVAIKGAYEDQLYVHIVMEFCA
GGELFDRIIQRGHYSERKAAELTRIIVGVVEACHSLGVMHRDLKPENFLLSNKDDDMSLK
AIDFGLSVFFKPGQIFTDVVGSPYYVAPEVLRKRYGPEADVWTAGVILYILLCGVPPFWAE
TQQGIFDAVLKGVIDFDLDPWPVISESAKDLIRRMLNPIPSERLTAHEVLCHPWICDHGVAP
DRPLDPAVLSRIKQFSAMNKLKKMALQVIAESLSEEEIAGLKEMFMAMDTDNSGAITYDE
LKEGLRKYGSTLKDTEIRDLMEAADIDNSGTIDYIEFIAATLHLNKLEREEHLVAAFSYFDK
DGSGYITVDELQQACKEHNMPAAFLDDVIKEADQDNDGRIDYGEFVAMMTKGNMGVGR
RTMRNSLNISMRDTPAGAL
>ZmCPK6
MGGHQLHLSSPTAVLGHVTPALRDLYAVGRKLGQGQFGTTYLCTELSTGAAFACKSIAKR
KLLTPEDVDDVRREIQIMHHLAGHKSVVTIKGAYEDPLYVHIVMELCEGGELFDRIVDRG
YFSERKAAEIARVIVGVVEACHSLGVMHRDLKPENFLLKDRGHDASLKAIDFGLSVFFKP
GQVFTDVVGSPYYVAPEVLCKHYGPEADVWTAGVIVYILLSGVPPFWAETQQGIFDAVLK
GAIDFDSEPWPAISDSAKDLIRRMLRSPPADRLSAHQVLCHPWICENGVAPDRALDPAVLT
RLKQFSAMNRLKKMALRVISQSLSEEELAGLKEMFKAMDTDGSGAITFDELKEGLKRHG
SKDLRESEIRDLMDAADVDKSGSIDYDEFIAATVHMSKLEREEHLLAAFAYFDKDGSGYIT
VDELEQACREHNMADVGLDDIITEVDQDNDGRIDYGEFVAMMKKGIIGHGRLTMRHTSD
GSVLHGAG
>ZmCPK7
MGNQCPNGTLGSDYYNRPTSRFADGCLEDDRYSDLKKFDKPWPEVNSFKPTAVGILKRG
LDPTSITVLERKTADIREHYIIGRRLGQGQFGTTFLCTEISTGCEYACKTIPKRKLITKEDVE
DVRREIQIMHHLSGHKNVVSIKDVYEDGQAVYIVMELCAGGELFDRIQEKGHYSEQKAA
ELIRIIISLVAMCHSLGVMHRDLKPENFLLWDKEDDLSIKAIDFGLSVFFKPGQVFTELVGSP
YYVAPEVLHKRYGPEADVWSAGVILYVLLSGVPPFWAETQQGIFDAVLKGHIDFDSDPWP
KISESAKNLIRKMLCPCPSERLKAHEVLRHPWICENGVATDQALDPGVLSRLKQFSTMNK
LKKLALRVIAERLSEEEIAGLRQMFKAVDVQNRGVITFGELRQGLKRYGSELENREISDIM
EVADNDNNVTINYEEFIAATVPLNKIEREEHLMAAFTYFDKDGSGYITVDKLQRACGEHD
MDDTFLEEIILEVDQNNDGQIDYAEFVAMMQGSKVGLGWQQMETTLNVTLRDAPQVHC
H
>ZmCPK8
MGNTCVGPSAAGRNGFLANVTLWRPRGDDPTPAPALPPPSSPASDKAPDPVTIPESKPSSH
HSSRSTDLPPAPASQPQAQAQDNTPAKQPAPKVKRVQSAGLLADSVLKRDVNTARLKDLY
TIGKKLGQGQFGTTYLCVEKATGREFACKSIAKRKLITEEDVEDVRREIQIMHHLAGHAN
VVSIVGAYEDAVAVQLVMELCAGGELFDRIIKRGHYSEKAAAQLARVIVGVVESCHSLGV
MHRDLKPENFLFVNQKEDSPLMTIDFGLSIFFKPGEMFTDVVGSPYYVAPEVLLKYYGRE
VDVWSAGVIIYILLSGVPPFWDESEQGIFEQVLRGDLDFSSEPWPSISESAKDLVRKMLIRD
PKKRLTAHEALCHPWVCVDGVAPDKPLDSAVLSRLKQFSAMNKLKKMALRVIAESLSEEE
IAGLKEMFKMIDTDNSGHITLEELKTGLQRVGANLMDSEINALMEAADIDNSGTIDYGEFI
AATLHINKVEKEDKLFAAFSYFDKDGSGYITQDELQKACEEFGIGDTRLEDIIGDIDQDND
GRIDYNEFVAMMQKGDNPLGRKGYQSNGNFGLGDALKLR
>ZmCPK9

MGNTCVGPSAAGRNGFLANVTLWRPRGDDPAPAPALPPPPSPASDKAPDPVTIPESEHSSH
HSSRSTDLPPAPASQPQDNPTAKKPAPKVKRVQSAGLLADSVLKRDVNTARLKDIYTIGKK
LGQGQFGTTYLCVEKATGREFACKSIAKRKLLTEEDVEDVRREIQIMHHLAGHANVVSIV
GAYEDAVAVQLVMELCAGGELFDRIIQRGHYSEKAAAQLTRVIVGVVEACHSLGVMHRD
LKPENFLFVNQKEDSPLKTIDFGLSIFFKPGEMFTDVVGSPYYVAPEVLLKYYGREVDVWS
AGVIIYILLSGVPPFWDESEQGIFEQVLKGDLDFSSEPWPSISESAKDLVRKMLIRDPKKRLT
AHEALCHPWVCVDGVAPDKPLDSAVLSRLKQFSAMNKLKKMALRVIAESLSEEEIAGLKE
MFKMIDSDNSGHITLEELKTGLQRVGANLMDSEINALMEAADIDNSGTIDYGEFIAATLHI
NKVEKEDKLFAAFSYFDKDGSGYITQDELQKACEEFGLGDTRLEDIIGDIDQDNDGRIDYN
EFVAMMQKGDNPLGRKGHQSNTSFGLGDALKLR
>ZmCPK10
MGNVCVGPRFSKNCGFFGNFSLWPSRSRNSGTPSNPTTTSRSVPVVQVQPSESDAKPQPA
QSTAAPAPVVISEPAQLSQPQPASKPDPSPPLPASQQHAQAQQQQASAPRQQSRKKAAHIK
RISSAGLQVESVLRRKTDNLKDMYSLGRKLGQGQFGTTYLCVDKATGLEYACKSIAKRKL
VTDEDVEDVRREIQIMHHLAGHPNIIAIRGAYEDSVAVHVVMELCAGGELFDRIVRRGHY
TERQAGELARVIVAVVESCHSLGVMHRDLKPENFLFVGNDEESPLKTIDFGLSMFFRPGAC
LSVCPREEFTDVVGSPYYVAPEVLKKRYGQEADVWSAGVIIYILLCGVPPFWAETEQGIFE
QVLHGSLDFDSDPWPSVSGDAKDLLRKVLVRDPKKRLTAHQVLCHPWLQTIASAPDKPL
DSAVLSRLKQFSAMNKLKKMALRVNKLLLSCDTIYRFQGTNMDVSHLQFVAQVIAENLS
EEEIAGLKEMFKMMDTDNSGQINFEELKAGLEKVGANMKESEIYQLMQAADIDNSGTID
YGEFIAATLHLNKIEREDHLFAAFQYFDKDGSGYITADELQQACDEFGIEDIRLEDMIGEVD
QDNDGRIDYNEFVAMMQKPTAGYGKKGHRYNLSIGFRDALKANS
>ZmCPK11
MGNTCVGPSITKNGFFQSVSTVLWKTSQDGDALPAAGSDANGPGRSQSTPPPALPKPASD
VHVAVQSKAPEPVKIAASHSEPAPKAAAPVAANASPSSSPNSSAEALPTRPRPKAPPVKRVS
SAGLLVGSVLKRRTENLKDKYSLGRRLGQGQFGTTYLCVERATGKEFACKSILKRKLVTD
DDVEDVRREIQIMHHLAGHPNVISIRGAYEDAVAVHLVMELCGGGELFDRIVQKGHYTER
KAAELARVIVGVVEACHSMGVMHRDLKPENFLFADQKEEAALKTIDFGLSIFFHPGQIFTD
VVGSPYYVAPEVLKKKYGPEADVWSAGVIIYILLSGVPPFWAENEQGIFEEVLHGRLDFES
EPWPSVSEGAKDLVRRMLIRDPRKRLTAHEVLRHPWVQVGGLAPDKPLDSAVLSRMKQF
SAMNKLKKMALRVIAENLSEDEIAGLKEMFKMIDADNSGQITFEELKVGLKKVGANLQE
SEIYALMQAADVDNNGTIDYGEFIAATLHLNKVEREDHLFAAFQYFDKDGSGYITADELQ
VACEEFGLGDVQLEDVIGEVDQDNDGRIDYNEFVAMMQKPPAGLPNKKAGLQNSFSIGFR
EALRMA
>ZmCPK12
MGNTCVGPSITMNGFFQSVSTALWKTPQDSDALPAAAANGPGGASPGRSQSALPKPASDV
HHHIAVLSEAPEPVKIGAYHSEPAPAVQSEAPEPVKIAASHSEPAPMAAKPGAAAANASPSP
SPRPRPQVKRVSSAGLLVGSVLRRKTENLKDKYSLGRRLGQGQFGTTHLCVERGTGKELA
CKSILKRKLATDDDVEDVRREIQIMHHLAGHPSVVAIRGAYEDAVAVHLVMELCGGGELF
DRIVRRGHYTERKAAELARVIVGVVEACHSMGVMHRDLKPENFLFADHSEEAALKTIDF
GLSIFFRPGQIFTDVVGSPYYVAPEVLKKRYGPEADVWSAGVIIYILLCGVPPFWAENEQGI
FEEVLHGRLDFESEPWPSISDGAKDLVRRMLVRDPRKRLTAHEVLRHPWVQVGGVAPDRP
LDSAVLSRMKQFSAMNKLKKMALRVIAENLSEDEIAGLREMFKMIDADNSGQITFEELKV
GLEKVGANLQESEIYALMQAADVDNNGTIDYGEFIAATLHLNKVEREDHLFAAFQYFDK

DGSGYITADELQVACEEFGLGDVQLEDLIGEVDQDNDGRIDYNEFVAMMQKPTVGVLPK
KADLQNSFSIGFREALRMA
>ZmCPK13
MGNTCVGPSITMNGFFQSVSTALWKTPQEGDALPAAANGPGGPAGAGSQSALPKPASDV
HHVAVQSEAPEPVKIAAYHSEPAPAVRSEAPEPVKIAASHSEPAPMAAKPGGAAANASPSPS
PRPRPQVKRVSSAGLLLGSVLRRKTENLKDKYSLGRRLGQGQFGTTHLCVERATGKELAC
KSILKRKLGSDDDVEDVRREIQIMHHLAGHPSVVGIRGAYEDAVAVHLVMELCGGGELFD
RIVRRGHYTERKAAELARVIVGVVEACHSMGVMHRDLKPENFLFADHSEEAALKTIDFGL
SIFFRPGQIFTDVVGSPYYVAPEVLKKRYGPEADVWSAGVIIYILLCGVPPFWAENEQGIFE
EVLHGRLDFESEPWPSISDGAKDLVRRMLVRDPRKRLTAHEVLRHPWVQVGGVAPDRPL
DSAVLSRMKQFSAMNKLKKMALRVIAENLSEDEIAGLREMFKMIDADNSGQITFEELKVG
LEKVGANLQESEIYALMQAADVDNNGTIDYGEFIAATLHLNKVEREDHLFAAFQYFDKD
GSGYITADELQVACEEFGLGDVQLEDLIGEVDQDNDGRIDYNEFVAMMQKPTVGGSRRRP
ICRTASASGSASGSGRRSGWPRPLCLWLPCCLRVGVDD
>ZmCPK14
MQPDPSGNTNAKAKLPLMAPAPSSGRPASVLPYKTANVRDHYRIGKKLGQGQFGTTYHC
VGKADGAEYACKSIPKRKLLCREDYEDVYREIQIMHHLSEHPNVVRIRGAYEDALFVHIV
MELCAGGELFDRIVAKGHYSERAAANLIKTIVGVVEGCHSLGVMHRDLKPENFLFASTAE
EAPLKATDFGLSMFYKPGDKFSDVVGSPYYVAPEVLQKCYGPEADVWSAGVILYILLCGV
PPFWAETESGIFRQIMRAKLDFESEPWPSISDSAKDLVRNMLTRDPKKRFSAHEVLCHPWI
VDDAVAPDKPIDSAVLSRLKHFSAMNKLKKMALRVIAESLSEEEIGGLKELFKMIDTDNSG
TITFDELKDGLKRVGSELTENEIQALMEAADIDNSGTIDYGEFIAATLHMNKLEREENLVSA
FSFFDKDGSGFITIDELSQACHEFGLDDVHLEDMIKDVDQNNDGQIDYSEFTAMMRKGNA
GATGRRTMRNSLHLNLGELLNPSKT
>ZmCPK15
MQPDPSGNANAKTKLPQLVTAPAPSSGRPASVLPYKTANVRDHYRIGKKLGQGQFGTTYQ
CVGKADGAEYACKSIPKRKLLCREDYEDVYREIQIMHHLSEHPNVVRIRGAYEDALFVHI
VMELCAGGELFDRIVAKGHYSERAAAKLIKTIVGVVEGCHSLGVMHRDLKPENFLFASTA
EEAPLKATDFGLSMFYKPGDKFSDVVGSPYYVAPEVLQKCYGPEADVWSAGVILYILLCG
VPPFWAETEAGIFRQILRGKLDFESEPWPSISDSAKDLVCNMLTRDPKKRFSAHEVLCHAW
IVDDAVAPDKPIDSAVLSRLKHFSAMNKLKKMALRVIAESLSEEEIGGLKELFKMIDTDSSG
TITFDELKDGLKRVGSELTENEIQALMEAADIDNSGTIDYGEFIAATLHMNKLEREENLVSA
FSFFDKDGSGFITIDELSQACREFGLDDLHLEDMIKDVDQNNDGQIDYSEFTAMMRKGNA
GATGRRTMRNSLHLNLGELLNPSKT
>ZmCPK16
MQPDPQGSGRGKAGGANAHARLPPPVTAASAGRPASVLPHKTDNVRDHYRIGKKLGQG
QFGTTYQCVGKADGAEYACKSIPKRKLLCREDYEDVWREIQIMHHLSEHPNVVRIRGAYE
DALFVHLVMELCAGGELFDRIVAKGHYTERAAAQLTRTIVGVVQGCHSLGVMHRDLKPE
NFLFASTAEDAPLKTTDFGLSVFYKPGDKFADVVGSPYYVAPEVLQKCYGPEADVWSAG
VILYILLCGVPPFWAESEAGIFRQILRGKLDLESEPWPSISDSAKDLVRKMLIRDPRKRLTAH
EVLCHPWIVDDAVAPDKPIDSAVLSRLKNFSAMNKLKKMALRVIAESLSEEEIGGLKEMFK
MIDTDNSGTITYDELKDGLKKVGSDLMEPEIQALMDAADIDNSGTIDYGEFLAATLHMNK
LEREESLVSAFAFFDKDGSGFITIDELSQACGQFGLSDVHLEDMIKDVDQNNDGQIDYSEF
AAMMRKGNTGGAGRRTMRNSLHVNLGELLKPAET

>ZmCPK17
MQPDPQGPGRGKAGGANAHPRLPPPVTAGSAGRPASVLPHKTDNVRDHYRIGKKLGQG
QFGTTYQCVGKADGGEYACKSIPKRKLLCREDYEDVWREIQIMHHLSEHPNVVRIRGAYE
DALFVHLVMELCAGGELFDRIVAKGHYTERAAAQLIKTIVGVVQGCHSLGVMHRDLKPE
NFLFASTPEDAPLKATDFGLSVFYKPGDKFSDVVGSPYYVAPEVLQKCYGPESDVWSAGV
ILYILLCGVPPFWAESEAGIFRQILRGKLDLESEPWPSISDSAKDLVRKMLIRDPTKRFTAHE
VLCHPWIVDDAVAPDKPIDSAVLSRLKNFSAMNKLKKMALRVIAESLSEEEIGGLKELFKM
IDTDNSGTITYDELKDGLKRVGSDLMEPEIQALMDAADIDNSGTIDYGEFLAATLHMNKL
EREESLVSAFAFFDKDGSGFITIDELSQACEQFGLSDVHLEDMIKDVDQNNDGQIDYSEFA
AMMRKGNAGGAGRRTMRNSLHVNLGELLKPTET
>ZmCPK18
MGNCCPGSGDAEPAPAASADPSSRLHSGGTSLKAGASVSSAPTPTKPPAPIGPVLGRPMED
VRSIYTVGKELGRGQFGVTSLCTHKATGERFACKTIAKRKLSTKEDVEDVRREVQIMYHL
AGQPNIVELKGAYEDKQSVHLVMELCAGGELFDRIIAKGKYTERAAASLLRTIVEIVHTCH
SMGVIHRDLKPENFLLLSKDDNAPLKATDFGLSVFFKQGEVFKDIVGSAYYIAPEVLKRNY
GPEADIWSIGVIVYILLCGVPPFWAGKGQVDFTSDPWPRISPSAKDLVRKMLTSDPKKRISA
YDVLNHPWIKEDGEAPDTPLDNAVMNRLKQFKAMNQFKKAALRVIAGCLSEEEIRGLKE
MFKSMDADNSGTITVDELRRGLAKQGTKLSEAEVEQLMAAADADGNGTIDYEEFITATM
HMNRMDREEHLYTAFQYFDKDGSGCISKEELEQALKEKGLLDGRDIKDIISEVDADNDGR
IDYSEFVAMMRKGTAEQNPKKRRDVVL
>ZmCPK19
MRPSVSMIYGPGAKIEKLDASFCCFSLLSINKATEGKSKQPLRSRHFLPAFPFMASAPTRPR
HLPGSLRPRLRVRVPGARGPRVSVAPLAVADMGNCCPGSSSSAEPAPPQPPDPSGSSRPVAS
PASVSPTAAPAPARPPAPIGPVLGRPMEDVRATYAVGKELGRGQFGVTSLCTHKATGHRFA
CKTISKRKLSTKEDVEDVRREVQIMYHLSGQPGVVELKGAYEDKGSVHLVMELCAGGEL
FDRIIARGHYTERAAASLLRTIVEIVHTCHSMGVIHRDLKPENFLLLSKDEDAPLKATDFGL
SVFFKEGEVFRDIVGSAYYIAPEVLKRSYGPEADIWSVGVIVYILLSGVPPFWAESEHGIFN
AILRGQVDFTSDPWPRISQGAKDLVRKMLNPDPKQRISAYDVLNHPWIKEDGEAPDTPLD
NAVLGRLKQFRAMNQFKKAALRVIAGCLSEEEIRGLKEMFKSMDSDSSGTITVDELRRGL
ANKGTKLSEAEVQQLMEAADADGNGTIDYEEFITATMHMNRMDRDEHLYTAFQYFDKD
NSGYITMEELEQALREKGLLDGRDIKEIVAEVDADNDGRINYTEFAAMMRKGDPEPSNPK
KRRDIVL
>ZmCPK20
MGQCCSKGAGEAGATEAAPKAQAPSRGASANNADGQRASSSSAVAAAAAAAGGGGGGT
TKPASPTGGARASSGSKPAAAVGTVLGRPMEDVRATYSMGKELGRGQFGVTHLCTHRTS
GEKLACKTIAKRKLAAREDVDDVRREVQIMHHLSGQPNVVGLRGAYEDKQSVHLVMEL
CAGGELFDRIIARGQYTERGAAELLRAIVQIVHTCHSMGVMHRDIKPENFLLLSKDEDAPL
KATDFGLSVFFKEGELLRDIVGSAYYIAPEVLKRKYGPEADIWSVGVMLYIFLAGVPPFWA
ENENGIFTAILRGQLDLSSEPWPHISPGAKDLVKKMLNINPKERLTAFQVLNHPWIKEDGD
APDTPLDNVVLDRLKQFRAMNQFKKAALRIIAGCLSEEEITGLKEMFKNIDKDNSGTITLD
ELKHGLAKHGPKLSDSEMEKLMEAADADGNGLIDYDEFVTATVHMNKLDREEHLYTAF
QYFDKDNSGYITKEELEHALKEQGLYDADKIKDIISDADSDNDGRIDYSEFVAMMRKGTA
GAEPMNIKKRRDIVL
>ZmCPK21

MGQCCSKGQCCSKGAEAAAAAKANAPIRGASASNADGQRPSSSCAAPGGGGGGGGGAT
KPASPTGGARASKPAAAAVGTVLGRPMEDVRATYTMGKELGRGQFGVTHLCTHRASGEK
LACKTIAKRKLAGKEDVEDVRREVQIMHHLSGQPGVVCLRGAYEDRHGVHLVMELCAG
GELFDRIIARGHYTERGAAALLRAIVQTVHTCHSMGVMHRDIKPENFLLLSRDEDAPLKA
TDFGLSVFFKEGEPLRDIVGSAYYIAPEVLRRRYGPEADIWSVGVMLYIFLAGVPPFWAEN
ENGIFTAILRGKLDLSGEPWPHISPGAKDLVKKMLNSNPKERLTAFQVLKLVDHPWIKEDG
DAPDTPLDNVVLDRLKQFRAMNQFKKAALRIIAGCLSEEEITGLKEMFKNIDKDNSGTITL
DELKQGLAKHGPKLSDSEMEQLMEAERNFDSIESEDVANLQTSIPSETFYTQFQADADGN
GLIDYDEFVTATVHMNKLDREEHLYTAFQYFDKDNSGYITKEELEHALKEQGLYDADKIK
EVIADADSDNDGRIDYSEFVAMMRKGTAGAEPMNNKKRRDIVL
>ZmCPK22
MGGRASRHRAQPHDQSSHPPPRPPPKQQQGQQHPNNRPNKPKQQQQPPPPRPPPPHQAPP
AASPAAPGAATVGRVLGRPMEDVRASYTFGRELGRGQFGVTYLATHKPTGRRYACKSIAT
RKLAHSDDVDDVRREVQIMHHLTGHRSIVELRGAYEDRHSVNLVMELCEGGELFDRIIAR
GHYSERAAAALCREIVSVVHSCHSMGVMHRDLKPENFLFLNKREDSPLKATDFGLSVFFK
PGEQFRDLVGSAYYVAPEVLKRRYGAEADIWSAGVILYILLSGVPPFWAENEDGIFDAVLR
GHIDFASDPWPSISNSAKDLVKKMLRQDPKERLTAAEILNHPWIREDGEAPDKPLDITVISR
MKQFRAMNKLKKVALKVVAENLSEEEIVGLKEMFKSLDTDNSGTITLEELRAGLPKLGTK
ISESEIRQLMEAADVDGNGTIDYVEFISATMHMNRLEKEDHIFKAFEYFDKDHSGHITVDE
LEEALKKYDMGDEATVKEIIAEVDTDHDGRINYQEFVAMMKNNSPEIVPNRRRMF
>ZmCPK23
MGNRASRHHRQAAEQQPATPTLPKPQPQIQPPPPPPPTQTQQYQQAPPQSQQKPKPAADV
GAVGRVLGRPMEDVRATYTFGRELGRGQFGVTYLVTHSETGQRFACKSIATRKLVHRDDI
EDVRREVQIMHHLTGHRNIVELRGAYEDRHSVNLVMELCEGGELFDRIIAKGHYTERAAA
SLCREIVAVVHSCHSMGVFHRDLKPENFLFLNNKEDSPLKATDFGLSVFFKHGETFKDLVG
SAYYVAPEVLKRKYGAEADIWSAGVILYILLSGVPPFWAENEDGIFDAVLRGHIDFSSDPW
PSISNGAKDLVKKMLRQDPKERLTAAEILNHPWIREDGEAPDKPLDITVIGRMKQFRAMN
KLKKVALKVVAENLSDEEIMGLKEMFRSLDTDNSGTITLEELRSGLPKLGTKISESEIKQL
MEAADVDGNGTIDYAEFISATMHLNRLEKEDHILKAFEYFDKDHSGYITVDELEEALKKY
DMGDDKTIKEIIAEVDTDHDGRINYQEFVAMMRNNSPEIVPNRRRMF
>ZmCPK24
MEDVKATYTFGRELGRGQFGVTYLVTHRETGQRFACKSIATRKLVHRDDIEDVRREVQIM
HHLTGHRNIVELRGAYEDRRSVNLAMELCEGGELFDRIIARGHYTERAAAALCREIVAVV
HSCHSMGVFHRDLKPENFLFLNNKEDSPLKATDFGLSVFFKPGDTFKDLVGSAYYVAPEV
LKRHYGAEADIWSAGVILYILLSGVPPFWAENEDGIFDAVLHSHIDFSSDPWPSISNGAKDL
VKKMLRQDPKERLTAAEILNHPWIREDGEAPDKPLDITVISRMKQFRAMNKLKKVALKVV
AENLSDEEIMGLKEMFRSLDTDNSGTITLEELRSGLPKLGTKISESEIKQLMEAADVDGNG
TIDYAEFISATMHLNRLEKEDRILKAFEYFDKDHSGYITVDELEEALKKYDMGDDKTIKEII
AEVDTDHDGRINYQEFVAMMRNNTPEIVPNRRRMF
>ZmCPK25
MGNCFTRKYDEHEHEHEIPITTDPPRRTSMPVYRPPRPRDDKTREVPLYSGSDRRPSYRPP
RLPVFPSGPGSTGRRRPPTGQTGTVLDRHTVDVRTLFHLERKLGSGQFGTTYLCTERATG
KKYACKSVSKRKLVRRADIADMRREITILQHLSGQPNVAEFKGAFEDRDDVHVVMELCS
GGELFDRITAKGSYSERQAAAVCRDVLTVVNVCHFMGVMHRDLKPENFLLASPADDAPL

KAIDFGLSVFIEEGKVYKDLVGSAYYVAPEVLRRNYGREIDVWSAGVILYILLCGSPPFWE
ETQKGIFDAILADELDLVSSPWPSISESAKDLIRKMLNRDPQRRITASQALEHPWLKGGAP
DRPIDSAVLSRMKQFKAMNKLKQLALKVIAENLTEDEIKGLKQMFNNMDTDRSGTITVEE
LKDGLAKLGSKISEAEVQKLMEAVDVDKSGSIDYTEFLTAMMNRHKLEKEEDLFLAFQHF
DKDDSGYITRDELEQAMAEYGVGDEASIKEVLDEVDKDKDGRIDYEEFVEMMRKGSYT
>ZmCPK26
MEDVKATYTFGRELGRGQFGVTYLVTHRETGQRFACKSIATRKLVHRDDIEDVRREVQIM
HHLTGHRNIVELRGAYEDRRSVNLAMELCEGGELFDRIIARGHYTERAAAALCREIVAVV
HSCHSMGVFHRDLKPENFLFLNNKEDSPLKATDFGLSVFFKPGDTFKDLVGSAYYVAPEV
LKRHYGAEADIWSAGVILYILLSGVPPFWAENEDGIFDAVLHSHIDFSSDPWPSISNGAKDL
VKKMLRQDPKERLTAAEILNHPWIREDGEAPDKPLDITVISRMKQFRAMNKLKKVALKVV
AENLSDEEIMGLKEMFRSLDTDNSGTITLEELRSGLPKLGTKISESEIKQLMEAADVDGNG
TIDYAEFISATMHLNRLEKEDRILKAFEYFDKDHSGYITVDELEEALKKYDMGDDKTIKEII
AEVDTDHDGRINYQEFVAMMRNNTPEIVPNRRRMF
>ZmCPK27
MVMAILTRQSRRKHLRVYNPPQQAAEVRYTPSATNSSAVPPVAVPPKPTADTILGKQYEDV
RSVYSFGKELGRGQFGVTYLCTEIASGRQYACKSISKRKLVSKADREDIRREIQIMQHLSG
QPNIVEFRGAYEDKSNVHVVMELCAGGELFDRIIAKGHYTERAAATICRAVVNVVNICHF
MGVMHRDLKPENFLLATMEENAMLKATDFGLSVFIEEGKMYRDIVGSAYYVAPEVLRRS
YGKEIDVWSAGVILYILLSGVPPFWAEIEKGIFDAILHEEIDFESQPWPSISESAKDLVRKML
TRDPKKRLTSAQVLQHQWLREGGEASDKPIDSAVLSRMKQFRAMNKLKKMALKVIASNL
NEEEIKGLKQMFMNMDTDNSGTITYEELKAGLAKLGSKLSEAEVKQLMEAADVDGNGSI
DYVEFITATMHRHKLERDEHLFKAFQYFDKDNSGFITRDELESALIEHEMGDTSTIREIISE
VDTDNDGRINYEEFCAMMRGGMQQPMRLK
>ZmCPK28
MGQCCSRAPAPDSGRGGTNGYGYSNQAKPAQTPSSYNPPQQPAEVRYTPPATNPPVVPPV
PVPPKPTADTILGKQYEDVRSVYSLGKELGRGQFGVTYLCTEVASGKQYACKSISKRKLTS
KADREDIRREIQIMQHLSGQPNIVEFRGAYEDKSNVHVVMELCAGGELFDRIIAKGHYTER
AAATICRAVVNVVNICHFMGVMHRDLKPENFLLASKEENAMLKATDFGLSVFIEEGKMY
RDIVGSAYYVAPEVLKRSYGKEIDVWSAGVILYILLSGVPPFWAETEKGIFDAILHEEIDFES
QPWPSISESAKDLVRKMLTRDPKKRLTSAQVLQHSWLREGGGASDKPIDSAVLSRMKQFR
AMNKLKKMALKVIASNLNEEEIKGLKQMFMNMDTDNSGTITYEELKAGLAKLGSKLSE
AEVKQLMEAADVDGNGSIDYVEFITATMHRHKLERDEYLFKAFQYFDKDNSGFITRDELE
SALIEHEMGDTSTIKEIISEVDTDNDGRINYEEFCAMMRGGMQQPMRLK
>ZmCPK29
MGNCCRSPAAAAREDVKSSHFPASAGGKKKPHQQARNGGPGSGEKKRLSVLGEEGCDV
GAGGIEEKYALDRELGRGEFGVTYLCMDRGTRELLACKSISKRKLRTPVDVEDVRREVAI
MRHLPRSPSIVSLREACEDDGAVHLVMELCEGGELFDRIVARGHYTERAAAAVTRTIVEVV
QLCHRHGVMHRDLKPENFLFANKKENSPLKAIDFGLSIFFKPGEKFSEIVGSPYYMAPEVL
KRNYGPEIDIWSAGVILYILLCGVPPFWAETEQGVAQAILRGNIDFKREPWPNVSDNAKDL
VRQMLQPDPKLRLTAKQVLEHPWLQNAKKAPNVPLGDIVKSRLKQFSRMNRFKRKALRV
IADHLSAEEVEDIKDMFKVMDTDNDGIVSYEELKSGIAKFGSHLAESEVQMLIEAVDTNG
RGALDYGEFLAVSLHLQRMANDEHLRRAFLFFDKDGNGFIEPEELQEALMEDGGADTMD
VVNDILQEVDTDKDGKISYEEFVAMMKTGTDWRKASRHYSRGRFNSLSIKLIKDGSVKLG

SE
>ZmCPK30
MGNCCRSPAAAAREDVKSSHFPASAGGKKKPHQARNGGGGGAGEKKRLSVLGEEGCDV
GAGIEEKYALDRELGRGEFGVTYLCMDRGTRELLACKSISKRKLRTPVDVEDVRREVAIM
RHLPKSPTIVSLREACEDDGAVHLVMELCEGGELFDRIVARGHYTERAAAAVTRTILEVVQ
LCHRHGVIHRDLKPENFLFANKKENSPLKAIDFGLSIFFKPGEKFSEIVGSPYYMAPEVLKR
NYGPEIDIWSAGVILYILLCGVPPFWAETEQGVAQAILRGNVDFKREPWPNVSDNAKDLVR
QMLQPDPKLRLTAKQVLEHPWLQNAKKAPNVPLGDIVKSRLKQFSRMNRFKRRALRVIA
DHLSAEEVQDIKEMFKVMDTDNDGIVSYEELKSGIANFGSHLAESEVQMLIEAVDTNGRG
ALDYGEFLAVSLHLQRMANDEHLRRAFLFFDKDGNGFIEPEELQEALMEDGGADTMDVV
NDILQEVDTDKDGKISYDEFVAMMKTGTDWRKASRHYSRGRFNSLSIKLIKDGSVKLGSG
>ZmCPK31
MGNCCRSPAAVAREDVSSSHFPASNAKKKPHQARNGGGAGQKRLSVLGDEGCEVVGIDD
KYVLDRELGRGEFGVTYLCMDRGTKELLACKSISKRKLRTAVDVEDVRREVAIMRHLPKS
PSVVSLREACEDDGAVHLVMELCEGGELFDRIVARGHYTERAAASVTRTILEVVQLCHRH
GVIHRDLKPENFLFANKKENSPLKAIDFGLSIFFKPGEKFSEIVGSPYYMAPEVLKRNYGPE
IDIWSAGVILYILLCGVPPFWAETEQGVAQAILRGNIDFKREPWPNVSENAKDLVRHMLEP
DPKLRLTAKQVLEHHWLQNAKKAPNVPLGDIVKSRLKQFSRMNRFKRRALRVIADHLSA
EEVEDIKDMFKTMDTDNDGIVSYEELKTGIAKLGSHLAESEVQMLIEAVDTNGRGALDYG
EFLAVSLHLQRMANDEHLRRAFLFFDKDGNGFIEPEELREALVDDGAADSMEEVVNDILQ
EVDTDKDGKISYEEFVAMMKTGTDWRKASRHYSRGRFNSLSMKLIKDGSVKLGVE
>ZmCPK32
MGGCYSAFACSRKLRGRISFILPVTERDRDDRSSDGGASSVASHSPSPSPSPRKDDAGPLVV
RTTAAEFARRYVLGRELGRGEFGVTRRCRDAATGEALACKTIRRNRRPRGPAARRLGAGP
PHHGPGAPPAAAGQDPAAAAHAADVQREVAIMRRMSSRGGGAVVRLREACCEDSAGAV
HLVMELCEGGELFDRIVARGHYSERAAAAVFGTIVDVVRLCHSNGVIHRDLKPENFLFAN
KSEDSQLKVIDFGLSVFFSPGDRFTEVVGSAYYMAPEVLKRNYGPEVDVWSAGVILYILLC
GVPPFWGDNDEKIAQAVLRGNIDFNREPWPRVSANAKDLIRRMLDPNPSTRLTARQVLEH
PWLKNADTAPNVSLGEAVRARLQQFSAMNKFKKKALGVVARNLPVEELDKYVQMFHLM
DKDHNGNLTLEELMDGLHINGQPVPESEIRMLLEAADTDGNGTLDCDEFVTVSLHLKKM
SNDEYLASAFRYFDKDGSGFIEPEELRDELGPNDQAILDIIRDVDTDRDGRISYQEFELMM
KSGTDWRNGSRQYSRANFSSLSRRLCKDM
>ZmCPK33

>ZmCPK34
MGNCCATPSTTEESGKSRQKKPKQKANPYNVAYNRGAAPAAARPGLVVLRDPTGRDLGA
QYELGGELGRGEFGVTYLCTESATGARYACKSISKRKLRTPVDVEDVRREVDIMRHMPPH
PNIVSLRAAYEDEDAVHLVMELCEGGELFDRIVARGHYTERAAAAVTRTIVEVVQMCHRH
GVMHRDLKPENFLYASKKESSPLKAIDFGLSVFFRPGERFTEIVGSPYYMAPEVLKRNYGP
EVDVWSAGVILYILLCGVPPFWAETEQGVAQAIIRSVVDFKREPWPRVSEPAKDLVRRMLD
PNPLTRFTAAQVLEHPWLHDSKKMPDISLGDTVRARLQQFAAMNKLKKKALRVIAEHLS
VEEVADIKQMFEKMDVSKNGKLTFEEFKAGLRKLGNQMPDSDLQIMMDAADIDKNGTL
DYEEFVTVSVHVRKIGNDEHIQKAFTYFDRNKSGYIEIEELREALADELEGTDEDIINGIIRD
VDTDKDGKISYDEFAAMMKAGTDWRKASRQYSRQRFSNLSLKLQKDGSLGAETR

>ZmCPK35

>ZmCPK36
MGNCCVTPKAADDAGGGKKPKEPKQKKGKKPNPFSIEYNRSGQASAPKLVVLRDPTGRD
IAARYELGAELGRGEFGVTYLCTDRASGEALACKSISKKKLRTAVDLEDVRREVEIMRHLP
KHPNVVTLRDTYEDDNAVHLVMELCEGGELFDRIVARGHYTERAAALVLRTIVEVVQMC
HKHGVMHRDLKPENFLFANKKESAALKAIDFGLSVFFTPGERFSEIVGSPYYMAPEVLKR
NYGPEVDVWSAGVILYILLCGVPPFWAETEQGVAQAIIRSVIDFKRDPWPRVSDNAKDLVR
GMLNPDPKRRLTAHQVLGHPWLQNIKKAPNVNLGETVKARLQQFSVMNKFKKHALRVI
AEHLSVEEAADIKDMFEKMDLNKDQMLSFEELKLGLHKFGHQMPDADVQTLMEAADA
DGNGSLNYGEFVTLFVHLRKIGNDEHLHKAFAYFDRNQSGYIEIDELRESLADDLGQNRE
EIINAIIRDVDTDKDGKISYDEFATMMKAGTDWRKASRQYSRERFTSLSLKLQKDGSLQM
TSTR
>ZmCPK37
MGNCCVTPNGAADAGGGKKPKEPKQKKGKKPNPFSIEYNRSTPASAPRLVVLREPTGRDI
AERYELGAELGRGEFGVTYLCTDRASGEALACKSISKKKLRTPVDVEDVRREVEIMRHLP
KHPNIVTLRDTYEDDNAVHLVMELCEGGELFDRIVARGHYTERAAALVTRTIVEVVQMCH
KHGVMHRDLKPENFLFANKKESAALKAIDFGLSVFFTPGERFTEIVGSPYYMAPEVLKRN
YGPEVDVWSAGVILYILLCGVPPFWAESEQGVAQAIIRSVIDFKRDPWPRVSDNAKDLVRG
MLNPDPKRRLTAQQVLDHPWLQNIKKAPNVNLGETVKARLQQFSVMNKFKKHALRVIAE
HLSVEEAADIKDMFEKMDLNKDQMLNFDELKLGLHKFGHQIPDADVQILMEAADADGN
GSLDYGEFVTLSVHLRKIGNDEHLHKAFAYFDRNQSGYIEIDELRESLADDLGQNHEEVIN
AIIRDVDTDKDGKISYDEFAAMMKAGTDWRKASRQYSRERFTSLSLKLQKDGSLQMEST
Q
>ZmCPK38
MGLCSSSTAARAASDPGAAAAGDAAAAKKGRGIVACGKRTDFGYDKDFEVRYSLGKLL
GHGQFGYTFAAVDRASTERVAVKRIDKNKMVLPVAVEDVKREVKILKALQGHENVVHFY
NAFEDDNYVYIVMELCEGGELLDRILAKKDSRYSEKDAAVVVRQMLKVAAECHLHGLVH
RDMKPENFLFKSKKEDSPLKATDFGLSDFIKPGRQFRDIVGSAYYVAPEVLKRRSGPESDV
WSIGVITYILLCGRRPFWDKTEDGIFKEVLKKKPDFRRKPWSNITSSAKDFVQKLLVKDPR
ARLTAAQALSHDWVREGGKASEIPLDISVLHNMRQFVKYSRFKQFALRALASTLNSEEMS
DLRDQFNAIDVDKNGTISLEELKQALAKDVPWRLKGPRVLEIIEAIDSNTDGLVDFEEFVA
ATLHVHQLVEHDTEKWKSLSQAAFDKFDVDRDGYITPDELRMHTGMKGSIDPLLEEADI
DKDGKISLDEFRRLLKTASMSARNVQTPRGVRKS
>ZmCPK39
MGACFSSASAAPAGAAVDERRPSKEGDGKKRRRAAGASPDAAAPVRVEFGYERDFEARY
EVGRLLGHGQFGYTFAATDRGSGDRVAVKRIDKAKMTRPVAVEDVKREVKILKALKGHQ
NIVHFYNAFEDDSYVYIVMELCEGGELLDRILAKKNSRYSEKDAAVVVRQMLKVAAECH
LRGLVHRDMKPENFLFKSNKEDSPLKATDFGLSDFIKPGKKFHDIVGSAYYVAPEVLKRRS
GPESDVWSIGVITYILLCGRRPFWDKTEDGIFKEVLRNKPDFRKRPWSSISPGAKDFVKRL
LVKNPRARLTAAQALSHPWVREGGEASDIPVDISVLSNMRQFVKYSRFKQFALRALASTL
NEEELSDLKDQFDAIDIDKSGSISIEEMRHALAKDLPWRLKGPRVLEIIQAIDSNTDGLVDF
KEFVAATLHIHQMAELDSERWGIRCQAAFSKFDLDGDGYITPEELRMVQHTGLKGSIEPLL
EEADIDKDGKISLSEFRKLLRTASMSNVPSPRGPPNPQAL

>ZmCPK40
MGACFSSASAAPAGAAVDERRPSKEGDGKKRRRAAGASPDAAAPVRVEFGYERDFEARY
EVGRLLGHGQFGYTFAATDRGSGDRVAVKRIDKAKMTRPVAVEDVKREVKILKALKGHQ
NIVHFYNAFEDDSYVYIVMELCEGGELLDRILAKKNSRYSEKDAAVVVRQMLKVAAECH
LRGLVHRDMKPENFLFKSNKEDSPLKATDFGLSDFIKPGKKFHDIVGSAYYVAPEVLKRRS
GPESDVWSIGVITYILLCGRRPFWDKTEDGIFKEVLRNKPDFRKRPWSSISPGAKDFVKRL
LVKNPRARLTAAQALSHPWVREGGEASDIPVDISVLSNMRQFVKYSRFKQFALRALASTL
NEEELSDLKDQFDAIDIDKSGSISIEEMRHALAKDLPWRLKGPRVLEIIQAIDSNTDGLVDF
KEFVAATLHIHQMAELDSERWGIRCQAAFSKFDLDGDGYITPEELRMVQHTGLKGSIEPLL
EEADIDKDGKISLSEFRKLLRTASMSNVPSPRGPPNPQAL
>PtCPK1
MGNCNSLASSSSTTTNHRNPPPSNGGIKVLPPNASPPPRSQFLHHSSAAVGRVLGRPMEDV
RNTYTFGREVGRGQFGVTYLVTHKETKQHFACKSIAKRKLINRDDIEDVLREVQIMHHLT
GHRNVVELKGAYEDRHSVNLIMELCEGGELFDRIITKGHYSERAAANLCRQIVTVVHNCH
TMGVIHRDLKPENFLFLSTHEDSPLKATDFGLSVFFKPGDVFKDLVGSAYYVAPEVLRRNY
GAEVDIWSAGVILYILLSGVPPFWGETEQAIFDSILRGHIDFSSDPWPSISSSAKDLVKQMLR
ADPKERISAVEVLNHPWMREDGASDKPLDIAVLTRMKQFRAMNKLKKIALKVIAENLSEE
EIMGLKEMFKSMDTDNNGTITFEELKAGLPKLGTKLSESEVRQLMEAADVDGNGTIDYIE
FITATMHMNRMEREDHLYKAFEYFDKDKSGYITMEELEQALMKYNMGDSKTIKEIIAEVD
TDHDGRINYEEFVAMMRKGNPELASNRRRK
>PtCPK2
MGNCCVTPTGASSSEKKVKGKKKEKKNPFFGDNYAVINESGNVDKLGVLKEPTGRDILA
HYDLGRELGRGEFGVTYLCTDINTGEKFACKSISKKKLRTAVDIEDVRREVEIMKHLPAHP
NIVSLKASYEDDSAVHIVMELCEGGELFDRIVARGHYTERAAAAVMRTIVEVVQMCHKH
GVIHRDLKPENFLFANKKETSALKTIDFGLSVFFKPGERFNEIVGSPYYMAPEVLKRNYGP
EVDVWSAGVILYILLCGVPPFWAETEQGVAQAIIRSVIDFKRDPWPRVSDNAKGLVMKML
NPDPKLRLTAQQVLEHPWIQNAKKAPNVPLGETVKARLKQFSVMNKLKKRALRVIAEHL
SVEEVAGIKDAFDSMDTGKKGSINLEELRVGLQKLGQHIADADLQILMEAADIDGDGALN
YGEFVAISVHIKKMGNDEHLHKAFAFFDRNQSGYIEIEELRESLNDDIDTSSEDVINAIMHD
VDTDKDGRISYEEFATMMKAGTDWRKASRQYSRERFNSLSITLRRDGSLQVAS
>PtCPK3
MGNCCSRGGAQDASTNKEENTEGASPKPAKVAPIGPVLGRPMEDVKSIYSIGKELGRGQF
GITHLCTNKVTGEQFACKTIAKRKLVNKEDVEDVRREVQIMHHLTGQPNIVELKGAYEDK
HSVHLVMELCAGGELFDRIIAKGHYTERAAASLLRTIVQIIHTCHSMGVIHRDLKPENFLLL
NKQENSPLKATDFGLSVFYKSAGEVFKDIVGSAYYIAPEVLKRRYGPEADVWSVGVMLFI
LLSGVPPFWAESEHGIFNAILRGHIDFTSDPWPSISPQAKDLVRKMLTTDPKQRMTAIQVLG
HPWIKEDGEAPDTPLDNAVLSRLKQFKAMNNFKKVALRVIAGCLSEEEIMGLKEMFKGM
DTDNSGTITLEELKQGLAKQGTKLSEYEAKQLMEAADADGNGIIDYDEFITATMHMNRM
DREELLYTAFQHFDKDNSGYITTEELEQALRDFGMHDGRDIKEIISEVDADNDGRINYDEF
VAMMRKGNPEANPKKRRDDVFV
>PtCPK4
MGLCFSRLSFSKFCSHDIHISSTSDEDSDQPKPPKRPAQEHYNPSPPQPPPVATKPAPSSKTS
GKSQTTLTPSSTSNIGSILGKPYVDITKIYDLDKELGRGQFGITYLCTEKATQRKYACKSISR
GKLVTKKDIEDVKMEILILEHLTGQPNIVEFRGAYEDKQNLYLIMELCSGGELFDRIIAKGS

YSEMEAATIIRQIVNVVHVCHFMGVMHRDLKPENFLLASKDDKAPIKATDFGLSVFIEEGK
VYSDIVGSAYYIAPEVLKRSYGKEIDVWSAGIILYILLSGVPPFWAETEKGIFDAVLEGNLD
LQSPPWPNISSSAKDLIRKMLTRDPKRRITAAQALEHPWMKVDGDASDKPIDSAVLIRMK
QFRAMNKLKKLALKVIAENLSEEEIKGLKQMFNNMDTDESGTITYDELKSGLSRLGSKLS
GVEIKQLMDAADVDKSGTIDYLEFITATMHRHRLEMEENLYKAFQYFDKDGSGFISRDEL
RQAMAQYGMGDDATINEVIEDVDTDKDGKINYEEFASMMRKGTQDYEINLI
>PtCPK5
MGNCNSLPSSSSTTTTSTTTDHHNTPPNGVIKVLPPTATPPPRSQHHHHSSASGSTAVGRVL
GRPREDVRNTYIFGRELGRGQFGVTYLVTHKETKQHFACKSIASRKLINRDDIEDVRREVQ
IMHHLTGHRNIVELKGAYEDQHSVNLIMELCEGGELFDRIIAKGHYSERAAANLCRQIVTV
VHNCHTMGVIHRDLKPENFLFLSTHEDSPLKATDFGLSVFFKPGDVFKDLVGSAYYVAPE
VLRRNYGAEVDIWSAGVILYILLSGVPPFWGDTEQVIFDSILRGHIDFSSDPWPSISSSAKDL
VKQMVRADPKERISAVEVLNHPWMREDGASDKPLDIAVLTRMKQFRAMNKLKKVALKV
IAENLSEEEIMGLKEMFKSMDTDNNGTITFEELKAGLPKLGTKLSESEVRQLMEAADVDG
NGTIDYIEFITATMHMNRMEREDHLYKAFEYFDKDKSGYITMEELEQALVKYNMGDTKTI
KEIIAEVDTDHDGRINYEEFVAMMRKGNPELAPTRRRK
>PtCPK6
MGCFSSKEKAPRPDANGCTYRPSTTGYSRQQQPQYHQSQQKAVAPQIQTQSPPRRPQQTQ
QKTPTRPVPKVDTILGKPFEDIKQLYTLSKELGRGQFGITYLCTENATGHSYACKSILRRKL
VNKKDGDDIKREVNIMQHLSGQPNTVEFRGVYEDSQSVHLVMELCAGGELFDRIIAKGH
YSERDAAKIFREIVNVVHACHFMGVMHRDLKPENFLLSSKDEGASLKATDFGLSVFIEEG
KVYRDIVGSAYYVAPEVLRRSYGKEIDIWSAGVILYILLSGVPPFWAENERGIFDAILQGDI
DFESQPWPSITNSAKDLVRRMLTQDPNKRITSAQVLEHPWIKDGGADRPIDSAVLSRMKQF
RAMNKLMKLALKVIAENLSEEEIKGLKAMFTNMDTDKSGTITYEELKTGLARLGSKLSEA
EVKNLMEAADVDGNGSIDYIEFISATMHRYKLERDEHLYKAFQYFDKDSSGYITRDELEL
AMKEYGMGDESSIKEIIAEVDADNDGRINYEEFCAMMRSGTPHAPSLY
>PtCPK7
MGNTCRGSFKGKLYQGYNQPDEQSTTTASNSKRNASSDHSNSEYSISSLTPQELNPKKDSN
LPLISPTKKDTIMRRGVDNQAYYVLGHKTANIRDLYTLGRKLGQGQFGTTYLCTDISTGIE
YACKSISKRKLISKEDVEDVRREIQIMHHLAGHKNIVTIKGAYEDQLYVHIVMELCSGGEL
FDRIIQRGHYTERKAAELTKIIVGVVEACHSLGVMHRDLKPENFLLVNKDDDFSLKAIDFG
LSVFFKPGQVFTDVVGSPYYVAPEVLLKQYGPEADVWTAGVILYILLSGVPPFWAETQQGI
FDAVLKGYIDFDSDPWPVISDSAKDLIRKMLCSQPSERLTAHEVLCHPWINDNGVAPDRAL
DPAVLSRLKQFSAMNKLKKMALRVIAESLSEEEIAGLKEMFKAMDTDNSGAITFDELKAG
LRRYGSTLKDVEIRDLMDAADVDNSGTIDYGEFVAATVHLNKLEREEHLVAAFQYFDKD
GSGYITVDELQQACAEHNMTDVLLEDIIKEVDQDNDGRIDYGEFVAMMQKGNAGIGRRT
MRNSLNMSMRDAPGAL
>PtCPK8
MGACFSTIKVSGSNSNNNVIHSRKEPTKPQTKTTKATTATRKKQEVVHHHQNNKNVNNE
AEKKLKVKEKQSSKAIPCGKRTDFGYDKDFDMRYTIGKLLGHGQFGYTYVAIDKANGDR
VAVKRIEKNKMVLPIAVEDVKREVKILRELTGHENVVQFYNALEDDSYVYIVMELCEGGE
LLDRILSKKDSRYTEKDAAVVVRQMLKVAAECHLHGLVHRDMKPENFLFKSTKGDSPLK
ATDFGLSDFIKPGRKFQDIVGSAYYVAPEVLKRKSGPESDVWSIGVITYILLCGRRPFWDKT
EDGIFKEVLKNKPDFRRKPWPTISTSAKDFVNKLLVKDPRARLTAAQALSHPWVREGGVA

SEIPIDISVLSNMRQFVKYSRLKQFALRALASTIDEEELADLKDQFDAIDVDKNGAISLEEM
RQALAKDLPWKLKESRVLEIVQAIDSNTDGLVDFTEFVAAALHVHQLQEHNSEKWQLRS
QAAFEKFDIDRDGYITPEELRMHTGLRGSIDPLLEEADIDKDGKISLSEFRRLLRTASMSSR
NVPSPSGHRKSHKI

>PtCPK9

MGKCFSRSCSHDIPISSSSDDAYYQPTPPKRPPLPPTLTTPSSKTSGTSQTIVTSSSNIGPILGK
PYVEITTIYDLDKELGRGQFGITYLCTEKASGRMYACKSISRRKLVKVKDIEDVKREILILQ
HLTGQPNIVEFKGAYEDKQNLHLIMELCSGGELFDRILAKGSYSESEAATIIRQIVNVVHAC
HFMGVMHRDLKPENFLLASKDPNALIKATDFGLSVFIEEGKVYNDIVGSSYYVAPEVLKR
SYGKEIDVWSAGIILYILLSGVPPFWAEAEKGIYEAILNGNLDLQSKPWPKISPSAKDLIKK
MLTRDPKSRITAAQALDHPWMKVGGEASNTLIDSVVLIRMKQFRAMNKLKKLALKVIAE
NLSEEEIKGLRQMFNNMDTDRSGTITYEELKSGLLRLGSKLTEVEIKQLMDGADVDNNGT
IDYVEFITATMHRHRLEKEENLYKAFQYFDKDNSGFITRDELRQAMSQYGMGDEATIDEVI
EDVDTDKDGNINYEEFATMMRK

>PtCPK10

MGNCCVTPSGNPEKTKHKKKHNRYALDYGLHDGGHKLIVLKDPTGKELEQRYELGTELG
RGEFGITYLCTDKDTGENFACKCISKKKLKTDIDIEDVRREVEIMKRMPQHPNLVTLKDTY
EDDNAVHLVMELCEGGELFDRIVARGHYTERAAAGVTKTIVEVVQICHEHGVMHRDLKP
ENFLFGNKKENAPLKAIDFGLSVFFKPGERFTEIVGSPYYMAPEVLKRNYGQEVDVWSAG
VILYILLCGVPPFWAETEQGVAQAIIRSVVDFKRDPWPKVSDNAKDLVRKMLDPDPKCRLT
AQQVLDHPWLQNAKKAPNVSLGETVRSKLKQFSIMNKLKKRAMRVIAEHLSVEEAAGIK
EGFQLMDTGNKGKINIDELRVGLQKLGQQVLESDLQILMEVGDTDRDGYLDYGEFVAITV
HLKKMGNDEHLRQAFKFFDQNQSGYIEIDELRGALADEVDGSNEEVINAIINDVDTDKDG
KISYEEFTTMMKAGTDWRKASRQYSRERFNSLSLKLMRDGSLKLANEGR

>PtCPK11

MGNTCVGPNLGNNGFFNTVTAAIWRSRPPEDRLHLPKGEDSSKNNGDSEAGPVGGSKKS
EGSKKGSNDHPPILVQNTPPEPVKMPNEAPPPKVIEHEKSIKQEMREVGIGQPGEEQKGKK
PTHVKRVSSVALQMESVLGRKTGNLKDIYSLGRKLGQGQFGTTFLCIEKATGKEFACKSIA
KRKLTTQEDVDDVRREIQIMHHLEGHPNVIKIVDAYEDAVAVHVVMELCSGGELFDRIVR
RGHYTEKQAAELARLIVGVVEACHSLGVMHRDLKPENFLFVSQEEESPLQTIDFGLSVFF
RPGETFTDIVGSPYYVAPEVLRKLYGPKCDVWSAGVIIYILLSGVPPFWDETEQGIFEQVLK
GELDFESEPWPNISESAKDLVRKMLVRDPKRRLTAHEVLCHPWVQMEGVAPDRPLDSAVL
SRLMQFSAMNRLKKIVIRVIAESLSEEEIAGLKEMFKMIDADNSGHITLEELKTGLEKVGA
NTKDSEIAGLMQAADVDNSGTIDYGEFVAAMLHLNKIEKEDHLYAAFSYFDQDGSGYITK
DELQQACEKFGLGDVQLDEIIREVDQDDDGRIDYSEFVAMMQDTGFGQTRSQIT

>PtCPK12

MGNNNCIGSRVSRDGIFQTISSSVWWARSKDCLITYNKKENVDGLSLNRVQEPPFHAQNK
PPEQMKIAKEEIINQVPSPPKPKENATVASEIIMEVEESRPAKPASDKEVKKPAEPTRPSKPL
VTRTPSAGLQVDSVLKTRTGHLKDYYNLGRKLGRGQFGTTFLCVEKATGKEYACKSIAKR
NLLTADDVEDVRREIQIMHHLAGHPNVISINGAYEDAVAVHVVMELCAGGELFDRIIKRGH
YTERKAAQLTRTIVGVIEACHSLGVMHRDLKPENFLFVNEREDSPLKAIDFGLSVFFKPGEI
LNDVVGSPYYVAPEVLRKRYGPEADVWSAGVMVYILLCGVPPFWAEKEHDIFEEVLHGH
LDFTSNPWPKVSASAKDLIRRMLVRDPKKRLTAHEVLCHPWVRDDGVAPDKPLDPAVLSR
LKQFSAMNKIKKMALRIIAENLSEEEIAGLKEIFKMIDTDNSGQITFEELKVGLRRFGANLT

EAEIYSLLRAADVDNSGTIDYKEFIAATLHLHKVEKEDHLFAAFSYFDKDDSGYITIDELQ
QACNEFGMDDVHLEEMIREVDQDNDGRIDYNEFVAMMQRGNTELVKNG
>PtCPK13
MGSCVSTPANLGQIISKRSSHNVRKPLPSPDQNDSVVQTPRALQIASVIKEPTGHNIHEKYT
FGKELGRGEFGITYHCFDIKTGEKYACKTISKSKLKSEIDVEDVRREVEIMRHLPKHPNIVS
FREAYEDRDAVHLVMELCEGGELFDRIISKGHYSERAAAMVTKTILEIVKVCHDHGVIHR
DLKPENFLFADASESSQLKAIDFGLSIFFEPGQRFREIVGSPYYMAPEILRRNYGPEVDVWS
TGVILYILLCGVPPFWAETEEGIAHAIVRGEIDFARDPWPKVSEEGKDIVKKMLDQNPYNR
LTVEEVLENPWIQNASDVPNISLGENVRTKIKQFSLMNRFKKKALRVVADSLPDEQVDKIK
QMFHMMDTDHNGDLSFQELKDGLNKFGHSVPDPDVKLLMDAADADGNGSLSCEEFVAV
SVHLKRISSDKHLTQAFRFFDKDQNGFIEFDELREAMSNDDLGPNNEQVIKDIIFDVDLDK
DGRISYNEFKAMMKSGLDWKMGSRQYSRAMMKALSINLLKNESKQLK
>PtCPK14
MGACFSTINISGSNSNNNTKANHNRKEPTKPQTRTTKAATRKKQEVVHHHHQINKNVNN
EAEKKLKVKEKQSSKAIPCGKRTDFGYDKDFDIRYTIGKLLGHGQFGYTYVATDKGNGDR
VAVKRIDKNKMVLPIAVEDVKREVRILQELTGHENVVQFHNAFEDDSYVYIVMELCEGGE
LLDRILAKKDSRYTEKDAAVVVRQMLKVAAECHLHGLVHRDMKPENFLFKSTKEDSPLK
ATDFGLSDFIKPGRKFKDIVGSAYYVAPEVLKRNSGPESDVWSIGVITYILLCGRRPFWDKT
EDGIFKEVLRNKPDFRRKPWPTISTSAKDFVQKLLVKDPRARLTAAQALSHPWVREGGDA
SEIPIDISVLSNMRQFVKYSRLKQFALRALASTIDEEELADLKDQFDAIDVDKNGAISLEEM
RQALAKDLPWKLKESLVLEIVQAIDSNTDGLVDFTEFVAAALHVHQLEEHNSEKWQLRSQ
AAFEKFDIDRDGYITPEELRMHSGLRGSVDPLLEEADIDKDGRISLSEFRRLLRTASMSSQN
VPDPSGHRNSKKL
>PtCPK15
MGNTCVGPSISKNGFFQSVSAAMWRTRSPDDSISQTNGESVHELEAVSRESEPPLPVQSKP
PEQMTIPKPEAPEKSEKSDEPAKPKKPPQVKRVSSAGLRTESVLKTKAGNLKEFFSLGKKL
GQGQFGTTFLCVEKATKKEFACKSIAKRKLLTDEDVEDVRREISIMHHLAGHPNVISIKGA
YEDAVAVHVVMEICAGGELFDRIIKRGHYTERKAAELTRTIVGVVEACHSLGVMHRDLKP
ENFLFVNQKEDSLLKTIDFGLSIFFKPGERFHDVVGSPYYVAPEVLKKRYGPEADVWSAG
VIIYILLSGVPPFWAETEQEIFEQVLHGDLDFSSDPWPSISESAKDLVRRMLVRDPRRRLTAH
EVLCHPWVHEDGVAPDKPLDSAVLSRLKQFSAMNKLKKMALRIIAETLSEEEIAGLKEMF
KMIDSDGSGQITFEELKAGLKRVGANLKESEIYDLMQAADVDNSGTIDYGEFIAATLHLN
KIERQDHLFAAFSYFDKDGSGYITPDELQQACEEFGIGDVRLEEMIKEVDQDNDGRIDYNE
FVAMMQKGNVAGPARKGLEHSFSINFREALKL
>PtCPK16
MGNCCFTPSGASSSEKKVKGKKKKQNPFFGDSYAVTNGSGDVDKLWVLKEPTGRDILAH
YDLGRELGRGEFGITYLCTDVNSGDKFACKSISKKKLRTAVDIDDVRREVEIMKHLPAHPN
IVTLKASYEDDTAVHIVMELCEGGELFDRIVARGHYTERAAAAVMRTIVEVVQMCHKHG
VMHRDLKPENFLFANKKETAVLKAIDFGLSVFFKPGERFNEIVGSPYYMAPEILKRNYGPE
VDVWSAGVILYILLCGVPPFWAETEQGVAQAIIRSFIDFKRDPWPRVSDNAKDLVKKMLNP
DPKLRLTAQQVLEHPWIQNAKKAPNVPLGETVRARLKQFSVMNKLKKRALRVIAEHLSV
EEVAGIKEAFDMMDTGKRGSINLEELRVGLQKLGQNIADADLRILMEAADVDGDGALNY
GEFVAISVHIKKMGNDEHLHKAFAFFDRNQSGYIEIEELRESLNDDVDTNNEDVINAIMHD
VDTDKDGRISYEEFATMMKAGTDWRKASRQYSRERFNNLSITLRRDGSLQAAT

>PtCPK17
MGNCCSRGGAQDASANKDDTTEGTNRTNGQLDAPSTTPPSPPPGASPKPAKVTPIGPVLG
RPMEDAKSIYTIGKELGRGQFGITHLCTNKVTGEQFACKTIAKRKLVNKEDVEDVRREVQI
MHHLTGQPNIVELKGAYEDKHSVHLVMELCAGGELFDRIIAKGHYTERAAASLLRTIVQIV
HTCHSMGVIHRDLKPENFLLLNKHENSPLKATDFGLSVFYKSGEVFKDIVGSAYYIAPEVL
KRRYGPEADIWSVGVMLYILLSGVPPFWAESEHGIFNAILRCHIDFTSDPWPSISPQAKDLV
RKMLASDPKQRMTAIQVLSHPWIKEDGEAPDTPLDNAVLSRLKQFKAMNNFKKVALRVI
AGCLSEEEIMGLKEMFKGMDTDNSGTITLEELKQGLAKQGTKLSEYEVKQLMEAADADG
NGTIDYDEFITATMHMNRMDREEHLYTAFQHFDKDNSGYITTEELEQALREFGMHDGRDI
KEIISEVDADNDGRINYDEFVAMMRKGNPEANPKKRRNDVFV
>PtCPK18
MGNTCVGPSISRNGFFQSVSAAMWRNRSPDDSMSQTNGESVHEHEAASRELESPLPVQS
KPPEQMTIPKPEEPEKPVKSEEPAKPKKPLQVKRVSSAGLRTEYVLTTQAGNLKEFYSLGK
KLGQGQFGTTFLCVEKATKKEFACKSIAKRKLLTDEDVEDVRREIQIMHHLAGHPNVISIK
GAYEDAMAVHVVMELCAGGELFDRIIQRGHYTERKAAELTRTIVGVVEACHSLGVMHRD
LKPENFLFVNEKEDSLLKTIDFGLSIFFKPGERFSDVVGSPYYVAPEVLKKRYGPEADVWS
AGVIVYILLSGVPPFWAENEEGIFEQVLHGDLDFSSDPWPSISESAKDLVRRMLIRDPRRRL
TAHEVLCHPWVQEDGVAPDKPLDSAVLSRLKQFSAMNKFKKMALRVIAETLSEEEIAGLK
EMFKMIDTDGSGHITFEELKAGLKRFGANLKESEIYDLMQAADVDNSGTIDYGEFIAATL
HLNKIERDDHLFAAFSYFDKDGSGYITPDELQKACEEFGWEDVRLEEMIREVDQDNDGRI
DYNEFVAMMQKGNVASPARKGLEHSFSINFREALKL
>PtCPK19
MGNCNTCVRPDTSPEDTETHQTKTKKPKKSNPYSEDFPHHQTTTNNRSSPAPIRVLKDSSV
PLSHRPRISDKYILGRELGRGEFGITYLCTDRENKEALACKSISKRKLRTAVDIEDVRREVAI
MSTLPEHPNIVKLKATYEDYENVHLVMELCEGGELFDRIVARGHYSERAAAHVARTVAEV
VRMCHANGVMHRDLKPENFLFANKKENSALKAIDFGLSVFFKPEERFSEIVGSPYYMAPE
VLKRNYGPEVDVWSAGVILYILLCGVPPFWAETEQGVALAILRGVIDFKREPWPQISESAK
SLVRQMLEPDPRKRLNAQQVLEHPWLQNAKKAPNVPLGDIVRSRLKQFSVMNRFKKKA
LRVIAEHLTVEEVEVIRDMFALMDTDNDGKVTYEELRTGLRKVGSQLAEPEIKMLMEVAD
VDGNGVLDYGEFVAVTIHLQKMENDEHFRRAFMFFDTDGSGYIELDELRGALADEYGET
DNDVLNDIMREVDTDKDGCISYEEFVAMMKAGTDWRKASRQYSRERFKSLSLNLMKDG
SLHLHDAFTGQSVAV
>PtCPK20
MNEETTRPPPPSTAEPAPAPRARPTIRKPTTSVLPHQTPRLRDHYLLGKKLGQGQFGTTYL
CTHKASNNLYACKSIPKRKLLCKEDYEDVYREIQIMHHLSGQPNVVQIKDTYEDPMFVHL
VMELCEGGELFDRIVERGQYSEKEAANLIKNIIGVVEYCHSLGVMHRDLKPENFLFDKPG
DDAKLKTTDFGLSVFYKPGQYFYDVVGSPYYVAPEVLLKYYGPQADVWSAGVILYILLS
GVPPFWAETESGIFRQILQGKLDLESDPWPNISESAKDLVRKMLERDPRQRITAHEVLCNP
WIVDDRVAPDKPLDSAVLSRLKQFSAMNKLKKMALRVIAERLSEEEIGGLKELFKMIDTD
NSGTITFEELKHGLKRVGSQMTEAEIKTLMDAADIDNSGTIDYGEFLAATLHLNKMDRED
NLVAAFSYFDKDGSGYITIDELQQACKDFGLGDVHLDETIKEIDLDNDGRIDYGEFAAMM
RKGDGGVGRTRTMRNNLNFNLADAFGVDALGMKDATSDAD
>PtCPK21
MGNCNTCVRPDTTTEDTNEHQTRNKKSKNHKKSNPYSEDYPHHQTTTNNRSSPAPIRVLK

DSSVSLSQRPRISDKYILGRELGRGEFGITFLCTDRETKESLACKSISKRKLRTAVDIEDVRR
EVAIMSTLPEHPNIVKLRATYEDFENVHLVMELCEGGELFDRIVARGHYSERAAAHVARTI
AEVVRMCHANGVMHRDLKPENFLFANKKENSVLKAIDFGLSVLFKPGERFSEIVGSPYY
MAPEVLRRNYGPEVDVWSAGVILYILLCGVPPFWAGMSVKTEQGVALAILRGVIDFKREP
WPQISENAKSLVRQMLEPDPSKRLNAQQVLEHPWLQNAKKAPNVPLGDIVRARLKQFSV
MNRFKKRALRVIAEHLSVEEVEVIRDMFALMDTDNDGKVTYEELRTGLRKVGSQLAEPEI
KMLMEVADVDGNGVLDYGEFVAVTIHLQKMENDEHFRRAFMFFDKDGNGYIELDELRE
GLADEYGETDDDVLNDIMREVDTDKDGCISYEEFVAMMKAGTDWRKASRQYSRERFKS
LSLNLMKDGSLHLHDALTGQSVAV
>PtCPK22
MGNCCVTPPGVPDHEKKKHKKKQNPFALDFGHHNRGTNHKLIVLRDPTGKEIEQRYELG
RELGRGEFGITYLCTDKETGENFACKSISKKKLRTAVDIEDVRREVEIMKQMPQHPNLVTL
KDTYEDDSAVHLVMELCEGGELFDRIVARGHYTERAAAAVTKTIVEVVQICHEHGVMHR
DLKPENFLFGNKKENAPLKAIDFGLSVFFKPGERFTEIVGSPYYMAPEVLKRNYGPEVDV
WSAGVILYILLCGVPPFWAETEQGVAQAIIRSVIDFKRDPWPKVSENAKDLVRKMLDPDPK
RRLTAQQVLDHPWLQNAKKAPNVSLGETVRTRLKQFSVMNKLKKRALRVIAEHLSVEEV
AGIKEGFQLMDTGNKGKINIDELRVGLQKLGQQVPEIDLQILMEVGDADRDGYLDYGEFV
AITVHLRKMGNDEHLRKAFEFFDQNQSGHIEIDELRDALADEVDGSNEDVINAIIHDVDT
DKDGKISYEEFAAMMKAGTDWRKASRQYSRERFNNLSLKLMKDGSLKLTSEGR
>PtCPK23
MGNTCVGPNLGNKGFLNSVTAAIWRSRPPEDRLPPPKGGDGSNNNGDSNAGLIGGSKKS
EGSRKGSTDHPSMPVQNTPPEPVKMLNEALPPPKFIEHEKSIKPEMRDVGIGKPGEEQKGK
KPTHVKRVSSIALQMESVLGRKTGNLKDIYSLGRKLGQGQFGTTFLCVEKATGKEFACKTI
AKRKLTTPEDVEDVRREIQIMHHLEGHPNVIRIVDAYEDAVAVHVVMELCSGGELFDRIVQ
RGHYTEKKAAELARLIVGVVEACHSLGVMHRDLKPENFLFVSQEEDSPLKTIDFGLSVFF
RPGETLTDVVGSPYYVAPDVLRKLYGPKCDVWSAGVIIYILLSGVPPFWDESEQGIFEQVL
KGELDFESEPWPNISESAKDLVRKMLVRDPKKRLTAHEVLCHPWVKMEGVALDRPLDPAV
LSRLKKFSAMNKLKKIAIRVIAESLSEEEIAGLKEMFKMIDTDSSGHITLEELKTGLERVGA
NIKDSELAGLMQAADVDNSGTIDYGEFIAAMLHLNKIVKEDHLYSAFSYFDKDGSGYITQ
DELQQACEQFGLGDVQLEEIIREVDQDNDGRIDYSEFVAMMQDTGFSQTRSQIT
>PtCPK24
MGNSNCIGSRFSKDGLFQTISSSIRWSRSTDGSIIHSKRENGEGLSLTKVQELPVHAQRKPP
EQMKIVKEETKQVTLPASPKEGAAKPSEIVMKVKEESKPAQPASDKEEKKPAVPTGPNKPL
VKRTPSAGLQVDSVLKTRTGHLREYYNLGRKLGHGQFGTIFLCAEKATGKEYACKSISKR
KLLTSDDVVDVRREIQIMHHLAGHPNVVSIKGAYEDEVAVHVVMELCAGGELFDRIIKRG
HYTERKAAQLTRTIVGVIEACHSLGVMHRDLKPENFLFVNESEDSPLKAIDFGLSVFFKPG
EIFNDVVGSPYYVAPEVLRKRYGPEADVWSAGVIVYILLCGVPPFWAEKEHDIFEEVLHG
DLDFTSDPWPNISASAKDLVRRMLVRDPKKRLTAHEVLCHPWVHDDGVAPDKPLDPAVLS
SLKQFSAMNKIKKMALRIIAENVSEEEIAGLKEIFKMIDTDNSGQITFEELKVGLRRFGANL
SEAEIYSLLRAADVDNSGTIDYKEFIAATLHLNKVEREDRLFAAFSYFDKDNSGYITIDELQ
QACNEFGMDDVHLEEMIREVDQDKDGRIDFNEFVAMMQKGNAELGKNGLQGNNFGIGF
REALSVY
>PtCPK25
MGNCCRSPAAVAREDVKSSFSGQDHGKKNSTAKKTPQPIRVLTGVPKENIEERYLVDRELG

RGEFGVTYLCIERDSRELLACKSISKRKLRTAVDIEDVRREVAIMKHLPKNSSIVSLKEACE
DDNAVHLVMELCEGGELFDRIVARGHYTERAAAAVTRTIVEVVQLCHKHGVIHRDLKPEN
FLFANKKENSPLKAIDFGLSIFFKPGERFSEIVGSPYYMAPEVLKRNYGPEIDIWSAGVILYI
LLCGVPPFWAESEQGVAQAILRGIIDFKRDPWPNISESAKSLVRQMLEPDPKLRLTARQVIE
HPWLQNAKKAPNVPLGDVVKSRLKQFSMMNRFKRKALRVIADFLSIEEVEDIKEMFMKM
DTDGDGIVSVEELKTGLRNFGSQLAESEVQMLIEAVDTNEKGKLDYGEFVAVSLHLQRMA
NDEHIHKAFSYFDKDGNGYIEPDELRDALMEDGADDCTDVANDIFQEVDTDKDGRISYDE
FVAMMKTGTDWRKASRHYSRGRFNSLSMKLMKDGSLNLGSE
>PtCPK26
MDDQPKPSSSSAPPLSSRPKWVLPYKTQNLRDHYSIGRKLGQGQFGTTFLCTHKTSGKKY
ACKSIPKRKLLCKEDYEDVWREIQIMHHLSEHPHVVRISGAYEDISCVHLVMELCEGGELF
DRIVKKGHYSEKEAAKLMKTIVGVVEACHSLGVMHRDLKPENFLLHSVEEDAPLKATDF
GLSVFYKPGETFCDVVGSPYYVAPEVLRKHYGPEADVWSAGIILYILLSGVPPFWAETEIGI
FKQILQGKLDFESEPWPSISDSAKDLIRKMLERNPKKRLTAHEVLCHPWIVDDRIAPDKPL
DSAVLSRLKQFSAMNKLKKMALRVIADRLSEEEIGGLKELFKMIDTDNSGTITFDELKDGL
RRVGSELMESEIKDLMDAADIDNSGTIDYGEFLAATVHLNKLEREENLVSAFSFFDKDSSG
YITIDELQQACKEFGLSELHLDEMIKEIDQDNDGQIDYGEFAAMMRKGNGGIGRRTMRST
FNLGDALGLTTNGSKTSD
>PtCPK27
MNEETSRAPPPQQRPSSAAPPASRARPTTRKPATSVLPHQTPRLRDHYLFGKKLGQGQFGI
TYLCTHKASSALYACKSISKRKLLCREDYEDVYREIQIMHHLSGQPNVVQIKDTYEDSMF
VHLVMELCAGGELFDRIVAKGHYSEKEAAKLTKNIIGVVEYCHCLGVMHRDLKPENFLFD
KPGDDAKLKTTDFGLSVFYKPGQYFYDVVGSPYYVAPEVLLKHYGPQADVWSAGVILYI
LLSGVPPFWAETDSGIFRQILQGKLDLESDPWPNISESAKDLIRKMLDRDPKQRITAHEVLC
NPWIVDDRVAPDKPLDSAVLSRLKHFSAMNKLKKMALRVIAERLSEEEIGGLKELFKMIDT
DNSGTITFEELKHGLKRVGSQMTETEIKDLMDAADIDNSGTIDYGEFLAATLHLNKMERE
DNLVAAFSYFDKDGSGYITIDELQQACKDFGLGDVHLDETIKEIDQDNDGRIDYGEFAAM
MRKGDGGVGRTRTMRSNLNFNLADALGVENATSDAK
>PtCPK28
MGCCGSKGNAPTPDVNGYRGPATGYPRQTNQQQQPQYHPSQQKVTVPQIQTQTPPTRPQ
QTQQQTPTRPAPDTILGKPFEDIKQHYTLGKELGRGQFGVTYLCTENSTSHTYACKSILKR
KLVNKNDKEDMKREVHIMQDLSGQPNIVEFRGAYEDRQSVHLVMELCAGGELFDRIIAK
GHYSERDAAKICREIVNVVHACHFMGVMHRDLKPENFLLSSKAEGAKLKATDFGLSVFIE
EGKVYRDIVGSAYYVAPEVLRRSYGKEIDIWSAGVILYILLSGVPPFWAENEKGIFDAILQG
DIDFESDPWPSISNSAKDLVRRMLTQDPKKRITSTQVLEHPWIKEGGADKPLDSAVLSRMK
QFRAMNKLKKLALKVIAENLSEEEIKGLKTMFTNMDTDKSGTITYEELKTGLARLGSKLS
EAEVKQLMEAADVDGNGSIDYIEFISATMHRYKLERDEHLYKAFQYFDKDSSGYITRDEL
ESAMKEYGMGDEATIKEIIAEVDADNDGKINYEEFCAMMRSGTQHAGKLF
