# Supplementary figures and images for: Genome-wide Identification and Expression Analysis of the CDPK Gene Family in Grape, Vitis spp
Source: BMC Plant Biol. 2015 Jun 30;15:164. doi: 10.1186/s12870-015-0552-z (PMC4485369; doi:10.1186/s12870-015-0552-z)

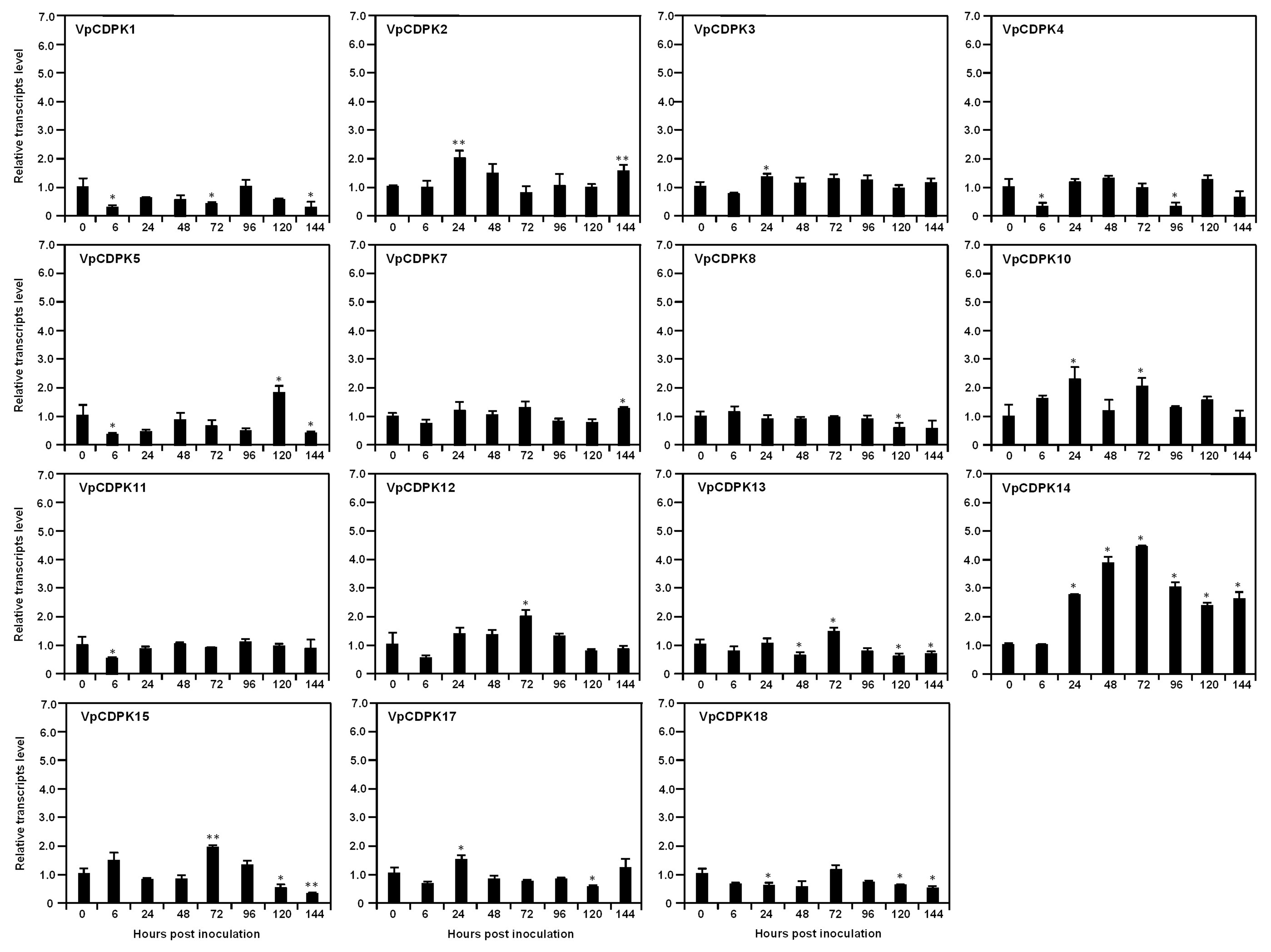

Supplement: Additional file 3: — Detailed expression profiling of the remaining 15 VpCDPK genes during powdery mildew infection. Detailed expression levels were measured by RT-qPCR. Grape Actin1 (GenBank Accession number AY680701) was used as an internal control. The experiments were repeated three times and gave consistent results. Mean values and SDs were obtained from three biological and three technical replicates. The data were showed as mean value ± SD. * and ** represent statistically significant (p<0.05) or highly significant (p<0.01), respectively. [file 12870_2015_552_MOESM3_ESM.tiff]

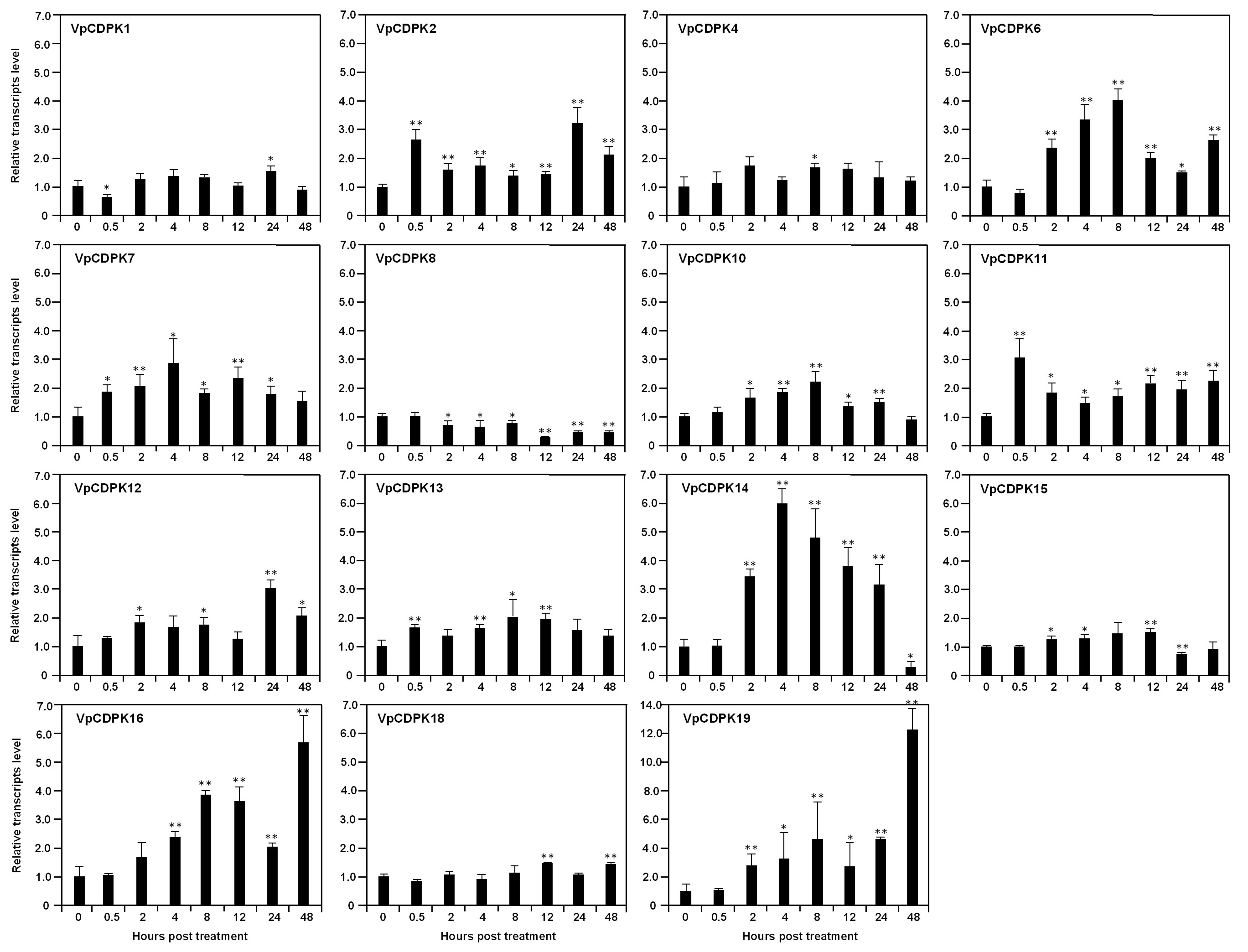

Supplement: Additional file 4: — Detailed expression profiling of the remaining 15 VpCDPK genes under NaCl treatment. Detailed expression levels were measured by RT-qPCR. Grape Actin1 (GenBank Accession number AY680701) was used as an internal control. The experiments were repeated three times and gave consistent results. Mean values and SDs were obtained from three biological and three technical replicates. The data were showed as mean value ± SD. * and ** represent statistically significant (p<0.05) or highly significant (p<0.01), respectively. [file 12870_2015_552_MOESM4_ESM.tiff]

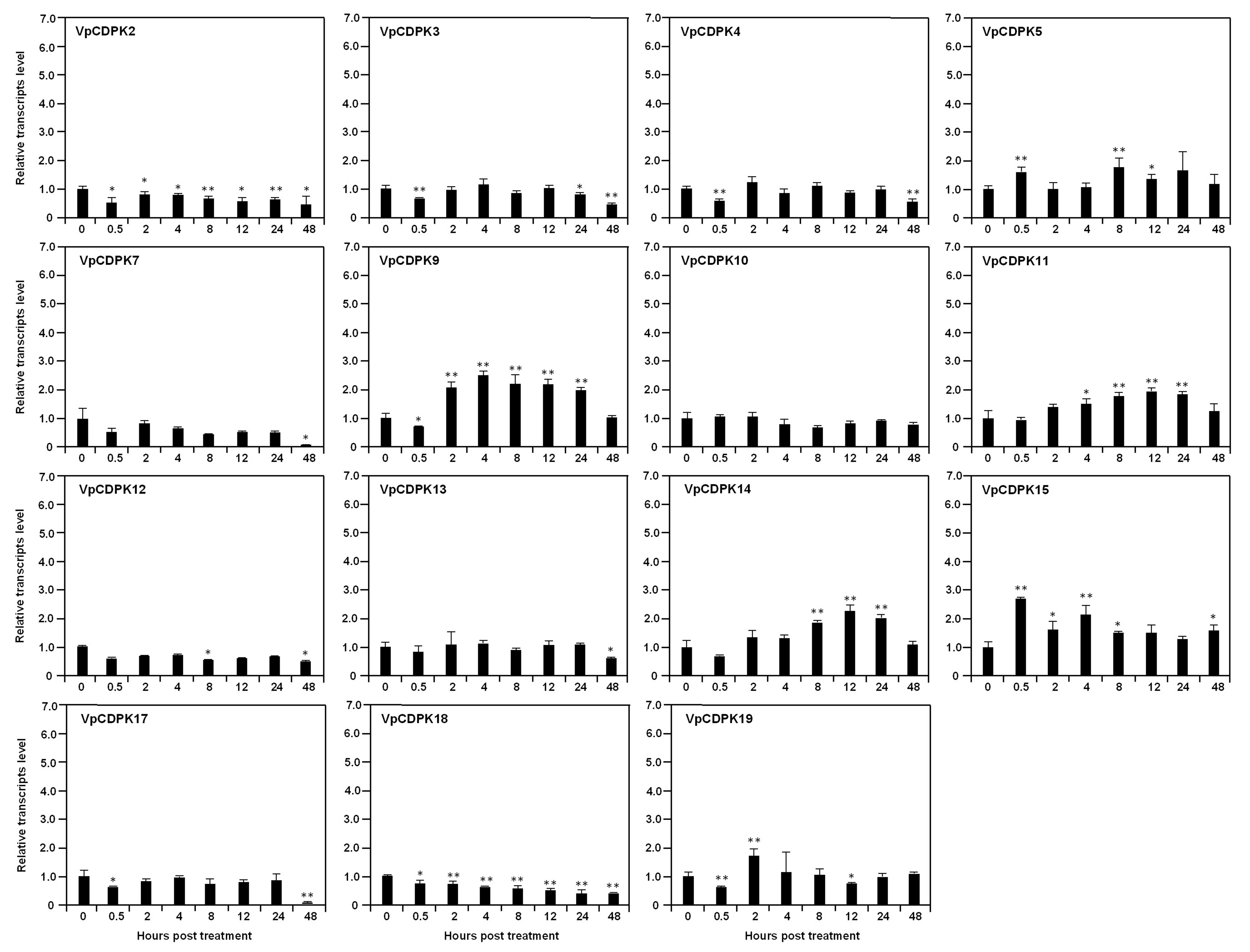

Supplement: Additional file 5: — Detailed expression profiling of the remaining 15 VpCDPK genes under 4°C treatment. Detailed expression levels were measured by RT-qPCR. Actin1 (GenBank Accession number AY680701) was used as an internal control. The experiments were repeated three times and gave consistent results. Mean values and SDs were obtained from three biological and three technical replicates. The data were showed as mean value ± SD. * and ** represent statistically significant (p<0.05) or highly significant (p<0.01), respectively. [file 12870_2015_552_MOESM5_ESM.tiff]

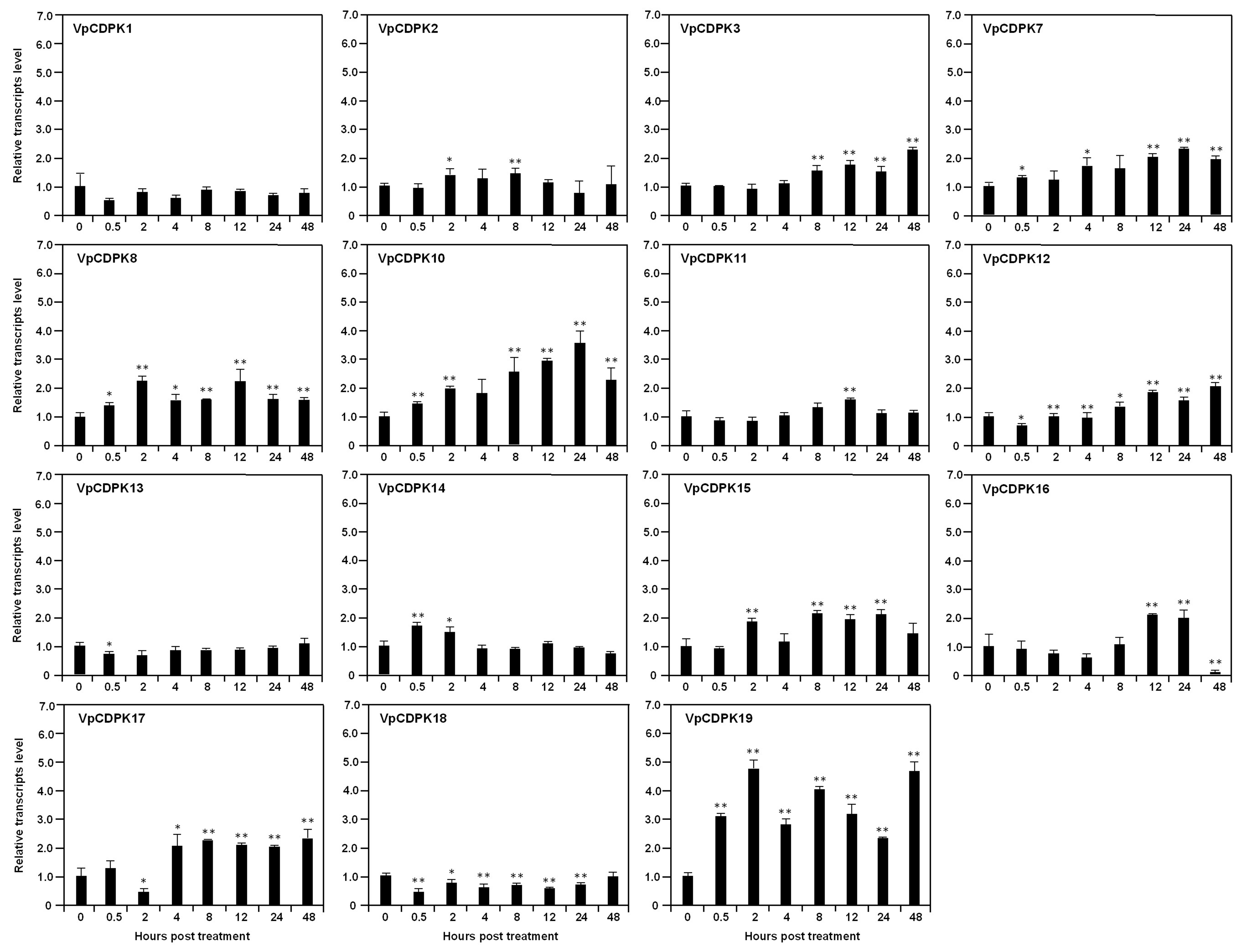

Supplement: Additional file 6: — Detailed expression profiling of the remaining 15 VpCDPK genes under 42°C treatment. Detailed expression levels were measured by RT-qPCR. Actin1 (GenBank Accession number AY680701) was used as an internal control. The experiments were repeated three times and gave consistent results. Mean values and SDs were obtained from three biological and three technical replicates. The data were showed as mean value ± SD. * and ** represent statistically significant (p<0.05) or highly significant (p<0.01), respectively. [file 12870_2015_552_MOESM6_ESM.tiff]

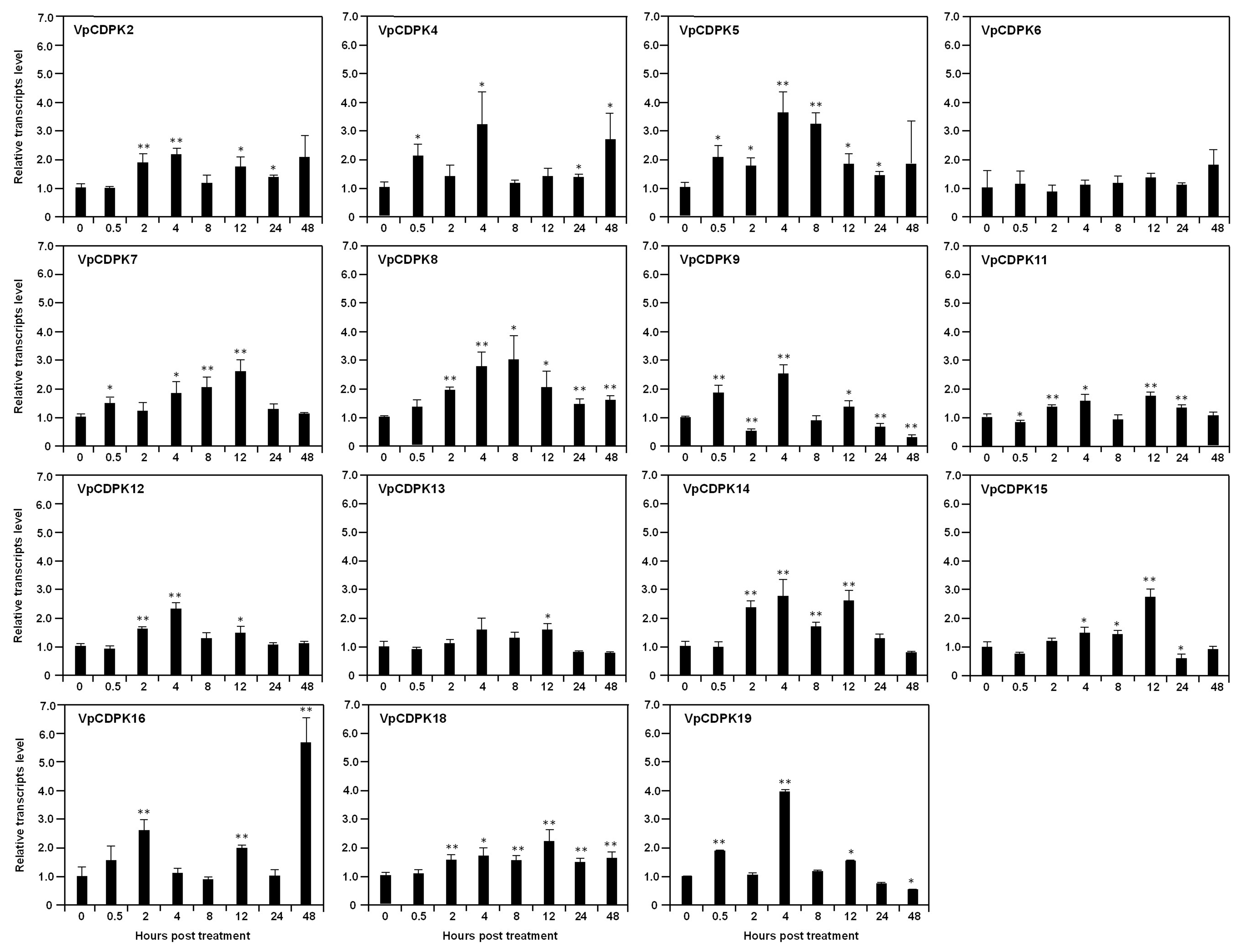

Supplement: Additional file 7: — Detailed expression profiling of the rest remaining VpCDPK genes under ABA treatment. Detailed expression levels were measured by RT-qPCR. Actin1 (GenBank Accession number AY680701) was used as an internal control. The experiments were repeated three times and gave consistent results. Mean values and SDs were obtained from three biological and three technical replicates. The data were showed as mean value ± SD. * and ** represent statistically significant (p<0.05) or highly significant (p<0.01), respectively. [file 12870_2015_552_MOESM7_ESM.tiff]

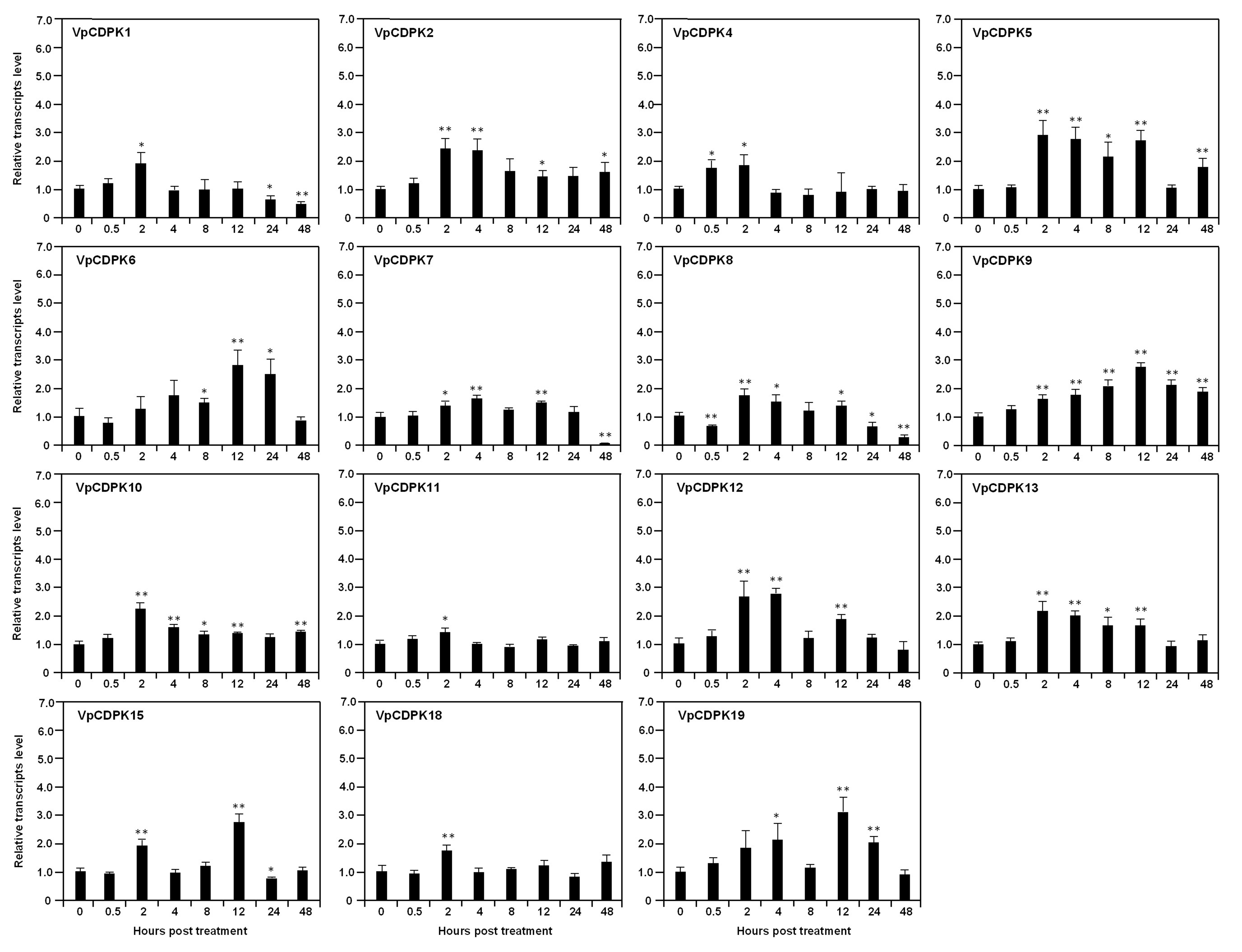

Supplement: Additional file 8: — Detailed expression profiling of the remaining 15 VpCDPK genes under SA treatment. Detailed expression levels were measured by RT-qPCR. Actin1 (GenBank Accession number AY680701) was used as an internal control. The experiments were repeated three times and gave consistent results. Mean values and SDs were obtained from three biological and three technical replicates. The data were showed as mean value ± SD. * and ** represent statistically significant (p<0.05) or highly significant (p<0.01), respectively. [file 12870_2015_552_MOESM8_ESM.tiff]

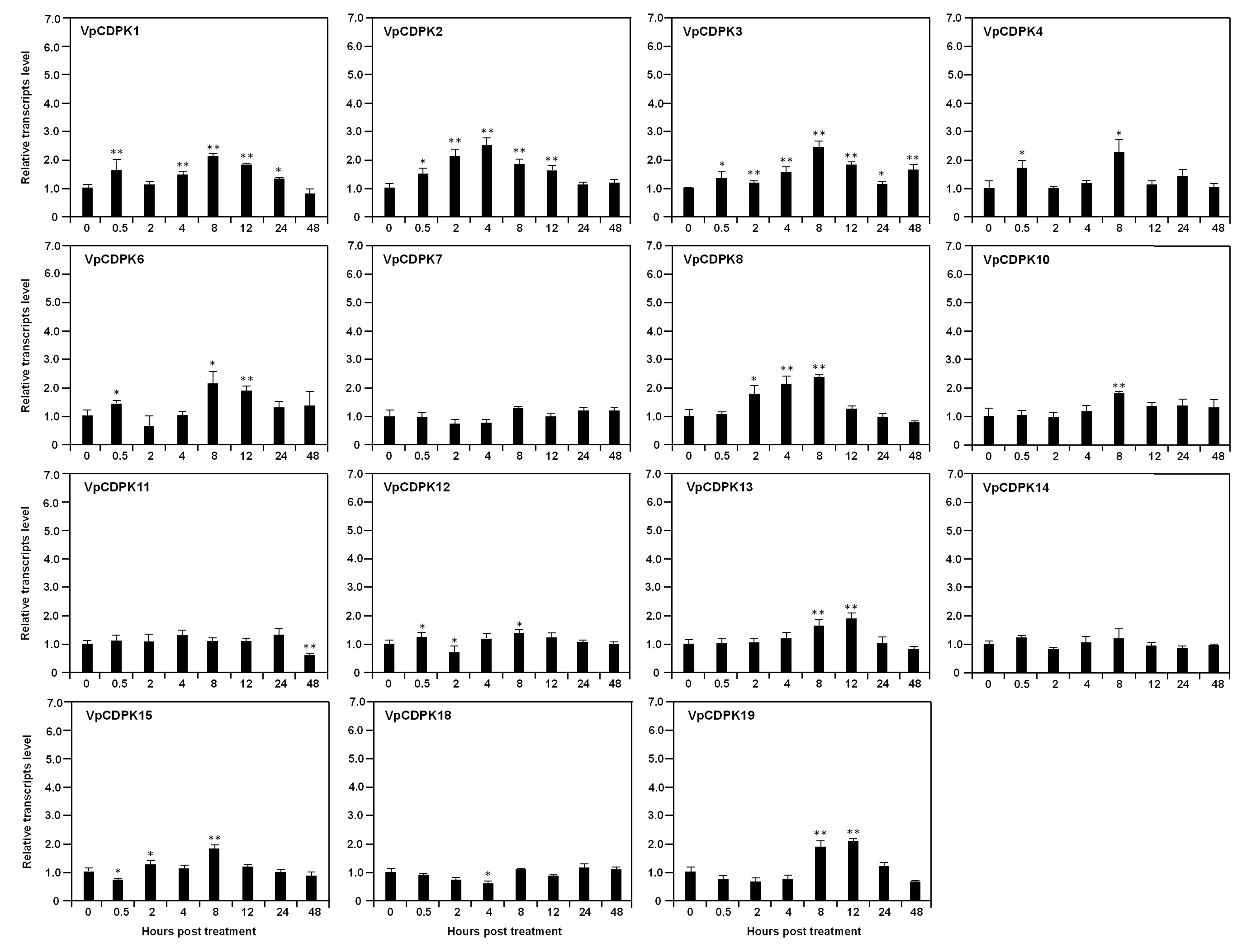

Supplement: Additional file 9: — Detailed expression profiling of the remaining 15 VpCDPK genes under MeJA treatment. Detailed expression levels were measured by RT-qPCR. Actin1 (GenBank Accession number AY680701) was used as an internal control. The experiments were repeated three times and gave consistent results. Mean values and SDs were obtained from three biological and three technical replicates. The data were showed as mean value ± SD. * and ** represent statistically significant (p<0.05) or highly significant (p<0.01), respectively. (TIFF 4982 kb) [file 12870_2015_552_MOESM9_ESM.tiff]

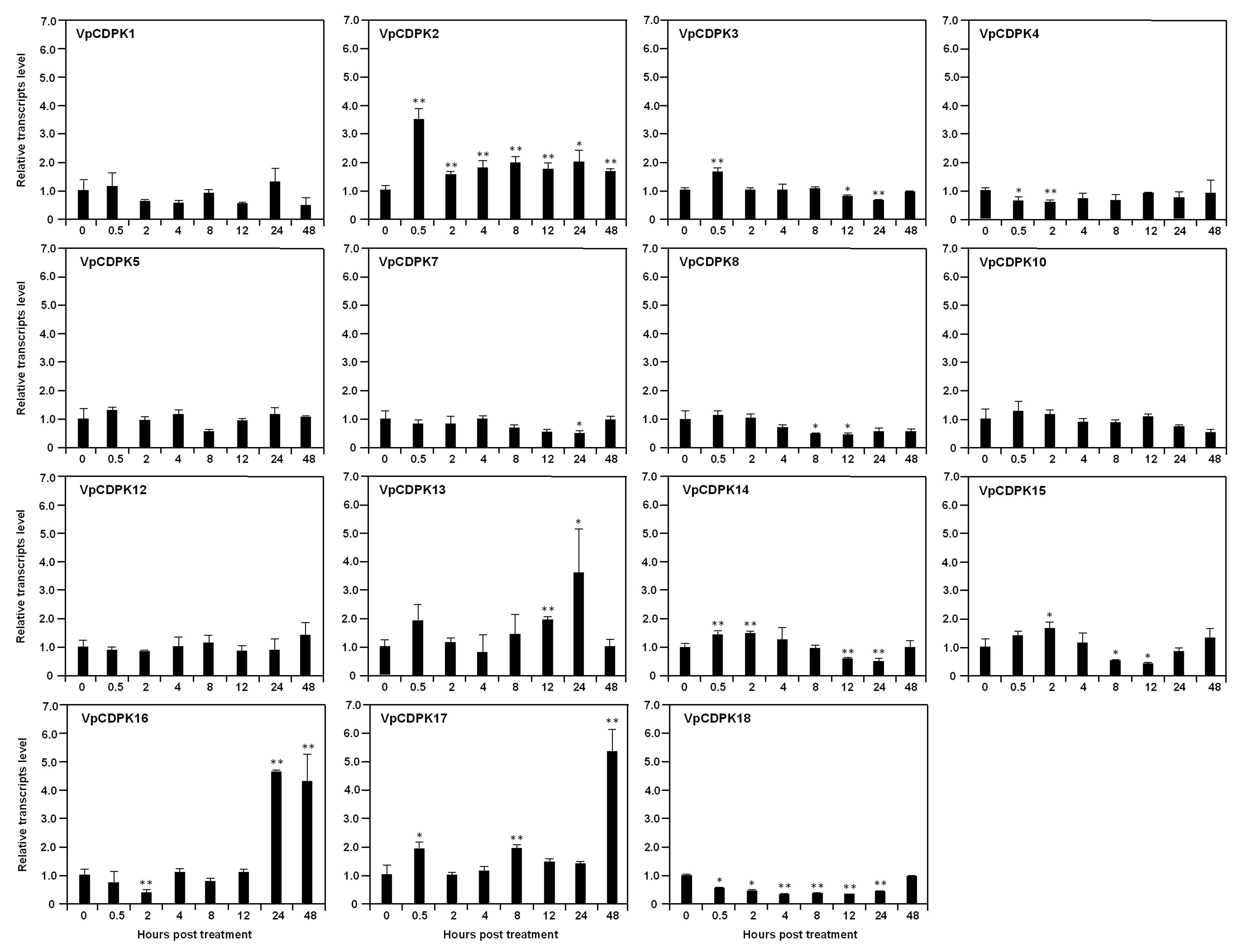

Supplement: Additional file 10: — Detailed expression profiling of the remaining 15 VpCDPK genes under ethylene treatment. Detailed expression levels were measured by RT-qPCR. Actin1 (GenBank Accession number AY680701) was used as an internal control. The experiments were repeated three times and gave consistent results. Mean values and SDs were obtained from three biological and three technical replicates. The data were showed as mean value ± SD. * and ** represent statistically significant (p<0.05) or highly significant (p<0.01), respectively. [file 12870_2015_552_MOESM10_ESM.tiff]
