# Supplementary material for: Genome-wide Identification and Expression Analysis of the CDPK Gene Family in Grape, Vitis spp
Source: BMC Plant Biol. 2015 Jun 30;15:164. doi: 10.1186/s12870-015-0552-z (PMC4485369; doi:10.1186/s12870-015-0552-z)
Supplement: Additional file 11: — Primers used in this study. [file 12870_2015_552_MOESM11_ESM.pdf]

Table S1. The Primers used in this study.

**Primer name**	**Sequence (5'-3')**[1]	**Description**[2]
VpCDPK1qF	CCTGTCGTCCATCGCCCTCTT	*VpCDPK1* (QRT)
VpCDPK1qR	CACCTCCCTCCGCACATCCTC	*VpCDPK1* (QRT)
VpCDPK2qF	GCAACTCTGACCGCTCAAATTCC	*VpCDPK2* (QRT)
VpCDPK2qR	TGGTTGGACTAAGGATGGATACACG	*VpCDPK2* (QRT)
VpCDPK3qF	AACGCCGCAGCCACCAAGA	*VpCDPK3* (QRT)
VpCDPK3qR	ACCGATTCCTGTCTCCCTCCTG	*VpCDPK3* (QRT)
VpCDPK4qF	CTGCATTTCCATGCCTGCTAAGG	*VpCDPK4* (QRT)
VpCDPK4qR	GATCGTCACCGATTTCCGAAACAC	*VpCDPK4* (QRT)
VpCDPK5qF	CAACTGGGCGTGACATTTCTCTCA	*VpCDPK5* (QRT)
VpCDPK5qR	GCAGGCATACTTTTCGTTTGTGGAT	*VpCDPK5* (QRT)
VpCDPK6qF	TCCACTCCTGCTGCTTCATCCG	*VpCDPK6* (QRT)
VpCDPK6qR	GGGTAACACCAAACTGCCCGC	*VpCDPK6* (QRT)
VpCDPK7qF	TCCGTTCTCCATCCTTCCCTCG	*VpCDPK7* (QRT)
VpCDPK7qR	TGTGGGTTTTGATGGTGCTCCTG	*VpCDPK7* (QRT)
VpCDPK8qF	GGCTTTCCAGTGCAGGGCTTC	*VpCDPK8* (QRT)
VpCDPK8qR	CCATTCGCCTTCTCCACGCA	*VpCDPK8* (QRT)
VpCDPK9qF	GTTCCCGAGCATGGATTGTTTGA	*VpCDPK9* (QRT)
VpCDPK9qR	CGTTTCGCTGACACCTTCTCCG	*VpCDPK9* (QRT)
VpCDPK10qF	AGCCGTTGCCAGGGAGGATG	*VpCDPK10* (QRT)
VpCDPK10qR	TCAACACCGTGATCGTCTTCTTGC	*VpCDPK10* (QRT)
VpCDPK11qF	ATGGGTAATTGCTGCGTGAC	*VpCDPK11* (QRT)
VpCDPK11qR	TTGCTCCCACCATTTCCCTGATTC	*VpCDPK11* (QRT)
VpCDPK12qF	GGAGGTGGTGGTGGAAGGAATC	*VpCDPK12* (QRT)
VpCDPK12qR	GTGGTTGGTGGTAAGTCTGTGTTGG	*VpCDPK12* (QRT)
VpCDPK13qF	GTTTCTTCCAATCTGTGTCGGCTG	*VpCDPK13* (QRT)
VpCDPK13qR	GTGGCTCATCCATAGTTTCTCCATTC	*VpCDPK13* (QRT)
VpCDPK14qF	CCAATCCATACGCCGACGACCA	*VpCDPK14* (QRT)
VpCDPK14qR	GCGACCCAGCACGTACTTGTCC	*VpCDPK14* (QRT)
VpCDPK15qF	CTCTTCTTCTTCCTCCTCTGATGGTG	*VpCDPK15* (QRT)
VpCDPK15qR	AGGTGGGATGTCCAATGACTCTTTG	*VpCDPK15* (QRT)
VpCDPK16qF	GCTTCTTGAATCCTTGTGGGCTG	*VpCDPK16* (QRT)
VpCDPK16qR	TCCTCCCTCAGTCTTGGTGTTGC	*VpCDPK16* (QRT)
VpCDPK17qF	TAAGCAAAGGCAAGAGGGGTTCAG	*VpCDPK17* (QRT)
VpCDPK17qR	CACGCTTTTCGGGTAATTGGT	*VpCDPK17* (QRT)
VpCDPK18qF	CTTTCTACCAACACCCAAACCACCA	*VpCDPK18* (QRT)
VpCDPK18qR	TGAATTGGCTGGCGAGTTCTTGA	*VpCDPK18* (QRT)
VpCDPK19qF	CGTAACCGTGGCAGCGAGAAAC	*VpCDPK19* (QRT)
VpCDPK19qR	CCGCTTGGGTCCTCATACACG	*VpCDPK19* (QRT)
VpActF	GCTGGATTCTGGTGATGGTGTG	Grape Actin
VpActR	TCCCGTTCAGCAGTAGTGGTG	Grape Actin
VpCDPK2XbaF	AAA**TCTAGA**ATGGGCAACACATGCCGGG	*VpCDPK2* (C,E)

VpCDPK2XhoR	AAA**CTCGAG**AAAAGCTCCTGGTGCATCTCTCAT	*VpCDPK2* (C,E)
VpCDPK3XbaF	AAA**TCTAGA**ATGGGGGCGTGCCTCTCC	*VpCDPK3* (C,E)
VpCDPK3XhoR	AAA**CTCGAG**AATTTTCCGAGGATTCCGAAAAC	*VpCDPK3* (C,E)
VpCDPK5XbaF	AAA**TCTAGA**ATGGGAAATTGCTGTGCATCA	*VpCDPK5* (C,E)
VpCDPK5XhoR	AAA**CTCGAG**TTGCCCATCCTTCCCATCC	*VpCDPK5* (C,E)
VpCDPK9XbaF	AAA**TCTAGA**ATGGGGAATAACTGTGTGGGATC	*VpCDPK9* (C,E)
VpCDPK9XhoR	AAA**CTCGAG**ATAGACTGGTAGCGGCTGCCTAA	*VpCDPK9* (C,E)
VpCDPK10XbaF	AAA**TCTAGA**ATGGGGAACTGTTGCAGATCTCC	*VpCDPK10* (C,E)
VpCDPK10XhoR	AAA**CTCGAG**CTCATTCCCCAAGTTTAGAGAACCA	*VpCDPK10* (C,E)
VpCDPK11XbaF	AAA**TCTAGA**ATGGGTAATTGCTGCGTGACC	*VpCDPK11* (C,E)
VpCDPK11XhoR	AAA**CTCGAG**TGGCCTAACTTCCAATGATCCATC	*VpCDPK11* (C,E)
VpCDPK19XbaF	AAA**TCTAGA**ATGGGACAGGAAACAAGGAGAC	*VpCDPK19* (C,E)
VpCDPK19XhoR	AAA**CTCGAG**TAGTGGGCGGAGAGAGGAGAGG	*VpCDPK19* (C,E)

1 Restriction sites are indicated in bold.

2 The type of experiment for which the primers were used is indicated in brackets (C: cloning, QRT: RT-qPCR, E: expression analysis).
